# Multidomain analyses of a longitudinal human microbiome intestinal cleanout perturbation experiment

**DOI:** 10.1371/journal.pcbi.1005706

**Published:** 2017-08-18

**Authors:** Julia Fukuyama, Laurie Rumker, Kris Sankaran, Pratheepa Jeganathan, Les Dethlefsen, David A. Relman, Susan P. Holmes

**Affiliations:** 1 Statistics Department, Stanford University, Stanford, California, USA; 2 Department of Microbiology & Immunology, Stanford University School of Medicine, Stanford, California, USA; 3 Harvard Medical School, Boston, Massachusetts, USA; 4 Department of Medicine, Stanford University School of Medicine, Stanford, California, USA; 5 Infectious Diseases Section, Veterans Affairs Palo Alto Health Care System, Palo Alto, California, USA; University of Washington, UNITED STATES

## Abstract

Our work focuses on the stability, resilience, and response to perturbation of the bacterial communities in the human gut. Informative flash flood-like disturbances that eliminate most gastrointestinal biomass can be induced using a clinically-relevant iso-osmotic agent. We designed and executed such a disturbance in human volunteers using a dense longitudinal sampling scheme extending before and after induced diarrhea. This experiment has enabled a careful multidomain analysis of a controlled perturbation of the human gut microbiota with a new level of resolution. These new longitudinal multidomain data were analyzed using recently developed statistical methods that demonstrate improvements over current practices. By imposing sparsity constraints we have enhanced the interpretability of the analyses and by employing a new adaptive generalized principal components analysis, incorporated modulated phylogenetic information and enhanced interpretation through scoring of the portions of the tree most influenced by the perturbation. Our analyses leverage the taxa-sample duality in the data to show how the gut microbiota recovers following this perturbation. Through a holistic approach that integrates phylogenetic, metagenomic and abundance information, we elucidate patterns of taxonomic and functional change that characterize the community recovery process across individuals. We provide complete code and illustrations of new sparse statistical methods for high-dimensional, longitudinal multidomain data that provide greater interpretability than existing methods.

## Author summary

Complex dynamics of microbial communities underlie their essential roles in health and disease. To maintain or restore healthy states, we must better understand the nature and basis of stability in the gut microbiota, under normal and perturbed conditions. Stability, resilience, and response to perturbation are central topics in community ecology. Extreme perturbations such as near-complete loss of biomass from a system can reveal factors that influence community structure. Recognizing the return to baseline diversity and abundances of biomarkers in community-wide recovery after a disturbance enables us to understand the basic pillars of resilience that contribute to human health. We have designed a densely sampled longitudinal experiment in human volunteers using transient non-inflammatory diarrhea as the perturbation. In order to uncover the essential players in the recovery process, we have tailored new advances in ribosomal sequence variant detection and sparse multidomain analytics that incorporate phylogenetic structure. We show sparse meaningful multidimensional projections that exhibit the essential features in resilient recovery. This work shows how a carefully designed longitudinal study combining denoised ribosomal RNA sequence variants and metagenomic data can inform the taxa and processes involved in the recovery from loss of large proportions of intestinal biomass.

## Introduction

The complex, dynamic microbial communities of the human body play essential roles in health and disease. For example, the human gut microbiota contributes to digestion, defense against pathogens, biosynthesis of essential molecules, metabolic homeostasis, and regulation of the immune system [1–3], but has also been implicated in malnutrition, obesity, diabetes, heart disease, cancer, and autoimmune diseases [4–10]. To maintain or restore healthy states, we must better understand the nature and basis of stability in the gut microbiota, under normal and perturbed conditions.

Stability, resilience, and response to perturbation are central topics in community ecology [11]. Extreme perturbations of a system, such as near-complete loss of biomass, are studied both to reveal factors that influence community structure, and as important phenomena in their own right. For example, Fisher et al. (1982) [12] examined the response of a desert creek ecosystem to flash flooding, with results that matched some but not all of Odum’s theoretical expectations about ecological succession [13]. Particular findings included a return to baseline values of community-wide measures such as diversity indices even while individual taxa were continuing to recover from the disturbance. They also found that the specific characteristics of organisms (e.g., the rapid post-flood emergence of motile diatoms buried in sediment, the existence of a nonaquatic adult dipteran stage that was not vulnerable to washout) influenced community composition during recovery in ways that were not evident from the study of unperturbed intervals [12].

As part of an ongoing study of human microbiota stability and resilience, we created a flash flood-like disturbance in the human gut by inducing acute, transient non-inflammatory diarrhea using a common clinically-relevant iso-osmotic agent, thereby eliminating the vast majority of gastrointestinal biomass. Induced, iso-osmotic diarrhea (IIOD) differs qualitatively from the less extreme and more selective, inhibitory and stimulatory action of antibiotics [14] and of diet supplementation [15, 16] that have more frequently been investigated as disturbances of the gut microbiota. Comparison of different types of perturbation is necessary to understand whether the traits of organisms or communities that affect resilience are specific to each type of perturbation, or act more generally. In addition, understanding the effects of IIOD on the gut microbiota has practical importance because it is a common clinical procedure (approximately 14 million persons in the United States were subjected to this disturbance in 2013 as preparation for colonoscopy [17]). Furthermore, studying the effects of diarrhea *per se* on the gut microbiota is relevant for our understanding of infectious diarrheal disease, which remains a major cause of mortality worldwide [18].

Several previous studies have investigated the effects of induced diarrhea on the human gut microbiota using 16S rRNA gene surveys that provide a more complete representation of the community than the older culture-based techniques [19]. Most studies recruited participants who experienced both induced diarrhea and colonoscopy for screening or diagnostic purposes [20–22], one study examined induced diarrhea without colonoscopy in healthy subjects [23] and one study induced less extreme diarrhea over several days intended to represent the physical effects of infectious diarrhea in the absence of an infectious agent [24]. Sampling strategies varied considerably between these studies, but none collected samples with sufficient frequency before or after the induced diarrhea to assess what day-to-day changes might be expected in the absence of deliberate perturbation. Furthermore, samples representing the perturbed state were separated by at least one week from any follow-up samples, so a detailed time course of gut microbiota recovery could not be investigated in these studies. We designed our sampling regime both to compare the effect of IIOD to the routine temporal variability of the gut microbiota in the same subject and to assess the timecourse of community recovery after IIOD.

Some recent studies of the human gut microbiota have continued to rely on 16S rRNA gene surveys alone [25, 26], but it is increasingly common to combine such surveys with additional high throughput, culture independent methods, such as metagenomic ‘shotgun’ sequencing [27, 28], or metabolomics [29, 30]. While all these methods provide a tremendous amount of information about microbial communities in their natural state, they present new and different challenges for data analysis and interpretation. We take the opportunity of analyzing our new human gut microbiota dataset to highlight useful recent advances in statistical methods which have yet to become widely adopted in microbiome studies.

Two related challenges recognized soon after the application of next-generation sequencing to 16S rRNA gene surveys are the high dimensionality of the data (hundreds or thousands 16S rRNA sequence variants identified per sample) and the need to distinguish sequencing errors from genuine biological variation. A common response to both issues has been the application of *ad hoc* clustering methods that sweep both biological variants and error-containing sequences into bins defined by a fixed similarity threshold (known as Operational Taxonomic Units or OTUs); such an approach loses information by obscuring the existence of sequence variants that may represent ecologically distinct microbial strains [31]. In contrast, an explicit data-derived error model of Illumina amplicon sequencing allows likely ribosomal sequence variants (RSVs) to be distinguished both from each other and from errors, with a resolution as fine as single nucleotide differences, as demonstrated by the recent DADA2 package [32].

Once the sequence data are represented as an abundance matrix, with samples as rows and RSVs as columns, they become amenable to statistical scrutiny. However, these data present a unique set of methodological challenges; in response we present solutions based on adaptations of existing techniques or the introduction of new techniques. The first central challenge is high-dimensionality. After preliminary preprocessing, we have 419 samples and have measured 2611 RSVs and 2798 genes across these samples. Traditional methods can become unreliable and uninterpretable in this regime, where there are more measured features than samples. A second difficulty is interpretation in terms of phylogenetic units during analysis. There are few options for ordination that account for the known evolutionary relatedness between RSVs, and these methods are generally inflexible. However, incorporation of this structure leads to more informative results. Finally, standard techniques are not well-suited to simultaneous study of multiple data sources. Experiments that collect multidomain data on the same samples provide more interesting views of samples, by describing them from several angles. When such complementary data are available, it becomes interesting to characterize covariation across sources [33]. At present, there are relatively few methods designed for this purpose.

To address these challenges, we repeatedly invoke a few key statistical principles. The first is that statistical methods can be improved by explicitly encoding known structure, for example, through informative priors or clever featurization. This principle motivates two methods that we introduce in this work—adaptive generalized principal components analysis (agPCA) [34, 35] and tree-based sparse linear discriminant analysis (LDA). By guiding statistical methods with domain knowledge—for example, about the phylogenetic relatedness of RSVs—we can typically obtain more useful results. A second principle is that *ℓ*^1^-regularization can address high-dimensionality in a way that facilitates interpretation. Indeed, regularization is foundational in modern high-dimensional statistics, and among regularization methods, *ℓ*^1^ constraints allow for the most convenient descriptions, because they induce sparsity [36]. “Sparsity” in this context means that a limited number of features, for us, either RSVs or gene ontology (GO) terms, are picked out as important for explaining the structure in the data. This form of regularization is used in both our tree-regularized supervised LDA and unsupervised sparse canonical correlation analysis (sCCA).

By implementing an intensive longitudinal sampling scheme that extended well before and after IIOD, we sought to place this perturbation to the human gut microbiota in the context of routine temporal variability. We characterized both the composition and functional potential of the gut community in eight individuals, analyzing the data with these new statistical methods and demonstrated improvements over current practice. Specifically, we pursued the following study aims: 1) determine whether and how quickly the gut microbiota demonstrates resilience after an IIOD perturbation, 2) elucidate patterns of taxonomic and functional change that characterize the community recovery process across individuals, and 3) innovate and apply statistical methods for high-dimensional, longitudinal multidomain data that provide greater interpretability than existing methods.

## Materials and methods

### Ethics statement

The research was approved by an Administrative Panel for the Protection of Human Subjects (Institutional Review Board) of Stanford University (protocol 25268). All subjects were properly informed of the risks and benefits of this study, and then signed an approved, written consent form.

### Experimental design

An unequally spaced time point design for longitudinal data with perturbations was created according to recommendations in the statistical design literature [37, 38].

Demographic and life history factors such as gender, race and BMI, often used to stratify human populations in epidemiological studies generally have only small effects on the gut microbiota [39]. Note that a within-subject comparison of perturbed and unperturbed samples was possible because our longitudinal sampling design establishes the baseline temporal variability; insufficient sampling would increase the risk of mistaking routine temporal variability for a treatment effect. We show simulations that prove that crossover longitudinal sampling with baseline computations are more powerful than parallel designs in the supporting information (S5 and S6 Figs).

The response of the human gut microbiota to IIOD was evaluated by collecting fecal samples from eight healthy participants for approximately ten weeks before and ten weeks after a one-day IIOD event. IIOD is commonly used to clear the bowel prior to colonoscopy; the perturbation in this study exactly reflects a commonly-used clinical protocol for bowel preparation. On the morning of the perturbation, participants were instructed to drink ~300 mL of a solution (GoLytely) containing polyethylene glycol (PEG) and electrolytes every 10 minutes (up to 4L total) until their diarrhea was clear and watery. Samples were requested once per week every week, except during the week before and the week after IIOD when daily samples were requested. Five consecutive daily samples were also collected at least 6 weeks prior to IIOD. DNA was extracted from the stool samples and used for amplicon sequencing of the V4 region of the 16S rRNA gene as well as ‘shotgun’ metagenomic sequencing. The data were analyzed to reveal community composition and functional profiles, in an attempt to characterize the immediate response to IIOD, and to assess long-term effects of the perturbation.

### Participants and sampling protocol

Healthy nonpregnant adults were recruited from the Stanford community, excluding individuals with chronic disease, hospitalization or antibiotic use in the previous 6 months, immunizations or international travel in the previous 4 weeks, or routine use of any prescription medication except birth control or hormone replacement therapy. Characteristics of the eight participants who completed the sampling protocol are summarized in Table 1. Participants collected ~2g stool samples at home, which were frozen immediately without preservative in home freezers. Samples were transferred without thawing to −80°C storage in the laboratory approximately every 3 weeks.

**Table 1:** Characteristics of study participants.

ID Code	Sex	Age	Racial Identification	BMI
AAA	Female	31	Caucasian	20.0
AAB	Male	52	Caucasian	25.8
AAD	Female	20	Caucasian	19.6
AAF	Male	27	Caucasian	20.8
AAG	Male	27	Caucasian	23.1
AAI	Female	31	Caucasian and Asian	21.3
AAN	Female	21	Caucasian	23.5
AAP	Female	21	Caucasian	20.0

**Fig 1:**
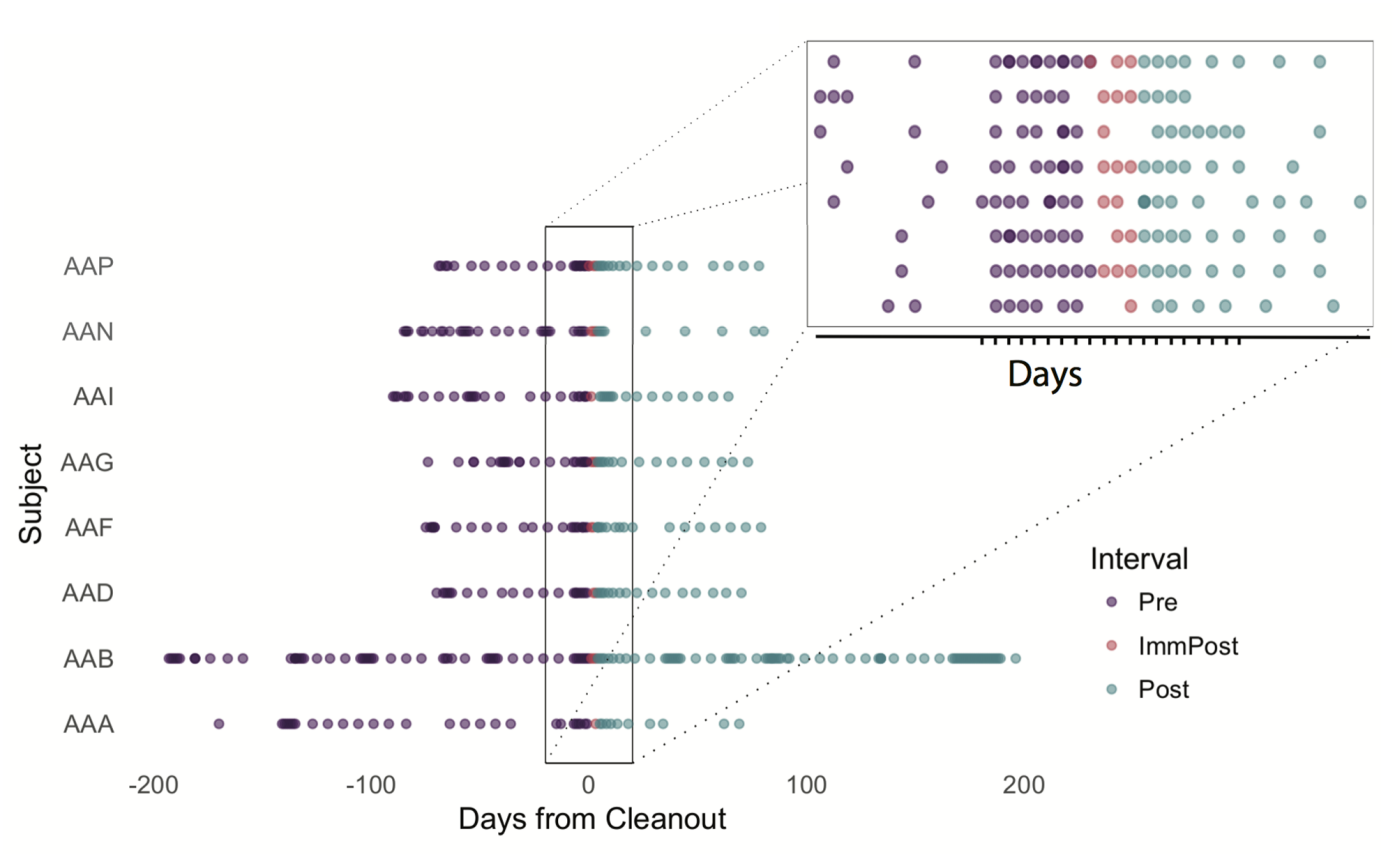
The sampling times for each participant in the study, relative to the time of the IIOD event. ImmPost samples are those taken within 3 days of IIOD. Note the denser sampling in the period immediately preceding and following this event.

A total of 419 fecal samples were collected; the timing of samples relative to IIOD for each participant is shown in Fig 1. Post-disturbance sampling began with the first bowel movement after IIOD in all subjects, which ranged from 1–3 days after IIOD. Some intended daily samples were not collected because participants did not produce stool that day.

### Sample processing and DNA extraction

Samples were thawed at 4°C to a semi-solid state and ~250mg aliquots were transferred to wells of the PowerSoil -htp 96 Well Soil DNA Isolation Kit (MoBio). Extraction followed the manufacturer’s centrifugation protocol, with the following modifications: stool tubes were thawed in small batches to minimize time unfrozen, the deepwell extraction plate was cooled on dry ice during sample loading, and extraction plates were returned to −80°C for at least 1 hour after loading to ensure consistent freeze-thaw cycles across all samples. Bead solution and C1 solution were added upon removal from the freezer to begin extraction with a 10 min incubation at 65°C, followed by 20 min beadbeating with the recommended MM 400 device (Retsch). 6–12 extraction control blanks were included per extraction plate, as well as 52 replicate stool aliquots derived from 17 distinct samples.

### 16S rRNA gene sequencing

The V4 region of the 16S rRNA gene was amplified for sequencing using 515F and barcoded 806R primers as described by Caporaso et al. [40]. Triplicate 25 *μ*L PCR reactions using Hot MasterMix (5 Prime) with 3 *μ*L extracted DNA as template and 10*μ*g/*μ*L BSA were cycled as follows: denaturation at 94°C for 3 min, 25 cycles of 94°C/45s, 52°C/60s, 72°C/120s, final extension at 72°C for 10 min. PCR amplicon libraries were purified using the UltraClean-htp 96 Well PCR Cleanup Kit (MoBio). Amplicon libraries were quantified by fluorometry (Quant-iT dsDNA High Sensitivity Kit, Invitrogen) on a SynergyHT plate reader (BioTek) and combined in equimolar ratios into two pools. Pooled libraries were concentrated by ethanol precipitation and gel purified (QIAquick Gel Extraction Kit, Qiagen).

Each pool of V4 16S rRNA amplicons was sequenced (2x150 paired end) on one lane of a HiSeq2500 sequencer (Illumina) at the Carver Biotechnology Center of the University of Illinois, producing an average of 237,800 reads per sample, with sample depths varying from 42,200 to a maximum of 1,530,000, with a total of 365,093,804 reads produced for this study. The DADA2 sequence processing pipeline (version 1.1) as described in [32] was used to infer the set of ribosomal sequence variants (RSVs) present and their relative abundances across the samples. Rather than clustering amplicon sequencing reads into Operational Taxonomic Units (OTUs) at a fixed similarity threshold, DADA2 derives an abundance distribution of distinct Ribosomal Sequence Variants (RSVs), which may differ by only a single nucleotide, consistent with the observed sequence reads, based on data-derived rates of Illumina sequencing errors. Using read quality scores for the dataset, forward and reverse reads were truncated at 150bp and 130bp, respectively; other quality filtration parameters used DADA2 default values. Taxonomic assignment was performed on RSVs using the RDP classifier and reference dataset [41] following the workflow outlined in [42].

#### Metagenomics

Metagenomic sequencing libraries were prepared from DNA extracts at the Carver Biotechnology Center of the University of Illinois using HyperPlus kits (Kapa Biosystems). Beadbeating during DNA extraction resulted in many samples with median fragment size < 500 bp; in these cases size fractionation focused on removing fragments < 200 bp prior to library construction. For samples with substantial amounts of larger DNA fragments, size fractionation enriched for fragments 200–800bp. For 7 of the 8 subjects all samples of the subject were multiplexed sequencing (2 × 160 or 2 × 250 paired-end) on one lane of a HiSeq2500 sequencer (Illumina). The larger number of samples from subject AAB were sequenced on two lanes to retain approximate parity in sequence depth per sample.

Because most paired-end reads in a majority of samples overlapped, pairs were joined using the Usearch v8.1 fastq_mergepairs command [43], discarding merged sequences with length < 72, containing any ambiguous base calls, or with > 1 expected error based on corrected Illumina quality scores for the merged sequence. Unmerged reads were quality filtered using the Usearch fastq_filter command, truncating at quality score 12 and discarding reads of length < 72, containing any ambiguous base calls or > 1 expected error. The sensitive setting of bowtie v2.2.4 [44] was used to remove metagenomic reads mapping to the human genome (GRCh.38, [45]).

Filtered, human-screened, merged and unmerged reads were assembled jointly for all samples from a given subject using Megahit v1.0.1 [46] with parameters k-min = 19, k-max = 119, k-step = 20 and a minimum contig length of 300. Putative genes were predicted on contigs derived from each subject using MetaGeneMark with default parameters (gmhmmp v3.26 with MetaGeneMark_v1.mod [47]). Predicted amino acid sequences were compared to UniRef100 v2015_12 [48] using sensitive mode of Diamond blastp v0.8.1 [49] with the BLOSUM80 matrix [50], accepting the hit with highest bit score as the identity of a query if it had an e-value no greater than 10^−6^ and at least 50% sequence identity over at least 70% of the query length.

The concatenated set of merged and unmerged reads for each sample were mapped individually to the contigs assembled from that subject using the sensitive-local setting of bowtie2 with minimum mapping quality of 20. A custom Perl script cross-tabulated read counts (counting merged reads as 2) per contig per sample with predicted gene hits vs. UniRef100 to obtain counts per sample per UniRef gene ID. These counts were normalized per kb of contig length and for sample variability in both sequencing depth and average genome size as estimated by MicrobeCensus v1.0.7 [51].

### Incorporating phylogenetic information

To improve power to detect subtle effects and to increase interpretability, it is often useful to include information about the phylogenetic relationships between RSVs. We use phylogeny both in the supervised context, to find groups of RSVs which distinguish samples immediately after the cleanout from the rest, as well as in the unsupervised context, to obtain a low-dimensional representation of the samples where the axes are interpretable in terms of over- or under-representation of groups of related RSVs.

#### Unsupervised analysis

There are several methods available for dimensionality reduction of microbiome data that incorporate phylogenetic structure of the RSVs. Weighted and unweighted Unifrac [52, 53] are phylogenetic distances that are used in combination with multi-dimensional scaling (MDS) to obtain a low-dimensional representation of microbial communities. Although these distances account for phylogeny, they do not ensure that RSVs load smoothly on the MDS axes, so it is difficult to interpret MDS directions in terms of phylogenetically-related groups of RSVs.

Both Pardom [54] and more recently Washburne et al [55] propose multivariate methods that integrate the phylogenetic distance into a multivariate generalized PCA or factor analysis. Purdom [54] proposed using double principal coordinates analysis (DPCoA) [56] as a dimensionality reduction method for general distances between RSV features whose phylogenetic relationships are known. DPCoA has the advantage over Unifrac of producing interpretable axes, while the distances implied by DPCoA remain very similar to those given by weighted Unifrac [57]. However, the axes given by DPCoA are usually smooth at the phylum level, and so the interpretation of the relative sample positions is in terms of the relative abundances of various phyla. This is not always desirable, as it might be the case that smaller groups of RSVs, say at the genus level, are the relevant units of analysis.

To deal with some of these issues, we have developed a new method which we call adaptive generalized PCA (gPCA). The mathematical details and justification are given in a separate paper and R package available on CRAN [35, 58]. The method was developed in the context of analyzing the data in the present study. We wanted to obtain a low-dimensional representation of the samples in which the axes were interpretable at a finer phylogenetic scale than what is available to us in DPCoA. Adaptive gPCA defines a family of projections of the data which interpolate between DPCoA (which emphasizes structure at a coarse phylogenetic level) and PCA (which does not take into account the phylogeny), which is equivalent to considering all phylogenetic information to be contained at the finest taxonomic scale. This family of projections corresponds to putting tree-structured priors of different strengths on the data, and the strength of this prior can be estimated from the data. In practice, this leads to low-dimensional representations of the data which are interpretable at a finer phylogenetic scale than those resulting from DPCoA.

#### Supervised analysis

It is also informative to perform a supervised analysis that includes phylogenetic information. Two examples of this are constrained DPCoA [59], which generalizes DPCoA to the problem of discriminating between classes, and kernel-penalized regression [60]. However, while they incorporate phylogeny, these methods do not induce sparsity in scores, which would facilitate identification of a small subset of related RSVs that discriminate between classes.

Therefore, in this work, we perform a modified version of sparse discriminant analysis that gives both sparsity and phylogenetic structure. First we created the phylogenetic tree following the standard workflow for RSVs as documented in [42], fitting a maximum likelihood tree with a generalized reversible Markovian model with Gamma rates.

We create two sets of features, one corresponding to leaves on the phylogenetic tree, and the other corresponding to nodes. For each leaf on the tree, the corresponding feature value is the variance-stabilized RSV abundance. For each node on the tree, the corresponding feature value is the sum of the variance-stabilized RSV abundances for all RSV leaves descending from that node. These are then centered and used as input to sparse discriminant analysis. We used the sparse discriminant analysis implementation in the R package sparseLDA [61]. The sparsity parameter was set by cross-validation, holding out one subject at a time.

Note that the use of a sparse supervised method (in our case sparse instead of standard LDA) is important when using both node features and leaf features at the same time. Because node features are exactly linear combinations of leaf features and of each other, a model without a sparsity penalty would be unidentifiable—there would be an infinite number of solutions, all equally good, but with different coefficient values. The sparsity constraint by an *ℓ*^1^-penalty resolves this unidentifiability and allows use of both node and leaf features at the same time.

This method is inspired in part by the idea of evolutionary units, which were shown to provide a unifying framework for several measures of phylogenetic diversity and dissimilarity [62]. In this framework, an evolutionary unit is a branch on the phylogenetic tree (or a standardized portion thereof), and the abundance of each evolutionary unit is the sum of the abundances of the species that descend from it. As an example, the unweighted Unifrac distance is the proportion of evolutionary units which are not shared between two samples. We use evolutionary units as input to sparse discriminant analysis, but in contrast to the evolutionary units described in [62], we ignore branch lengths.

In the supplementary material, we compare this approach to the LEfSe method developed by [63] to integrate taxonomic and metabolic information through successive filtering. However, just using relative proportions of RSVs do not provide any significant differences. By multiplying the proportions by a million, a number of RSVs become significant (see the results as displayed in S9 Fig). The choice to filter and loosen stringency by multiplying by a large factor inflates significance, however statistical guarantees no longer hold. Integrating taxonomic information into LEfSe while accounting for taxonomic levels could be done using [64], we have not done this here as the goal of this supplement is to compare with standard procedures that are already being used by practionners.

## Results

### Between-subject variation

**Fig 2:**
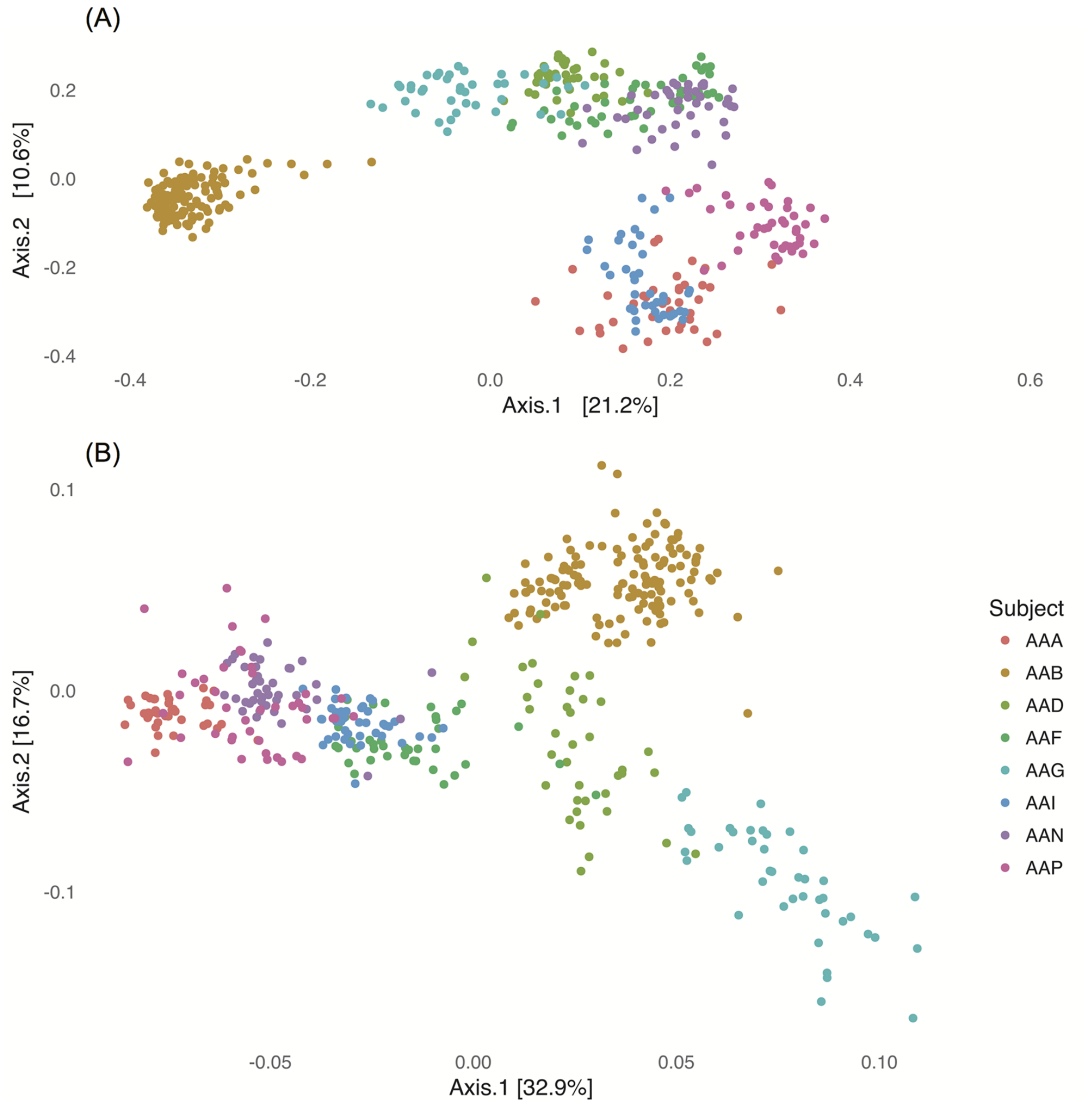
The first two axes of the multidimensional scaling (MDS) projection using Bray-Curtis distances shown in the top figure (A) and the agPCA projection in (B). Both methods demonstrate clear intersubject differences.

Bray-Curtis dissimilarity was computed between all possible sample pairs and MDS was used to obtain a low-dimensional representation of these dissimilarities. The results are shown in Fig 2A, where the main effect is the difference across subjects. In other words, between-subject distances tend to be larger than within-subject distances. With the Bray-Curtis ordination, the pre-cleanout and post-cleanout samples do not show any systematic differences, as can be seen in S3 Fig.

Community compositions in the days immediately surrounding the perturbation are displayed in supplementary S1 Fig. The analogous figure at the weekly level, is given in S2 Fig. The differences in composition across subjects is clearly evident, reinforcing the result of Fig 2. Further, subjects AAD, AAF, AAG, and, to some extent, AAI exhibit decreases in Ruminococceae and Lachnospiraceae, though to differing degrees. These subjects also see an increase in the proportions of either Bacteroidaceae or Prevotellaceae in the days following the perturbation.

The between-subject variation strongly justifies the decision to use a design in which each subject is their own control.

### IIOD effect highlighted by adaptive gPCA

**Fig 3:**
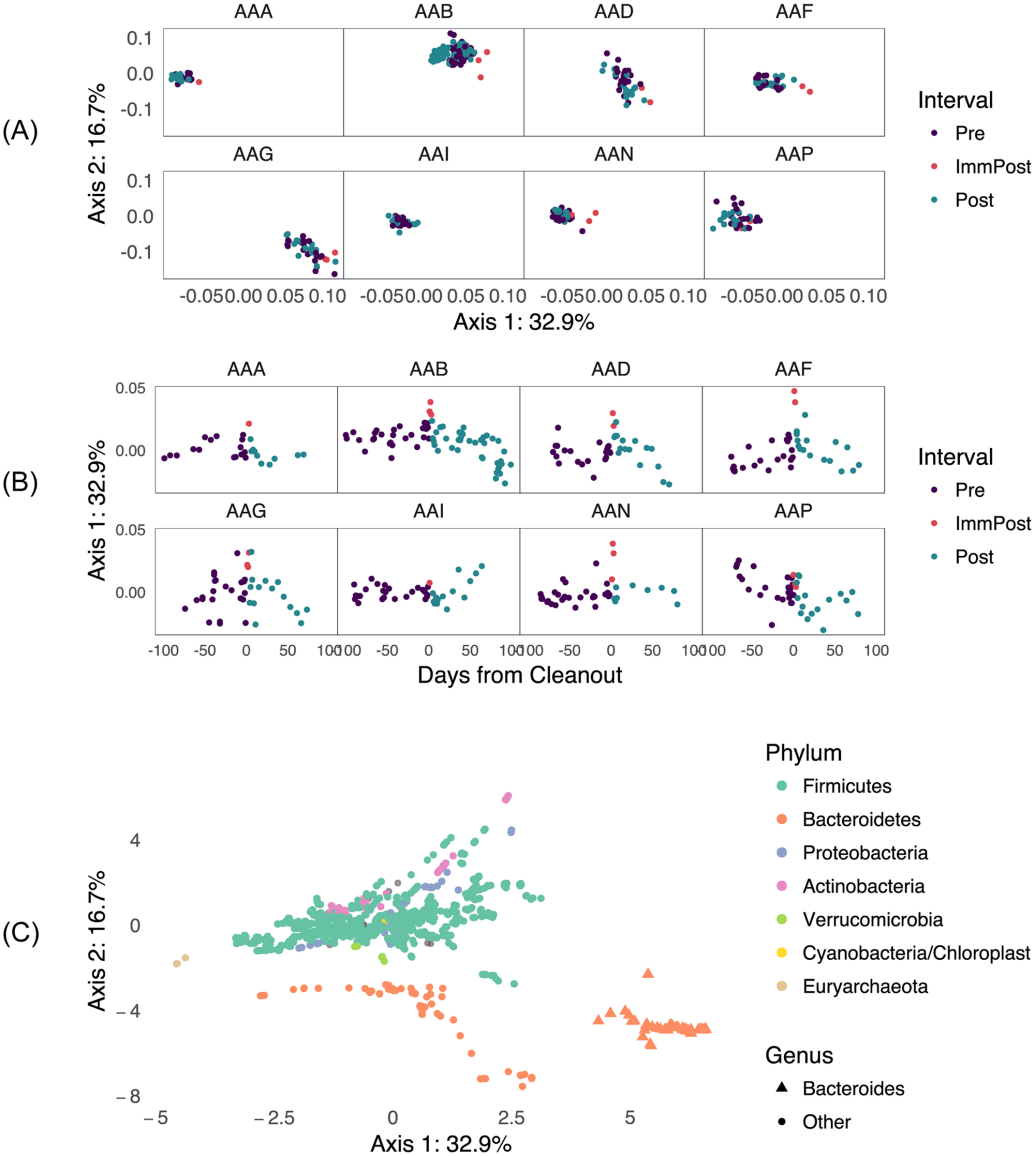
Several views of results from adaptive gPCA reveal a brief but definitive IIOD effect. (A) shows the sample scores from agPCA plotted on the first two axes. In (B), the sample scores have been centered by subject so as to better show the within-subject variation, and the centered scores along the first axis are displayed over time. In (C) we show the RSV scores on the principal axes. Compositional inferences in the agPCA method can be made in comparison to the taxon component of the biplot; the main compositional gradient is from abundant Firmicutes (subject AAA, center left) to abundant Bacteroidetes (subject AAG, lower right), while AAB is unique in having high relative abundance of Actinobacteria.

Since we were interested in understanding the major portions of the between-sample variability which could be explained in terms of phylogenetically related groups of RSVs, we performed a phylogenetically-informed ordination of the RSV data using adaptive gPCA. The results of this ordination are shown in Figs 2B and 3A. We still see a subject effect: different subjects are localized to different regions of the principal plane and within-subject distances are generally greater than between-subject differences. However, we now also see an effect of the cleanout in that the samples immediately after the cleanout generally have more positive loadings along the first adaptive gPCA axis than other samples from the same subject. This is shown in more detail in Fig 3B, where we have plotted the scores of each sample along the first axis across time. The magnitude of the effect varies by individual, but points immediately after the cleanout tend to have the most extreme values along the first axis of any of the samples in the corresponding subject.

Finally, we show the RSV loadings along the principal axes in Fig 3C. Since positive scores along the first axis seem to be associated with the samples immediately after the cleanout, we are particularly interested in RSVs which have strong loadings on this first axis. By examining Fig 3C, we see that a subset of the Bacteroidetes phylum has a strong positive loading on the first axis, and is therefore positively associated with the cleanout. This group corresponds exactly to the *Bacteroides* genus (see S1 Fig). Since this genus seems to be associated with the cleanout, it is analyzed further below.

#### Covariation between RSV and metagenomic measurements

**Fig 4:**
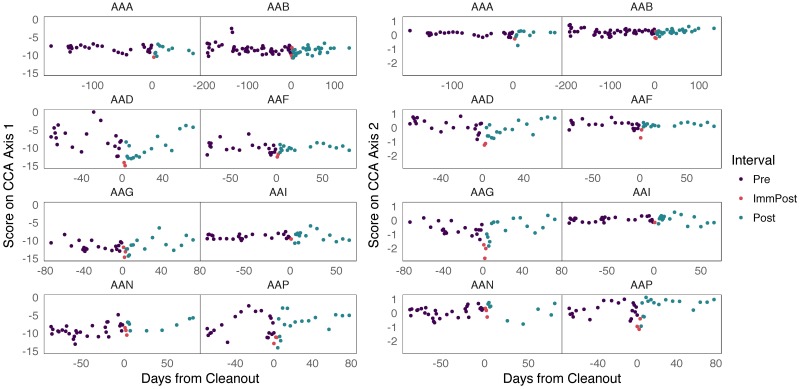
The top two sCCA directions with respect to the bacterial abundance table are generally stable over time, though several subjects show a decrease near the cleanout date. Further, the scale of the scores continues to discriminate between subjects.

**Fig 5:**
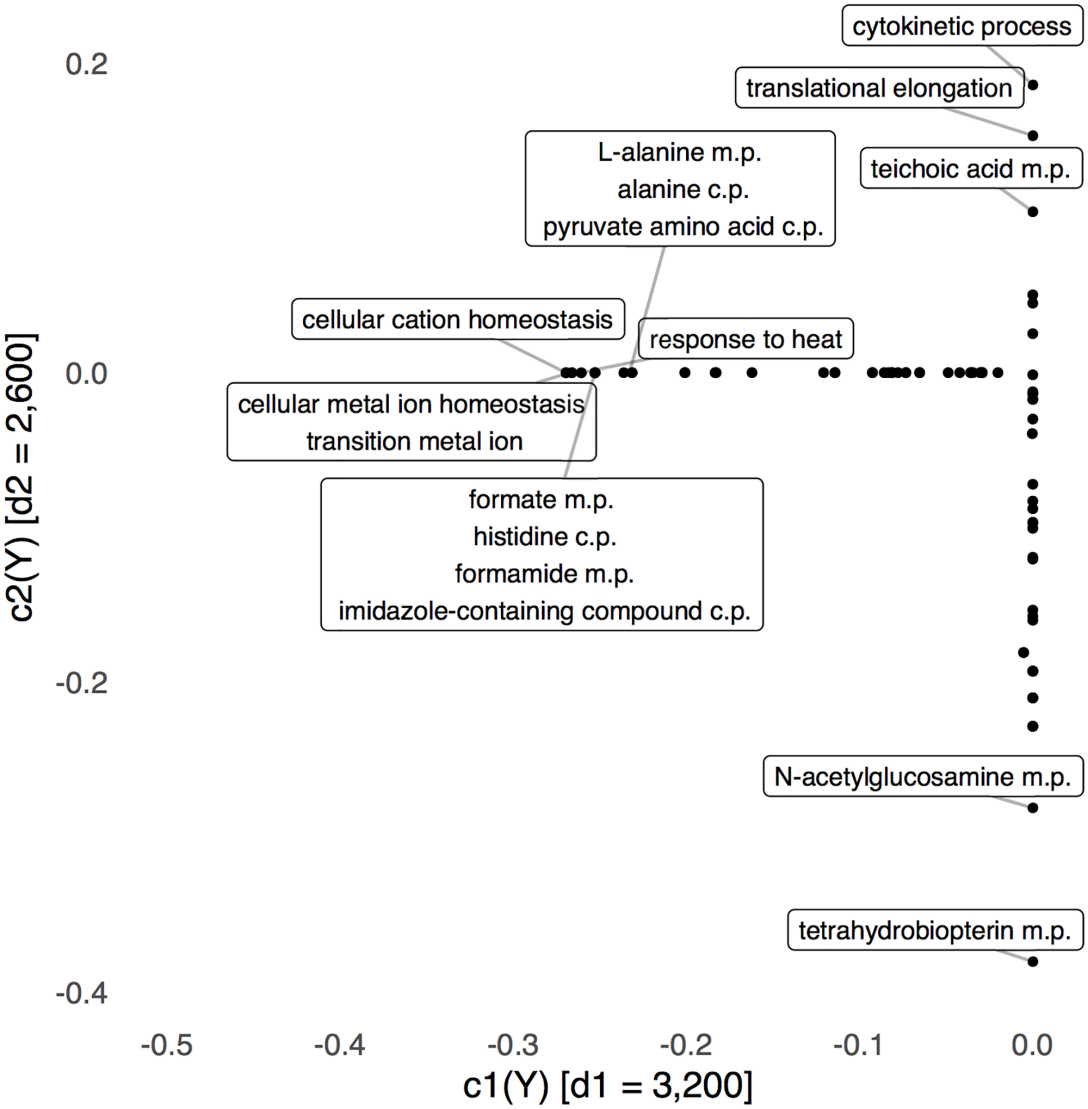
No GO terms are nonzero in both sCCA metagenomic directions. We have only labeled those whose loadings on one of the two axes are large. We abbreviate metabolic and catabolic processes as m.p. and c.p., respectively.

Previous studies have successfully used regularized canonical correlation analyses to connect metagenomic data to metabolic pathways [65]. In our study we have also chosen to characterize the covariation between microbial and metagenomic measurements using a similar approach. We identify measurements that contribute most to this covariation using sparse CCA (sCCA), with results displayed in Figs 4 and 5, S4 Fig. This analysis ensures different data sources are not studied in isolation from one another.

We first describe preprocessing of the data and comment on the sCCA results, then we evaluate the associated biological significance.

As preprocessing, we filtered and transformed both the bacterial abundance and metagenomic data. This focuses sCCA on more substantial sources of variation and ensures that the input distributions are not too skewed. For the bacterial abundance data, we retained only RSVs assigned to the *Bacteroides* genus because these RSVs were the most strongly associated with a cleanout effect. Because genomic count data tend to be heavy tailed, we log(1 + *x*) and asinh transformed the bacterial abundance and metagenomic data respectively to further reduce skewness.

The RSV scores associated with the top two directions are given in Fig 4. The peaks around the cleanout, clearly present in the adaptive gPCA, appear as small drops in both axes of the sCCA, though the effect seems attenuated. The main sources of covariation do not seem as strongly related to the cleanout date, so the metagenomic data likely only exhibit a weak relationship with the latent phenomena that are the main sources of variation in the bacterial abundance data.

**Fig 6:**
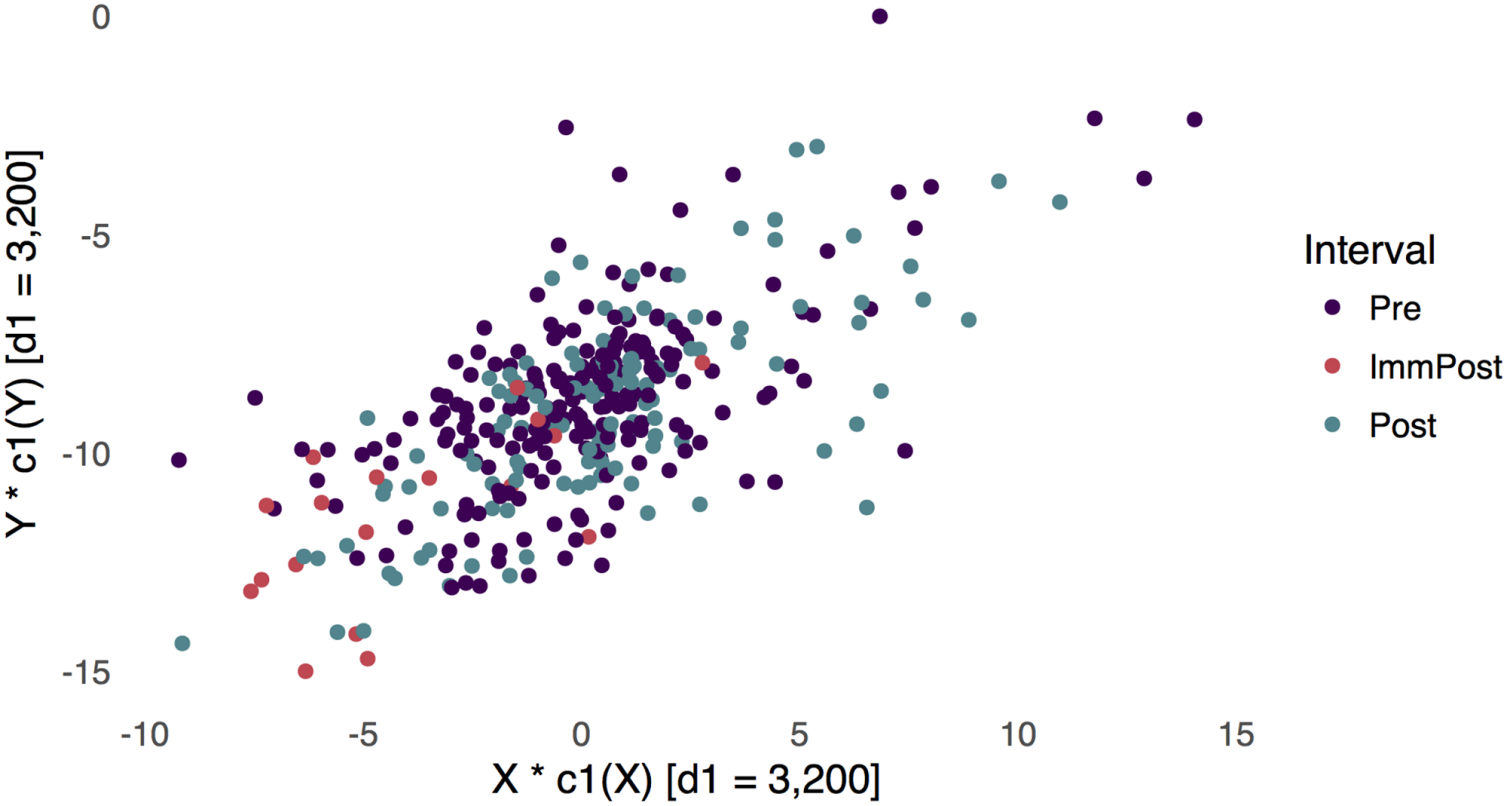
The top scores for the two tables are plotted against each other here, and they have a correlation of 0.663, reflecting the sCCA objective for the fixed regularization parameters.

In Fig 6, we compare the top scores across the two tables. The sCCA objective attempts to maximize the correlation in this display. There is reasonably high correlation between these scores, suggesting that the two tables do reflect some shared latent phenomena, at a global level. In Fig 5 and supporting information S3 Fig, we study the top sCCA directions associated with the bacterial abundance and metagenomic tables, respectively. For the metagenomic directions, note that no terms are nonzero in both coordinates—the sparsity penalty here is relatively aggressive. We have labeled those directions that lie far from zero; these tend to be related to different metabolic and catabolic processes.

#### Sparse LDA identifies clades associated with IIOD

**Fig 7:**
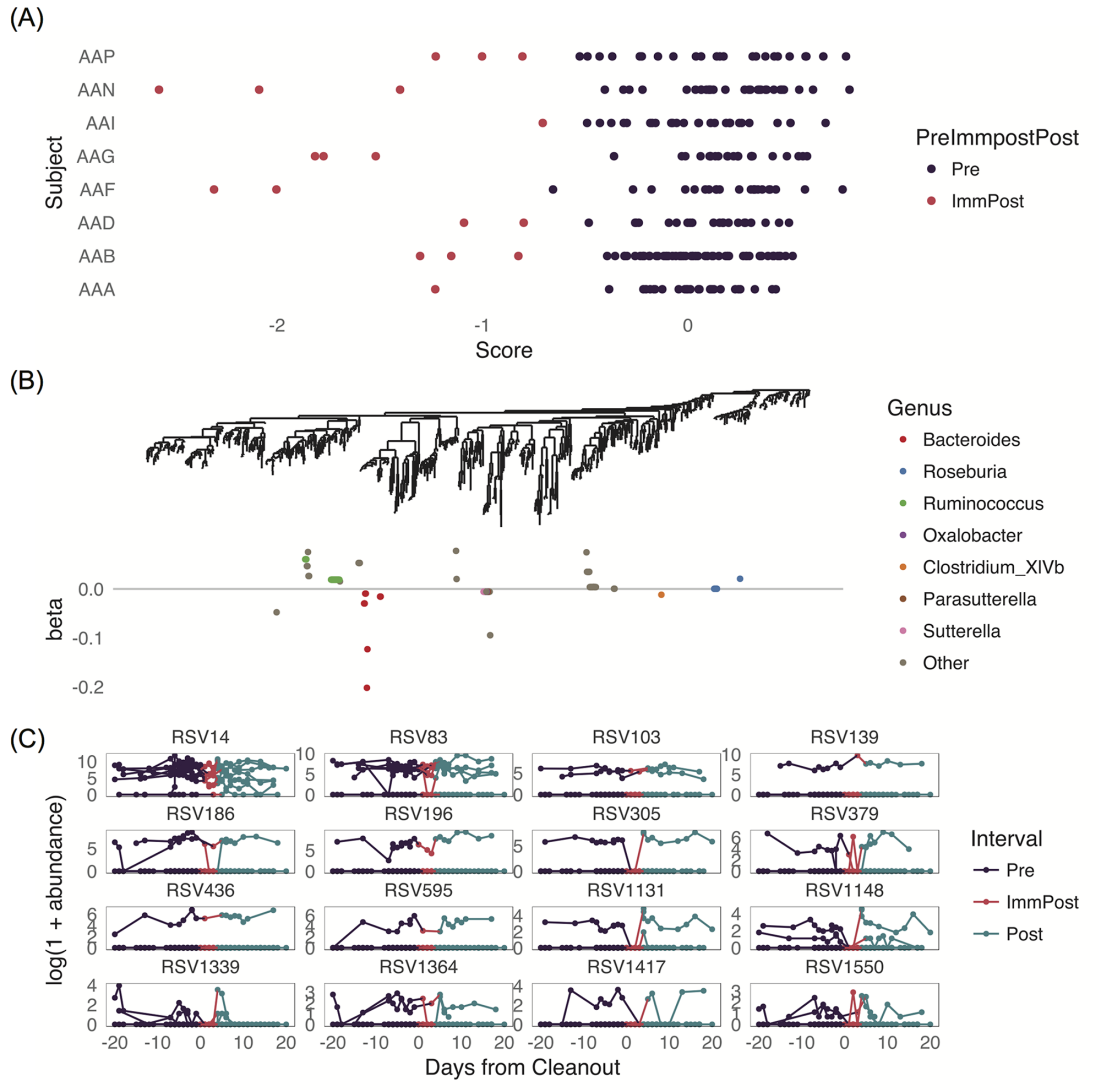
Results from tree-based sparse discriminant analysis. In (A), we see the sample scores on the discriminating axis. (B) shows the RSV loadings on the discriminating axis colored by genus and plotted along the phylogenetic tree. In (C), we show the trajectories of the RSVs in the largest discriminating clade (corresponding to a group of *Ruminococcus* RSVs) over time for each subject. For the taxonomic information for each of these numbered RSVs see the mapping in supporting information S1 Table.

Although we saw a distinct compositional change in the samples in the period immediately after the cleanout from the unsupervised analysis, we were also interested in whether a supervised approach would give us additional insight into RSVs which separate the samples in this period from the others. Both because we expected similar RSVs to respond in a similar way to IIOD and because groups of phylogenetically related RSVs are more interpretable biologically than lists of unrelated RSVs, we used the tree-based version of sparse LDA described above to discriminate between pre-cleanout samples and the samples in the period immediately after the cleanout (the method is implemented in the treeDA R package see [66]). We filtered the RSVs to those which were present at an abundance of at least 5 in at least 10 of the samples, which led to a set of 1207 RSVs. We used log-transformed RSV abundances and used the tree-based sparse LDA to discriminate between the pre-cleanout samples and the samples taken in the three days after the cleanout. The optimal number of predictors was determined by cross-validation to be 25, which corresponds to 80 RSVs with non-zero coefficients since many of the predictors corresponded to nodes in the tree. A plot of the sample scores along the discriminating axis is shown in Fig 7A, and we see that this set of predictors clearly separates the two classes quite well, and in particular more strongly than with the unsupervised analysis. Fig 7B plots RSV coefficients against the tree, revealing a mixture of small clades and singleton RSVs which were chosen by the method as discriminatory.

To gain insight into the method and the selected RSVs, we examined the largest clade selected by sparse LDA. This is a group of 16 RSVs, all in the genus *Ruminococcus*. The filtered dataset had a total of 27 RSVs assigned to the *Ruminococcus* genus, and 3 of the other *Ruminococcus* RSVs were selected separately by sparse LDA, suggesting that this genus is substantially associated with the cleanout. The log-transformed abundances of the 16 *Ruminococcus* RSVs selected by sparse LDA are plotted in Fig 7C. In this figure, each line represents one subject, and each facet represents one RSV. From the plots, we see that although the signal is not very strong, there is a tendency for the RSVs in this group to decline in the first few days after the cleanout and then to return to a high level. We also see that most RSVs are present in only one or two of the subjects, and it is not the same subject for each RSV. This is one reason why phylogenetic methods are important for microbiome studies: there are substantial individual differences in the RSVs present in each subject, but it is still possible to learn about groups of related RSVs which show the same behavior conditional on them being present in a given subject.

To ensure that our results were not overly sensitive to the choice of transformation, we repeated the procedure with asinh-transformed RSV abundances. This led to a slightly more parsimonious model, 13 predictors corresponding to 61 leaves on the tree chosen by cross-validation. However, the results were qualitatively the same: the largest clade discovered was composed of RSVs from the *Ruminococcus* genus, and a low abundance of these RSVs was predictive of the immediate post period. A group of RSVs from the *Bacteroides* genus had coefficients of the opposite sign, meaning that large abundances of these RSVs were again predictive of the immediate post period. (See S7 Fig and the code section S1 Data for more details.)

In the LEfSe test (whose results are shown in S9 Fig), only a few of the *Ruminococcus* RSVs occur as loosely significant because many were filtered out before the testing procedure. A group of RSVs from the *Bacteroides* genus also have coefficients of the opposite sign in this LEfSe analysis. Thus the sparse LDA increases the power of detection of differences to similar levels as LEfSe.

### Diagnostics through resilience prediction

Even without discovering the underlying mechanisms of resilience, the development of predictive diagnostics of resilience can be clinically relevant. With only 8 subjects, it is impossible to make any definitive conclusions; however, it is not unreasonable to explore methodological frameworks and propose possibly predictive factors.

One approach to this problem is to define a scalar measure of resilience within each subject, and then attempt to predict this resilience measure using information known before any perturbation is performed. Any pre-perturbation features that may be predictive of resilience could become potential diagnostics.

To characterize resilience, we use the relative change in Shannon diversity, computed over windows immediately preceding and following the cleanout. We use a window of length 3 days. As potential predictors of community recovery following severe perturbation, we consider taxonomic composition at the family level. Additional potential predictors include features from other measurement domains and/or derived features, a more complete supervised model would require more subjects and will be the focus of a complete followup study. While it is not unreasonable that the relative abundance of particular taxa (e.g. nutritional generalists or specialists) might influence community resilience, we are choosing this particular measure primarily to demonstrate the predictive methodology.

Upon applying an elastic net regression to this problem, tuned by bootstrap resampling, we identify three families with nonzero coefficients, displayed in S8 Fig.

There is a hint of an association between early presence of these bacteria and change in diversity after cleanout. For example, it seems that when Streptococacceae or Enterobacteriaceae are present at the onset of sampling, diversity actually *increases* post-cleanout, while when Prevotellaceae is more abundant at onset, diversity decreases. Of course, new data would need to be collected to validate these claims.

### Data and code availability

Sequencing reads from the V4 16S survey and shotgun metagenomic sequencing are available from the NCBI Short Read Archive via BioProject PRJNA388263. The adaptive generalized PCA programs have been combined into an R package called adaptiveGPCA on CRAN (https://cran.r-project.org), the tree-aware sparse discriminant analysis code is available as the R package treeDA available on CRAN.

All code Rmarkdown, R scripts, and data have been combined into the supporting information S1 Data which contains a tar file. There is also a larger docker file (cleanout_submit.tar) available at the Stanford digital repository permanent url: https://purl.stanford.edu/cf264md0197 for those who do not want to install R manually.

## Discussion

By assembling a rich set of taxonomic and metagenomic data from longitudinal sampling and examining these through several statistical lenses, we investigated the effect of IIOD on the gut microbiome. Specifically, we pursued the following study aims: 1) determine whether and how quickly the gut microbiota demonstrates resilience after IIOD perturbation, 2) elucidate patterns of taxonomic and functional change that characterize the community recovery process, and 3) develop statistical methods for the examination of multidomain data that provide greater biological interpretability than existing methods.

### Immediate response to IIOD is a transient community shift followed by recovery of pre-perturbation state

The present study constitutes an investigation of unprecedented rigor—with regard to length of sampling time period, temporal resolution of sampling, and generation of multiple data types—of the effects on the gut microbial community of a disturbance type, intestinal cleanout, relevant to clinical practice and ecological theory.

As controls for comparison to the perturbed samples of each subject, we used unperturbed samples of the same subject, rather than making a comparison between distinct groups of subjects which did or did not experience IIOD. A simulation study (the details and associated figure of which are available in the supporting materials S5 and S6 Figs) confirms that in our context within-subject comparisons have greater power to reveal IIOD effects because inter-individual variation in the composition of the healthy adult human gut microbiota is greater than temporal variation within an individual [67, 68], even across experimental perturbations (see [14, 69, 70]).

As a result, this work resolves questions raised by previous studies of induced diarrhea [20–24]. These previous studies solely examined 16S rRNA taxonomic data and reached conflicting conclusions about effects of the perturbation on fecal microbiota in healthy adults. Some differences in past reported outcomes are likely attributable to variation among studies in clinical procedures and analytical methods. However, these prior studies collected only 2–5 samples per subject, with gaps of one week to one month between a sample representing the perturbed community and the earliest follow-up sample. Without fine-grained sampling beginning prior to perturbation onset and continuing until the community regained stability, these past studies could neither establish the timescale of recovery nor characterize the recovery process. In addition, by collecting samples at daily or weekly intervals for months before and after perturbation, we were able to assess the effect of this disturbance within the context of ordinary temporal variation for each subject. We have found and characterized a definitive but very transient effect of the colon cleanout. That is, we identified consistent changes in microbial community composition across all subjects in the first days following perturbation, after which the communities reverted to their pre-cleanout states. Furthermore, we found that no other phenomenon during the long sampling interval of normal temporal variation preceding IIOD compared in magnitude to the perturbation effect of IIOD on the community.

In both the adaptive gPCA and the sparse CCA, the samples before and after the cleanout (excluding those from the period immediately after the cleanout) occupy the same region on the axes, suggesting there is no long-term compositional change resulting from cleanout. This rapid return to the pre-cleanout state is consistent with clinical observations that colon cleanout prior to colonoscopy for screening purposes in healthy individuals rarely leads to complications.

### Response to IIOD perturbation differs among RSVs: Bacteroides blooms while ruminococcus lags behind

The depth of sampling surrounding perturbation enabled characterization of the community recovery process using both taxonomic and metagenomic data. While community-wide metrics rapidly attain pre-perturbation states, we observe variation in recovery patterns on finer phylogenetic scales. Specifically, members of the *Bacteroides* genus recover quickly and dominate samples taken immediately post-perturbation while *Ruminococcus* genus members are slower to recover.

Examining loadings of the RSVs on the agPCA axes offers insight into details of the compositional changes that accompany the cleanout.

These results are partially consistent with some found in earlier studies. Gorkiewicz *et al*. found elevated relative abundance of OTUs within *Bacteroides* in fecal samples collected on the 3rd day of PEG-induced chronic diarrhea [24]. Drago *et al*. found reduced relative abundance of Firmicutes in fecal samples collected the day after bowel preparation with a combined stimulatory and osmotic laxative [21]. Shobar *et al*. found an elevated Bacteroidetes:Firmicutes ratio in dilute fecal material recovered via endoscopy from healthy subjects within a day of bowel lavage [22].

Several related biological mechanisms may explain the increased relative abundance of the Bacteroidetes phylum and *Bacteroides* genus, and decreased relative abundance of the Firmicutes phylum and *Ruminococcus* genus in the period immediately after the cleanout. We describe four potential mechanisms here: physical partitioning, substrate preference, growth rate, and differential oxygen tolerance.

Physical partitioning could elevate *Bacteroides* abundance and decrease *Ruminococcus* abundance post-cleanout because paired fecal and mucosal biopsy samples from the unprepped colon of healthy humans revealed that members of Bacteroidetes are enriched in the mucosal layer, which would favor their retention during cleanout, while members of the Firmicutes are enriched in feces [71]. Furthermore, Firmicutes, and in particular members of the *Ruminococcus* genus, prefer attachment to undigested food particles over inhabiting the liquid phase of the gut lumen [69]. Attachment to food particles may enhance removal of *Ruminococcus* during cleanout.

Differential use of growth substrates among the phyla may also contribute to elevated *Bacteroides* abundance and decreased *Ruminococcus* abundance post-cleanout. IIOD removes essentially all diet-derived substrates from the colon. Species capable of growth on the host-derived resources that would be available during and immediately after cleanout could begin to repopulate the colon earlier than specialist species that rely on specific diet components. Prominent gut Firmicutes tend to be nutritional specialists, whereas gut Bacteroidetes and members of the *Bacteroides* genus in particular are versatile foragers capable of growth on host-derived mucin [72, 73].

A related but distinct potential explanatory mechanism for the compositional changes seen immediately post-cleanout is variation in intrinsic growth rates. Differential growth rates are related to the generalist/specialist mechanism in that generalists preferentially consume the resources that permit the fastest growth. Competition for labile substrates ensures their rapid depletion and advantages organisms capable of resource switching. On the other hand, nutritional specialists can persist in a flowing environment like the gut only if their preferred resource is reliably available, which requires that the resource not be easily degraded. The lower energy yield and/or slower rate of the catabolic reactions in degradation of a recalcitrant substrate, perhaps coupled with greater investments in requisite enzymes, imply slower maximal growth rates for specialists. In the unperturbed gut, reduced resource competition means that microorganism growth need only keep pace with the flow rate of the gut, so rapid growth is not as important for specialist fitness. The median ribosomal RNA operon copy number per genome is correlated with maximal growth rates of microbes [74, 75]. On this basis, *Bacteroides* (median 6 *rrn* copies/genome) are likely to be capable of faster growth than *Ruminococcus* (median 4 *rrn* copies/genome [76]) Comparisons of microbial growth rates in culture are challenging to interpret because experimental conditions may not reflect the native habitat of the gut, but existing data from such experiments are consistent with the hypothesis that *Bacteroides* are generally capable of faster growth than *Ruminococcus* [77, 78].

A final mechanism that may contribute to the over-representation of *Bacteroides* and underrepresentation of *Ruminococcus* in the post-cleanout period is differential oxygen tolerance. Under normal conditions, oxygen diffusing into the colon is rapidly depleted by facultatively anaerobic and microaerophilic microbes, allowing oxygen-sensitive anaerobes to grow in the colonic lumen [79]. The loss of most microbial biomass during cleanout and more rapid diffusion of oxygen through the less viscous intestinal contents that remain would increase oxygen concentration in the lumen of the colon. David et al. reached the same conclusion after observing a shift in the relative abundance of low-affinity vs. high-affinity cytochrome oxidases in the gut microbiome during early stages of succession following secretory diarrhea due to cholera [80]. According to the published literature summarized in Albenberg et al., the *Bacteroides* genus includes both anaerobic and microaerophilic species, while *Ruminococcus* as well as all other genera in the *Ruminococcaceae* family are anaerobic [79]. The published literature may be biased by the relatively recent recognition of widespread microaerophily; a systematic investigation into the respiratory reductases encoded by 254 complete and partial genomes of human gut microbes found evidence for microaerophily in all 43 *Bacteroides* genomes that were examined, but only 4 of 9 genomes from *Ruminococcus* [81].

### Perturbation-associated GO functional terms include both directly-relevant and genomically-linked terms

In addition to the 16S rRNA analyses employed by previous studies of IIOD, we collected metagenomic data and integrated analysis of the two data types using multitable methods. We applied sparse CCA to the combined 16S and metagenomic data to examine possible functional implications of the perturbation. To this end, we recovered a perturbation-related gradient across samples based on GO terms, indicating that changes in community functional capacity from baseline exist in the perturbed state.

The GO terms included in this gradient may elucidate the survival advantages and disadvantages of organisms that recover quickly or more slowly, respectively, after IIOD. However, caution is necessary in the interpretation of GO terms included in the perturbation-associated gradient for several reasons. The GO terms defining the perturbation-associated gradient include a wide range of generality and specificity (e.g., “cellular metal ion homeostasis” and “tetrahydrobiopterin metabolic process”), and the method cannot establish the directionality of the causal relationship between microbial abundance and functional capabilities. That is, based on our data alone, we cannot say whether the GO terms we identified are functionally relevant to the response to perturbation or simply enriched (or depleted) in the genome of microbes that have a characteristic response to the perturbation due to other functional traits. In fact, we note two types of terms highlighted by the gradient: terms reflecting functions of importance for survival in the post-cleanout environment (e.g., catabolism of alanine and pyruvate family amino acids) and terms carried by organisms systematically affected by the cleanout that are not themselves of direct functional importance for the carrier organisms’ survival (e.g., teichoic acid metabolic processes).

We can nonetheless identify the predominant variation in functional terms, without specifying the underlying mechanism, based on Figs 3 and 5. From the relatively small set of terms found to be more strongly associated with the perturbation, we highlight the presence of teichoic acid metabolic processes, which may indeed be a genomic marker of microbes with a characteristic response rather than a function with direct relvance to post-IIOD recovery. Teichoic acid is a cell wall component of the Gram-positive Firmicutes but not the Gram-negative Bacteroidetes, and appears with the expected positive association with both CCA axes. We have no reason to suggest that the presence or absence of teichoic acid *per se* influences microbial survival during the cleanout, but it is reassuring that a functional term known to be correlated with those taxa that are differentiated by other relevant functional traits is identified by this statistical technique. A functional trait that appears in several distinct clades of bacteria and is also selected by sparse CCA is more likely to reflect a function that is relevant to the perturbation, and such may be the case for the cluster of functional terms related to the catabolism of alanine and pyruvate family amino acids. Protein-coding genes from members of the Bacteroidetes, Firmicutes and Proteobacteria phyla (among others) are annotated with these terms and amino acid fermenting microbes belonging to 6 genera in these 3 phyla are known to associate with the human colonic mucosa [79] where they may resist elimination during the cleanout and would have access to host-derived protein. Furthermore, because proteolytic microbes are typically much less common in the gut relative to saccharolytic microbes, the selection of these functional terms by sparse CCA is less likely to be due to chance association with a broader taxonomic group.

### Statistical regularization improves interpretability and facilitates multidomain analysis

While analyzing these data, we developed new methods and applied existing methods in novel ways. Some of the key issues were: high dimensionality, the simultaneous study of multiple data sources, and our desire to have biologically interpretable results. The issues of interpretability and high dimensionality were both addressed with statistical regularization, either through the use of a sparsity constraint, through incorporation of the phylogenetic structure or using both approaches. The simultaneous study of multiple data sources was performed in an interpretable way using sCCA (sparse CCA).

Adaptive gPCA and tree-based discriminant analysis offer more flexible and interpretable incorporation of information regarding phylogenetic relatedness among observed RSVs than existing methods. In both cases, the aim was to obtain an explanation of the variation between the samples in terms of groups of closely-related RSVs. We expected this constraint to be useful both because groups of closely-related RSVs are more biologically interpretable than lists of unrelated RSVs and because we expect closely-related RSVs to respond in similar ways to IIOD. In adaptive gPCA, we are interested in explaining the overall variability between the samples in these terms. Currently, the most common approaches for comparing samples in microbiota studies either ignore the phylogeny entirely (e.g. Bray-Curtis) or incorporate the phylogeny in a fixed way (e.g. weighted Unifrac). Furthermore, the ordination axes resulting from these approaches are not directly interpretable in terms of which microbial taxa are most important for positioning samples in the lower dimensional space. In contrast, adaptive gPCA allows more fine-tuned control of the extent to which phylogeny is reflected in the analysis and offers explanations of the ordination axes in terms of closely related RSVs.

In tree-based discriminant analysis, we were interested in explaining the difference between the samples at baseline and the samples immediately after IIOD, but we again wanted the explanation to be in terms of groups of phylogenetically-related RSVs. By including features associated with internal tree nodes, the tree-based discriminant analysis allows identification of larger evolutionary units whose members are all associated with the response. Without this enrichment in the feature space, it is only possible to read off individual RSVs associated with the response and then attempt to assess phylogenetic relatedness in follow-up analysis. Further, in a limited sample-size setting, individual microbe effects may be undetectable, while aggregate evolutionary-unit level signals may be clear. In this situation, only a model incorporating these higher level units as features would succeed. The analysis also incorporates a sparsity constraint, meaning that in the final model most of the RSVs are considered unimportant in explaining the differences between the groups and giving us just a small number of related RSVs to focus our attention on.

sCCA described here offers improved integration of analysis on multiple datatypes collected from the sample set. Most microbiota studies have employed only a single analytical technique (most often 16S rRNA gene surveys), although an increasing number of studies apply additional techniques (e.g. metagenomics, metabolomics) to at least a subset of samples. However, the data derived from each technique has typically been studied in isolation, not exploiting the fact that various techniques have been applied to the same set of samples. By explicitly seeking aspects of the data structure that are shared across multiple data types, multitable statistical analyses can provide insight into the fundamental biological processes responsible for the patterns observed via different techniques. For example, sCCA defines ordinations based on the latent factors present across all data sources, down-weighting the influence of factors present in isolated data types. Consequently, the positions of samples in the reduced space is informed by relatedness across multiple data types. Further, the factors recovered by sCCA can illuminate sets of features across multiple data types that are correlated with one another, suggesting the presence of fundamental biological processes driving parallel changes across data types.

Both sparse LDA and sCCA induce sparsity through *ℓ*^1^ regularization, reducing variance and improving interpretability in the high-dimensional regime. The high dimensionality of modern ‘omics’ data poses a problem for traditional statistics because the hundreds or thousands of identified features (e.g., microbial taxa, functional genes) generally greatly exceeds the number of samples analyzed. The problem of identifying meaningful associations in high-dimensional data is often handled with a FDR approach, which seeks to provide the largest possible list of features that vary in the comparison, while keeping the rate of false positive feature identifications below a certain threshold. The resultant long lists of features can be difficult to interpret, especially when separate lists of significantly varying features are generated from different analytical techniques. An alternative approach is to apply a sparsity constraint during feature selection, which seeks to restrict the list of significant features to the small set most strongly associated with the comparison of interest. In contrast to testing, sparse models can encode specific structure—for example, phylogenetic or multidomain structure—while still providing a parsimonious description of the essential signals in a data set. Dense LDA coefficients or CCA factors can be difficult to inspect, relative to sparse versions which allow attention to be focused on the subset of coordinates with nonzero values. Further, without some form of regularization, ordinary LDA and CCA are statistically unidentifiable in the case that the number of features exceeds the number of samples, as in the IIOD experiment. Even in the case that the number of features is slightly smaller than the number of samples, unregularized models can be alarmingly unstable. Across analysis types, sparse models can encode known structure, simplify inspection of coefficients, and improve model stability.

### Implications and limitations of the study results

The recovery process post-IIOD observed here has potential implications for clinical practice. While colonoscopy (and *a fortiori* the IIOD used to prepare the bowel for the procedure) has a low rate of complications for healthy adults undergoing colonoscopy for colorectal cancer screening, for ulcerative colitis patients colonoscopy is associated with an exacerbation of symptoms [82]. Both the reduced abundance of *Ruminococcus* that are prominent producers of anti-inflammatory butyrate in the human gut [72] and the potential for increased abundance of pro-inflammatory facultative anaerobes of the Proteobacteria phylum [83] could contribute to this phenomenon. Prebiotic interventions to increase the relative abundance of butyrate-producing microbes before and after the colonoscopy [84] (given the depletion of such organisms in IBD [85]), as well as irrigation of the colon with sodium butyrate solutions at the time of colonoscopy [86] may help reduce post-colonoscopy symptoms and hasten the return to a balanced microbiota in IBD patients.

One way forward in illuminating the microbial and functional landscape related to perturbations and temporal variability would be to augment metagenomic data with metabolomic or transcriptomic measurements, applying the statistical techniques described here. These methods provide data that could be used to interrogate microbial function and activity with less potential for confounding due to the covariation of relevant functional traits with other genes carried on the same bacterial genomes. For example, it would be possible to directly quantify short chain fatty acids or secondary bile acids using metabolomic techniques and relate these measurements to the expression of recognizable genes from both characterized and uncharacterized microbial taxa. The provisioning of these and many other compounds are recognized as ecosystem services of the gut microbiota, with health effects both locally in the gut and systemically throughout the host [87, 88].

The new tools and insight described in this work provide guidance and a framework for a more comprehensive assessments of stability and resilience in complex ecosystems, such as the human microbiome. The use of longitudinal study design and multidomain analysis, as we and others are now undertaking, will reveal ecosystem features that are both predictive and diagnostic of key health-associated attributes, and will guide new forms of informed intervention.

## Supporting information

S1 Data Reproducible research through R markdown and data files. This archive contains the R code, data and html output with figures as generated by the code in png and eps formats. All code and data for reproducing the analysis and figures in this study are also available on the Stanford Digital Repository purl https://purl.stanford.edu/cf264md0197. A docker image containing code and data, with all required packages preinstalled, is available at the SDR purl https://purl.stanford.edu/cf264md0197.
(ZIP)

S1 Fig Stacked bars display showing community composition within subjects, in the days surrounding the perturbation. Each row corresponds to a subject, and the *x*-axis provides the day number, relative to the perturbation. For a single *x*-value, bars are colored according to the taxonomic composition of that sample, at the family level.
(TIF)

S2 Fig Stacked bars for weeks around perturbation. Each row corresponds to a subject, and the *x*-axis provides the week number, relative to the perturbation. For a single *x*-value, bars are colored according to the taxonomic composition of that sample, at the family level.
(TIF)

S3 Fig MDS using Bray-Curtis distances. This plot shows that simple MDS on Bray-Curtis distances fails to convincingly separate immediately post-cleanout samples from the rest.
(TIF)

S4 Fig sCCA factors for a subset of taxa. Only taxa members of the *Bacteroides* genus were used in this sCCA analyis. These taxa were identified by agPCA to be relatively more abundant in the period after the cleanout. The numeric labels represent indices of RSVs, they serve as shorthand for full sequence identity, the corresponding taxonomic information is available in S1 Table.
(TIF)

S5 FigPower study simulation.Simulated data at a subset of parameter settings provide a comparison of the crossover longitudinal design with a parallel design. This snapshot shows some of the data from the simulation experiment. From left to right across columns, the true effect size is increased, while from top to bottom, intersubject variability is increased. In the Experimental design subsection of the Methods section we discussed the motivation behind dividing each subject into treatment and control timepoints, rather than allocating separate study subjects as controls, who would never receive any IIOD. To quantitatively characterize the impact of this choice, we performed this simulation experiment. We considered two experimental designs. In both, 8 subjects are tracked for 21 days, with 10 days before and after an IIOD day, respectively. For both, we suppose an IIOD effect appears for five days, with the same strength each day, and across all subjects. In the first design, every subject is given an IIOD, while in the second, half are set aside as controls. We call these two designs “internal” and “external”, respectively. We vary two parameters across simulation repetitions—the strength of the treatment effect, and the intersubject variation. More formally, suppose *i* indexes every sample and *s*(*i*) and *t*(*i*) map the sample to its associated subject label and timepoint, respectively. Let *T* be the set of labels of subjects who are given the treatment. Then, we simulate measurements *y*_*i*_ according to
$$\begin{array}{ccc} y_{i}|{\left(\mu_{s}\right)}_{s=1}^{8} & ∼ & N\left(\mu_{s\left(i\right)}+\beta 1\left\{s\left(i\right)\in T\text{ and }t\left(i\right)\in \left[0,5\right]\right\},\sigma^{2}\right)\\ \mu_{s} & ∼ & N\left(0,\tau^{2}\right). \end{array}$$
*τ*^2^ and *β* parameterize the intersubject variability and treatment effect sizes, respectively. In our simulations, we vary *τ*^2^ across 12 values between 0 (no intersubject variation) and 5 (high intersubject variation), and we vary *β* across 30 values between 0 (no treatment effect) and 2 (large effect). Throughout, we set *σ*^2^ = 1. For each parameter combination, we simulate 10 replicates.(TIF)

S6 Fig Simulated comparison of the crossover longitudinal design with a parallel design. We consider two inference approaches, for both experimental designs. These are (1) a mixed effects model with a random effect for subject and fixed treatment effect and (2) an ordinary linear regression that ignores possible intersubject variability. The results displayed in this figure show points that represent one realization of the experiment, with effect sizes on the *x*-axis and *t*-statistics on the *y*-axis. From top-left to bottom-right, the degree of intersubject variation increases. When there is little treatment effect, no method successfully detects it. However, when treatment effects increase, the difference between methods becomes amplified. As expected, when there is no intersubject variation, there is no difference between the mixed and fixed-effects models. Even here, however, it is better to apply treatments to all subjects. After increasing intersubject variability, the performance of the fixed-effects model deteriorates, as its assumptions are no longer met, even approximately. Throughout all intermediate regimes, the model that applies an IIOD treatment to every subject and accounts for intersubject variation is most powerful.
(TIF)

S7 Fig Plots of held-out samples for cross-validation in sLDA. Plots of held-out samples for cross-validation. Cross-validation was performed holding one subject out at a time. To visualize how the model performed on the held-out data, for each fold of the cross-validation we projected the samples from the held out subject onto the discriminating axis fit on the other subjects. The projections of the samples for each subject are plotted above. The separation between the samples in the two groups is not as dramatic as in the model fit with all of the subjects, but for the most part the discriminating axis generalizes to the held out samples, as seen by the fact that for each subject, the samples in the immediate post period tend to have the highest scores.
(TIF)

S8 Fig Resilience prediction using the elasticnet. Here, we display the raw data associated with each nonzero coefficient in the resilience prediction problem. Within each panel, the initial abundance fraction for that family is plotted along the *x*-axis. On the *y*-axis is the model’s response—the relative change in diversity between windows immediately preceding or following the perturbation. The text label is the name of the associated subject. The dashed line corresponds to the situation that diversity does not change at the cleanout.
(TIF)

S9 Fig Results from LEfSe analysis. The analysis was run on the Pre/Post status class variables and the RSV relative abundance matrix. LEfSe uses the Kruskal-Wallis (KW) sum-rank test to find the significantly differentially abundant RSVs in the pre and immediate post cleanout conditions at each taxonomy level. An RSV is retained if at least the Phylum level taxonomy is significantly different in pre and immediate post cleanout conditions. The reduced RSV abundance will be used to compute the linear discriminant analysis coefficients, which separates the pre and immediate post cleanout conditions. The contribution of each RSV to the discriminant axes is given by the corresponding loadings shown in the barplot (A). Red denotes shows RSVs that have an elevated abundance immediately post cleanout, green identifies the RSVs with lower abundances post-cleanout. When applying LEfSe on the relative abundances at the RSV level alone, no RSVs were significant. The sample by sample transformation recommended in this case by the LEfSe implementation increases differential abundance detection power by multiplying the relative abundance by 1,000,000. After this transformation, LEfSe shows elevated Bacteroidetes and a decrease in Firmicutes immediately after the cleanout, consistent with findings from adaptive gPCA and tree-structured sLDA. However, we do not actually recommend this transformation unless all the sampling depths are of order 1,000,000 as this transformation corresponds to artificially inflating the amount of data available and reducing the standard errors (i.e. it is anti-conservative). (B) shows a simplified tree plot of significant RSVs in pre and immediate post cleanout conditions from the LEfSe output using this taxonomy information. In fact, LEfSe also proposes a sequence of tests at different taxonomic levels, however we did not do this here as we believe that it would be preferable to use a multiple testing procedure that incorporates the hierarchy such as that implemented in [64] as illustrated in [42].
(TIF)

S1 Table RSV identifier mapping table. Identifiers for RSV numbers used in text and figures and the taxonomic information for these RSVs. See supporting information S1 Data for the actual R commands that generate this mapping table.
(CSV)
