# Supplementary figures and images for: Multidomain analyses of a longitudinal human microbiome intestinal cleanout perturbation experiment
Source: PLoS Comput Biol. 2017 Aug 18;13(8):e1005706. doi: 10.1371/journal.pcbi.1005706 (PMC5576755; doi:10.1371/journal.pcbi.1005706)

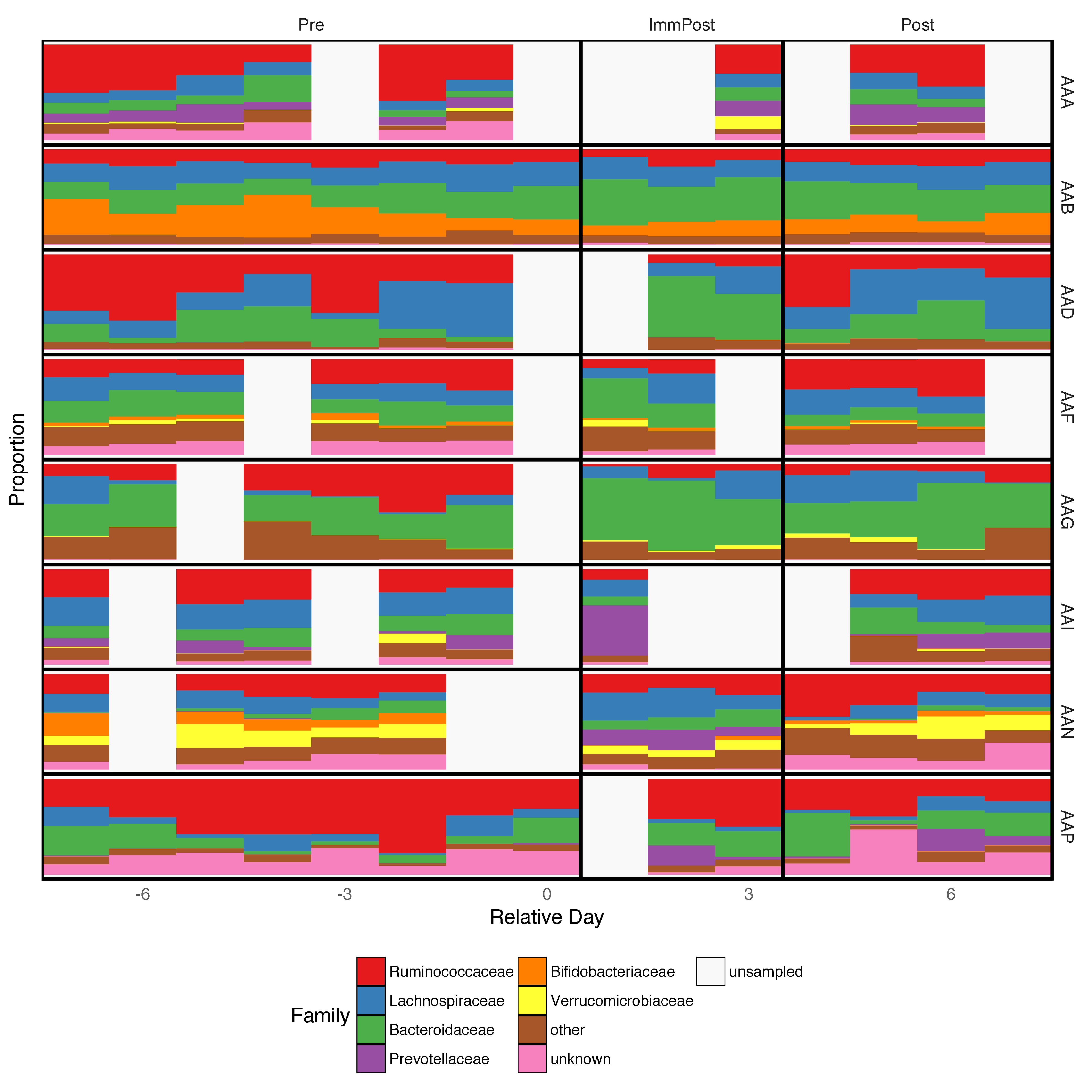

Supplement: S1 Fig — Each row corresponds to a subject, and the x-axis provides the day number, relative to the perturbation. For a single x-value, bars are colored according to the taxonomic composition of that sample, at the family level. (TIF) [file pcbi.1005706.s002.tif]

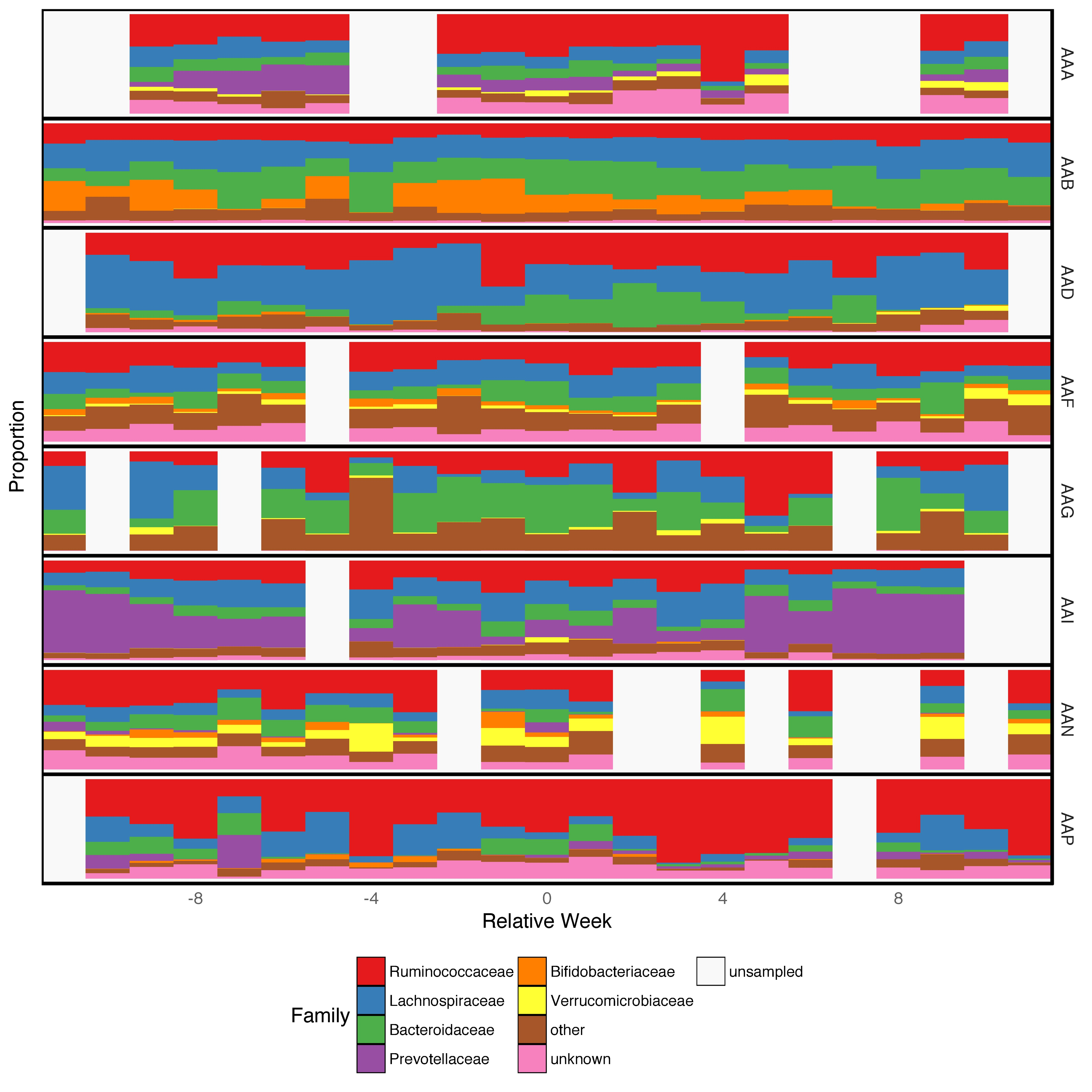

Supplement: S2 Fig — Each row corresponds to a subject, and the x-axis provides the week number, relative to the perturbation. For a single x-value, bars are colored according to the taxonomic composition of that sample, at the family level. (TIF) [file pcbi.1005706.s003.tif]

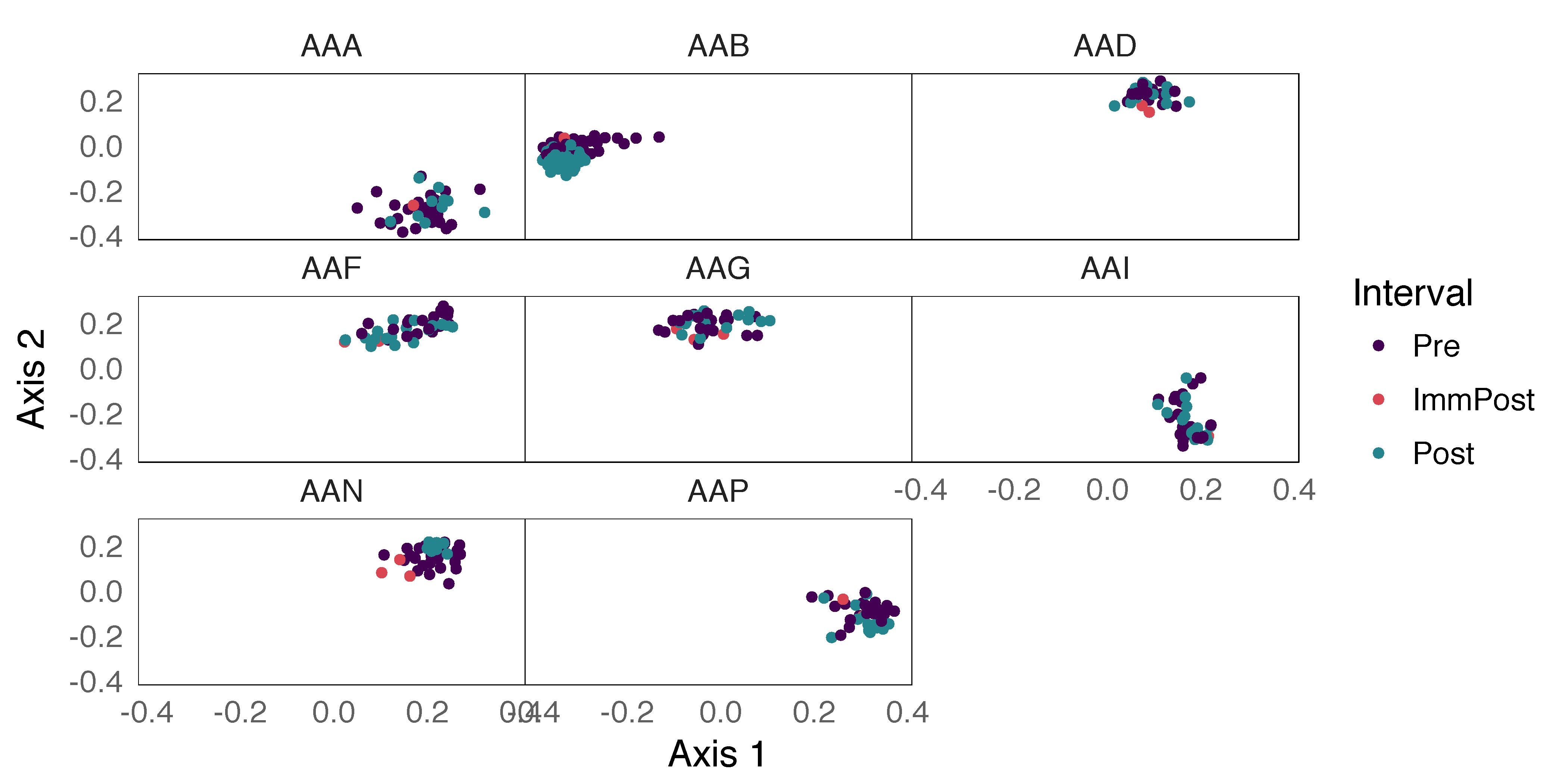

Supplement: S3 Fig — This plot shows that simple MDS on Bray-Curtis distances fails to convincingly separate immediately post-cleanout samples from the rest. (TIF) [file pcbi.1005706.s004.tif]

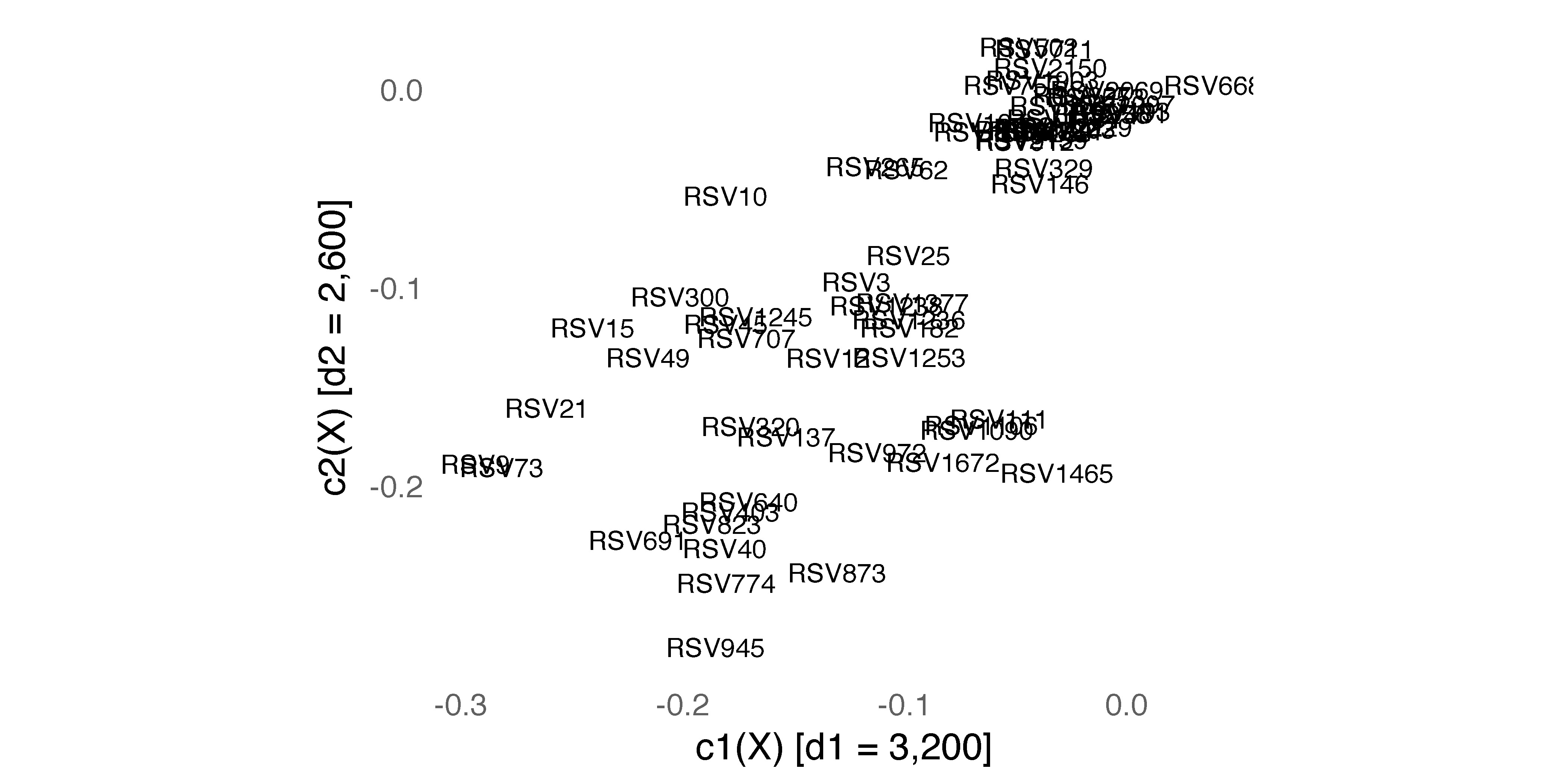

Supplement: S4 Fig — Only taxa members of the Bacteroides genus were used in this sCCA analyis. These taxa were identified by agPCA to be relatively more abundant in the period after the cleanout. The numeric labels represent indices of RSVs, they serve as shorthand for full sequence identity, the corresponding taxonomic information is available in S1 Table. (TIF) [file pcbi.1005706.s005.tif]

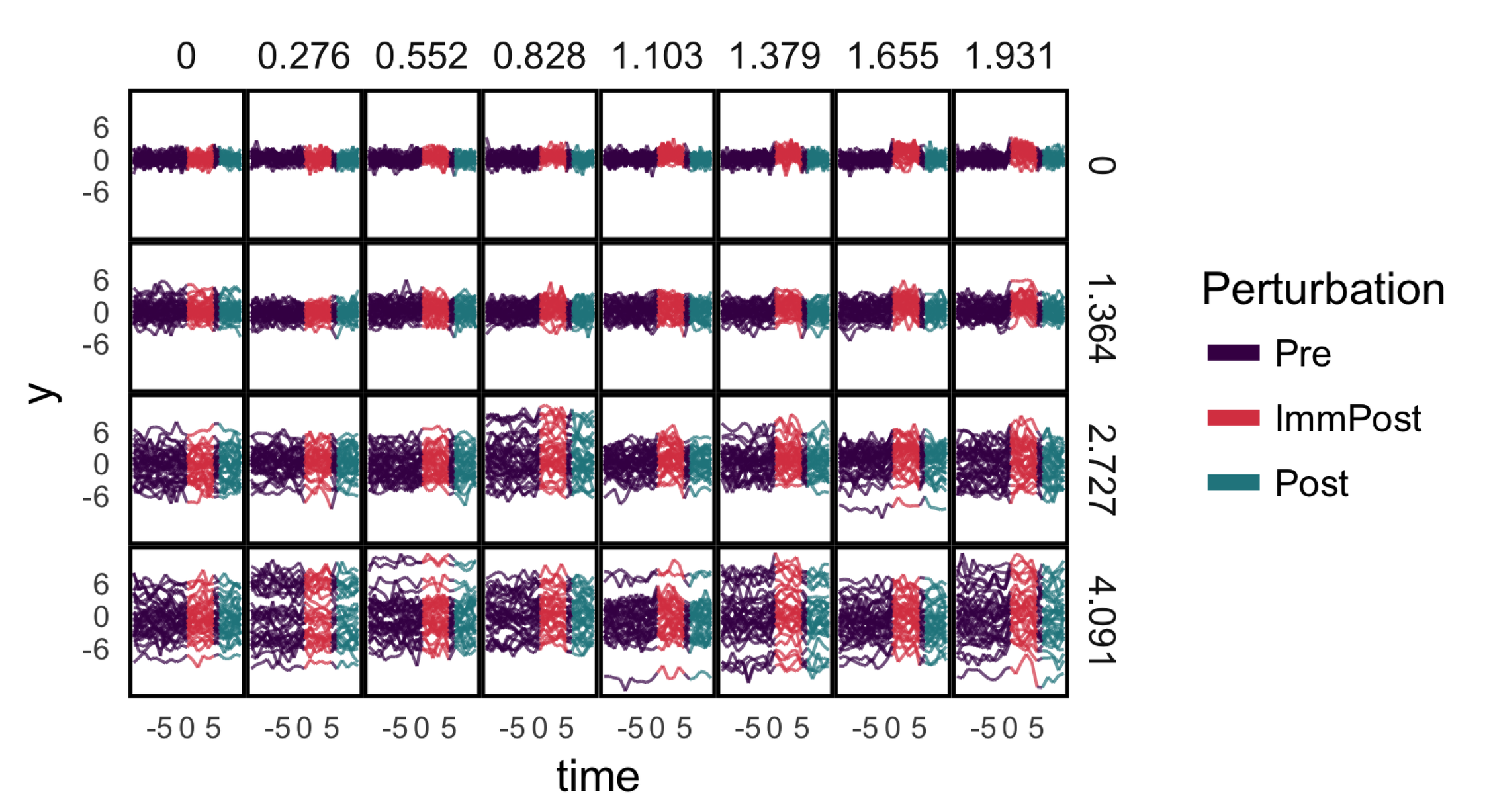

Supplement: S5 Fig — Simulated data at a subset of parameter settings provide a comparison of the crossover longitudinal design with a parallel design. This snapshot shows some of the data from the simulation experiment. From left to right across columns, the true effect size is increased, while from top to bottom, intersubject variability is increased. In the Experimental design subsection of the Methods section we discussed the motivation behind dividing each subject into treatment and control timepoints, rather than allocating separate study subjects as controls, who would never receive any IIOD. To quantitatively characterize the impact of this choice, we performed this simulation experiment. We considered two experimental designs. In both, 8 subjects are tracked for 21 days, with 10 days before and after an IIOD day, respectively. For both, we suppose an IIOD effect appears for five days, with the same strength each day, and across all subjects. In the first design, every subject is given an IIOD, while in the second, half are set aside as controls. We call these two designs “internal” and “external”, respectively. We vary two parameters across simulation repetitions—the strength of the treatment effect, and the intersubject variation. More formally, suppose i indexes every sample and s(i) and t(i) map the sample to its associated subject label and timepoint, respectively. Let T be the set of labels of subjects who are given the treatment. Then, we simulate measurements yi according to yi|(μs)s=18∼N(μs(i)+β1{s(i)∈T and t(i)∈[0,5]},σ2)μs∼N(0,τ2). τ2 and β parameterize the intersubject variability and treatment effect sizes, respectively. In our simulations, we vary τ2 across 12 values between 0 (no intersubject variation) and 5 (high intersubject variation), and we vary β across 30 values between 0 (no treatment effect) and 2 (large effect). Throughout, we set σ2 = 1. For each parameter combination, we simulate 10 replicates. (TIF) [file pcbi.1005706.s006.tif]

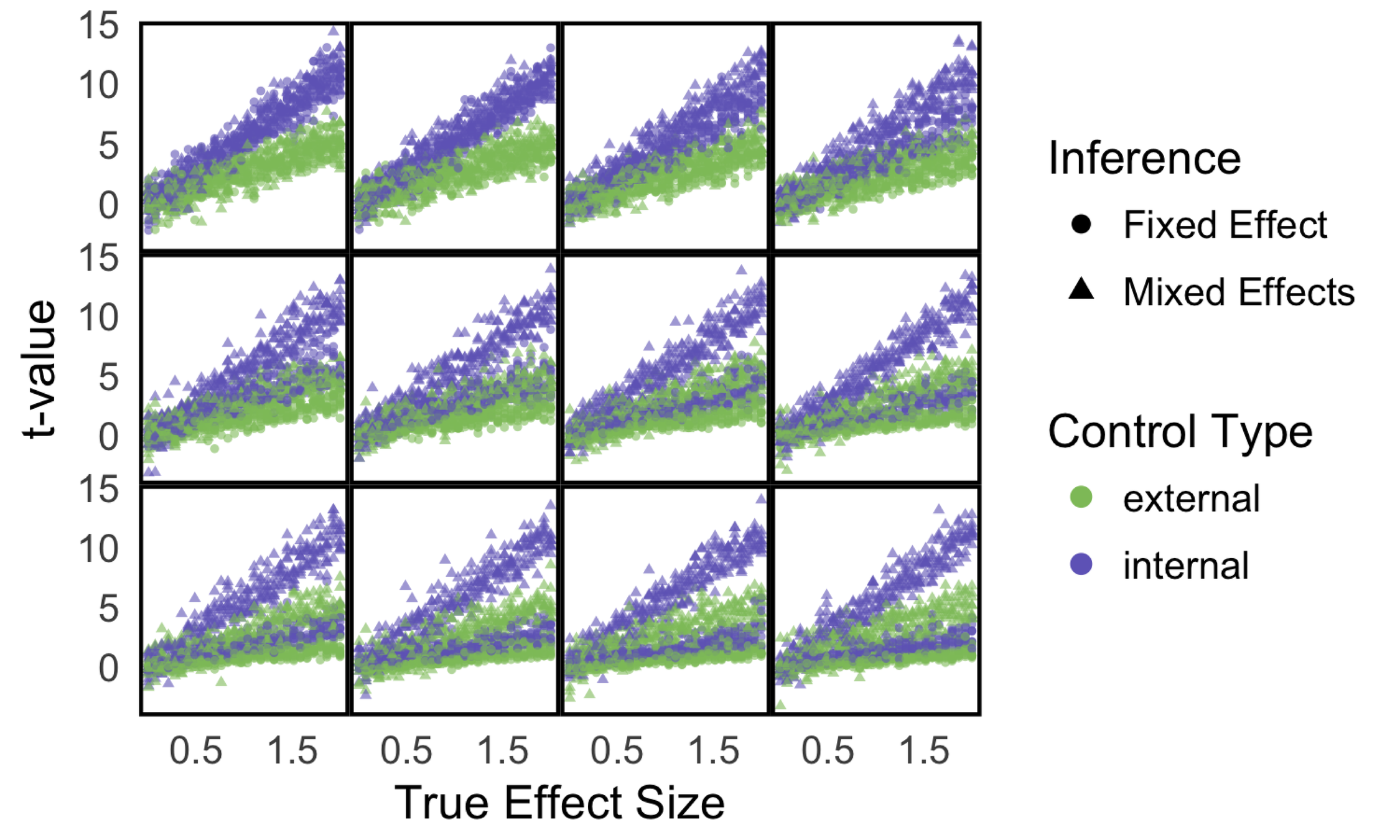

Supplement: S6 Fig — We consider two inference approaches, for both experimental designs. These are (1) a mixed effects model with a random effect for subject and fixed treatment effect and (2) an ordinary linear regression that ignores possible intersubject variability. The results displayed in this figure show points that represent one realization of the experiment, with effect sizes on the x-axis and t-statistics on the y-axis. From top-left to bottom-right, the degree of intersubject variation increases. When there is little treatment effect, no method successfully detects it. However, when treatment effects increase, the difference between methods becomes amplified. As expected, when there is no intersubject variation, there is no difference between the mixed and fixed-effects models. Even here, however, it is better to apply treatments to all subjects. After increasing intersubject variability, the performance of the fixed-effects model deteriorates, as its assumptions are no longer met, even approximately. Throughout all intermediate regimes, the model that applies an IIOD treatment to every subject and accounts for intersubject variation is most powerful. (TIF) [file pcbi.1005706.s007.tif]

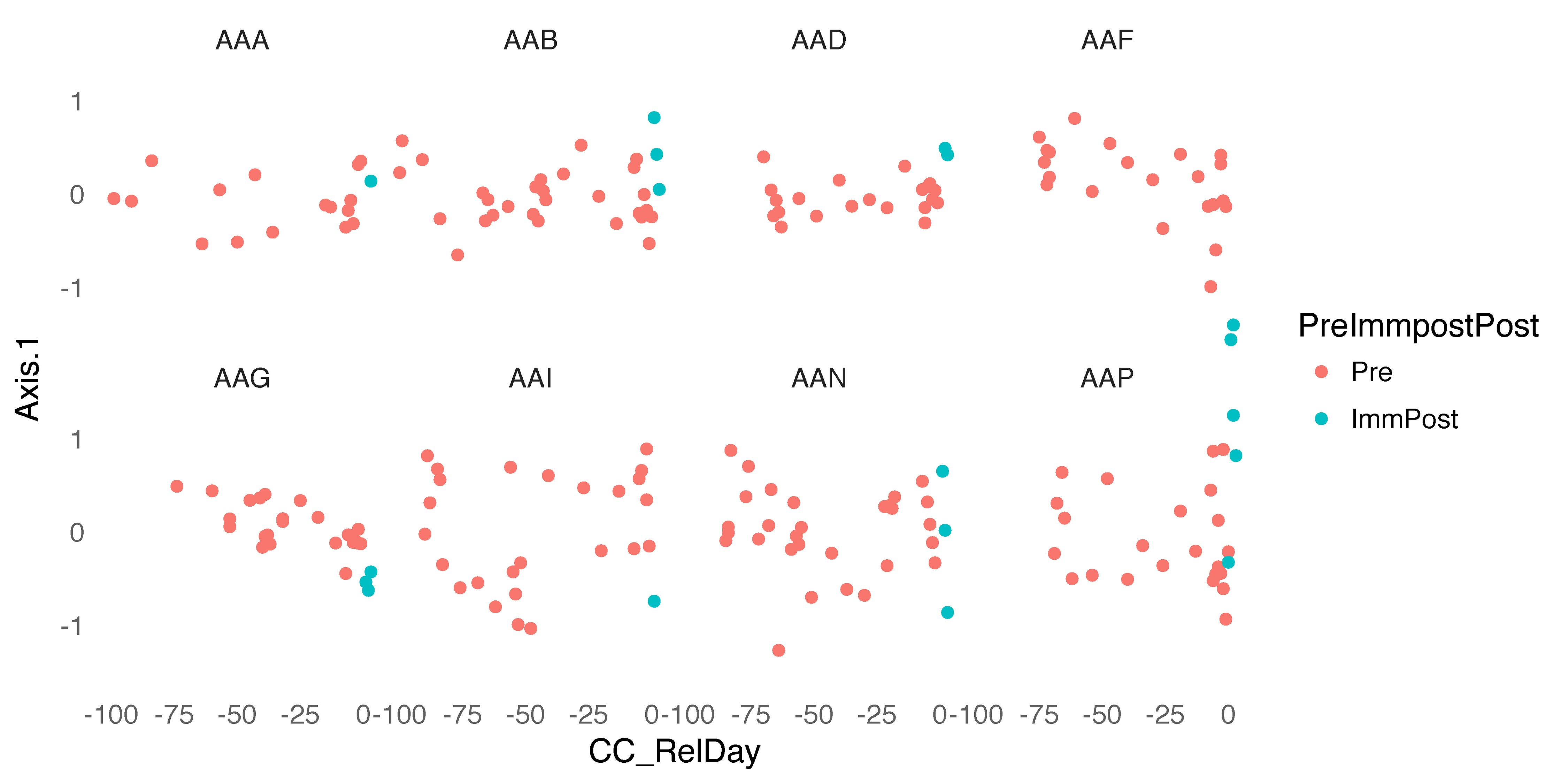

Supplement: S7 Fig — Plots of held-out samples for cross-validation. Cross-validation was performed holding one subject out at a time. To visualize how the model performed on the held-out data, for each fold of the cross-validation we projected the samples from the held out subject onto the discriminating axis fit on the other subjects. The projections of the samples for each subject are plotted above. The separation between the samples in the two groups is not as dramatic as in the model fit with all of the subjects, but for the most part the discriminating axis generalizes to the held out samples, as seen by the fact that for each subject, the samples in the immediate post period tend to have the highest scores. (TIF) [file pcbi.1005706.s008.tif]

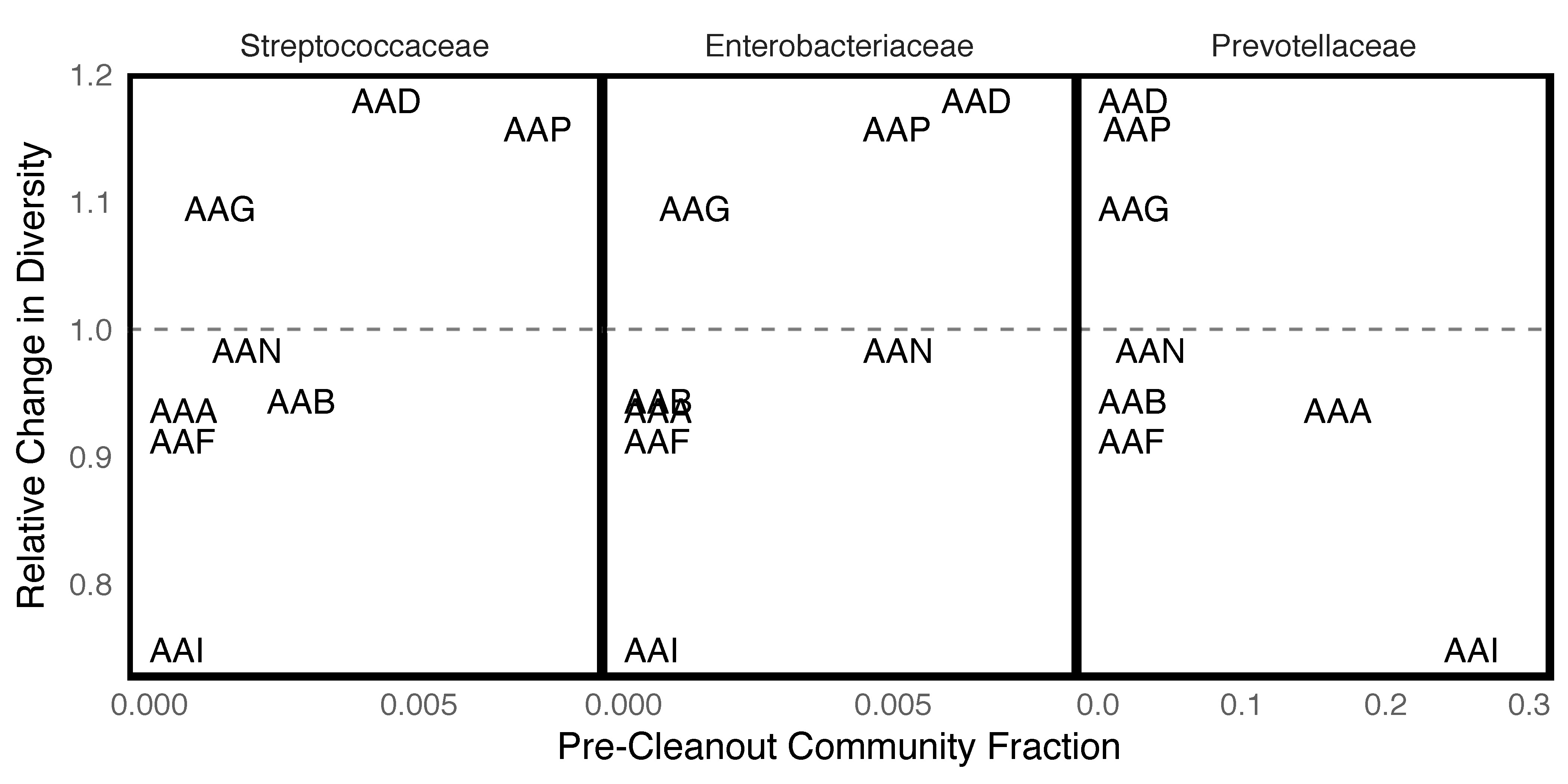

Supplement: S8 Fig — Here, we display the raw data associated with each nonzero coefficient in the resilience prediction problem. Within each panel, the initial abundance fraction for that family is plotted along the x-axis. On the y-axis is the model’s response—the relative change in diversity between windows immediately preceding or following the perturbation. The text label is the name of the associated subject. The dashed line corresponds to the situation that diversity does not change at the cleanout. (TIF) [file pcbi.1005706.s009.tif]

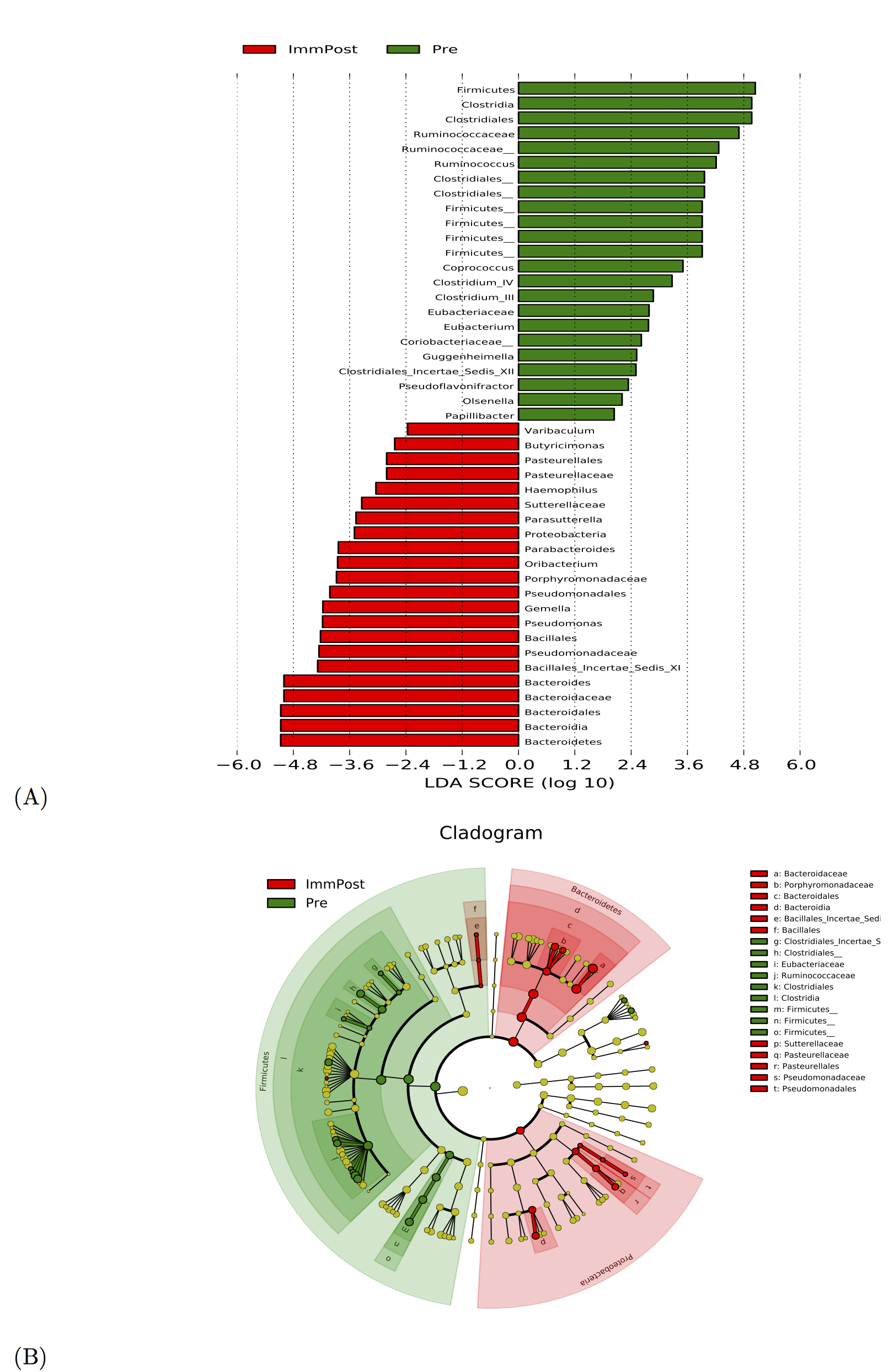

Supplement: S9 Fig — The analysis was run on the Pre/Post status class variables and the RSV relative abundance matrix. LEfSe uses the Kruskal-Wallis (KW) sum-rank test to find the significantly differentially abundant RSVs in the pre and immediate post cleanout conditions at each taxonomy level. An RSV is retained if at least the Phylum level taxonomy is significantly different in pre and immediate post cleanout conditions. The reduced RSV abundance will be used to compute the linear discriminant analysis coefficients, which separates the pre and immediate post cleanout conditions. The contribution of each RSV to the discriminant axes is given by the corresponding loadings shown in the barplot (A). Red denotes shows RSVs that have an elevated abundance immediately post cleanout, green identifies the RSVs with lower abundances post-cleanout. When applying LEfSe on the relative abundances at the RSV level alone, no RSVs were significant. The sample by sample transformation recommended in this case by the LEfSe implementation increases differential abundance detection power by multiplying the relative abundance by 1,000,000. After this transformation, LEfSe shows elevated Bacteroidetes and a decrease in Firmicutes immediately after the cleanout, consistent with findings from adaptive gPCA and tree-structured sLDA. However, we do not actually recommend this transformation unless all the sampling depths are of order 1,000,000 as this transformation corresponds to artificially inflating the amount of data available and reducing the standard errors (i.e. it is anti-conservative). (B) shows a simplified tree plot of significant RSVs in pre and immediate post cleanout conditions from the LEfSe output using this taxonomy information. In fact, LEfSe also proposes a sequence of tests at different taxonomic levels, however we did not do this here as we believe that it would be preferable to use a multiple testing procedure that incorporates the hierarchy such as that implemented in [64] as illustra [file pcbi.1005706.s010.tif]
