# Supplementary material for: Multidomain analyses of a longitudinal human microbiome intestinal cleanout perturbation experiment
Source: PLoS Comput Biol. 2017 Aug 18;13(8):e1005706. doi: 10.1371/journal.pcbi.1005706 (PMC5576755; doi:10.1371/journal.pcbi.1005706)
Supplement: S1 Data — This archive contains the R code, data and html output with figures as generated by the code in png and eps formats. All code and data for reproducing the analysis and figures in this study are also available on the Stanford Digital Repository purl https://purl.stanford.edu/cf264md0197. A docker image containing code and data, with all required packages preinstalled, is available at the SDR purl https://purl.stanford.edu/cf264md0197. (ZIP) [file pcbi.1005706.s001.zip › S0_DataAnalysis.tar!/Rmd_and_data/analysis/analysis.html]

# Figures for PLOS Computational Biology cleanout paper

## Copyright 2017 Julia Fukuyama, Laurie Rumker, Kris Sankaran, Pratheepa Jeganathan,Les Dethfelsen, David Relman and Susan Holmes.

MIT License: Permission is hereby granted, free of charge, to any person obtaining a copy of this software and associated documentation files (the “Software”), to deal in the Software without restriction, including without limitation the rights to use, copy, modify, merge, publish, distribute, sublicense, and/or sell copies of the Software, and to permit persons to whom the Software is furnished to do so, subject to the following conditions:

The above copyright notice and this permission notice shall be included in all copies or substantial portions of the Software.

THE SOFTWARE IS PROVIDED “AS IS”, WITHOUT WARRANTY OF ANY KIND, EXPRESS OR IMPLIED, INCLUDING BUT NOT LIMITED TO THE WARRANTIES OF MERCHANTABILITY, FITNESS FOR A PARTICULAR PURPOSE AND NONINFRINGEMENT. IN NO EVENT SHALL THE AUTHORS OR COPYRIGHT HOLDERS BE LIABLE FOR ANY CLAIM, DAMAGES OR OTHER LIABILITY, WHETHER IN AN ACTION OF CONTRACT, TORT OR OTHERWISE, ARISING FROM, OUT OF OR IN CONNECTION WITH THE SOFTWARE OR THE USE OR OTHER DEALINGS IN THE SOFTWARE.

## Introduction

In this document we show some exploratory analyses of the colon cleanout data. We first notice a large difference in library size which is related to the sequencing run which caused no end of problems when trying to analyze this data processed previously. It doesn’t seem to be as much of an issue this time, but it’s good to keep in mind that this is a potential confounder. We replicate the increase in Bacteroidetes/decrease in Firmicutes that we saw in the OTU data. Finally, we look at some ordination methods and see that some of the phylogenetic methods separate the first couple of days post-cleanout from the remainder of the samples within each subject on the first axis.

The raw sequence data was processed through DADA2. This gave a table with 5497 sequences. The sequences were filtered to only those which were seen at least once in at least 6 samples (out of about 500). The phylogenetic tree was created by first performing multiple alignment of the sequences, construct a neighbor-joining tree as a starting point, and then use this as the input to fit a GTR+G+I maximum likelihood tree (this is the method used in the F1000 paper). The taxonomy table was created using the function in .

## Data setup

The code below is just boilerplate to get our script running. It loads different packages and customizes figure output.

```
library("knitr")
opts_chunk$set(fig.width = 7, fig.height = 3.5, fig.align = "center", dpi = 400,
               dev = c("png", "cairo_ps"),
               fig.show = "asis", size = "scriptsize", cache = TRUE,
               cache.path = "analysis_cache/", warning = FALSE, messages = FALSE)
```

```
# List of packages for session
.packages <- c("data.table", "stringr", "plyr", "dplyr", "tidyr", "ggplot2",
               "viridis", "phyloseq", "ggrepel", "adaptiveGPCA", "Matrix",
               "PMA",  "mvtnorm",  "gridExtra", "RColorBrewer","svglite",
               "scales", "caret", "tibble", "xtable", "devtools")

# Install CRAN packages (if not already installed)
.inst <- .packages %in% installed.packages()
if(any(!.inst)) {
  install.packages(.packages[!.inst], repos = "http://cran.rstudio.com/")
}

if (!("treeDA"  %in% installed.packages())) {
devtools::install_github("jfukuyama/treeDA")
}

Biocpkgs <- c("GO.db","phyloseq","impute")
instBioc <- Biocpkgs %in% installed.packages()
if(any(! instBioc)) {
source("https://bioconductor.org/biocLite.R")
biocLite(Biocpkgs[!instBioc])
}

allneededpackages <- c(.packages, Biocpkgs, "treeDA")

# Load packages into session
sapply(allneededpackages, require, character.only = TRUE)
```

```
## Loading required package: data.table
```

```
## Loading required package: stringr
```

```
## Loading required package: plyr
```

```
## Loading required package: dplyr
```

```
## -------------------------------------------------------------------------
```

```
## data.table + dplyr code now lives in dtplyr.
## Please library(dtplyr)!
```

```
## -------------------------------------------------------------------------
```

```
## 
## Attaching package: 'dplyr'
```

```
## The following objects are masked from 'package:plyr':
## 
##     arrange, count, desc, failwith, id, mutate, rename, summarise,
##     summarize
```

```
## The following objects are masked from 'package:data.table':
## 
##     between, first, last
```

```
## The following objects are masked from 'package:stats':
## 
##     filter, lag
```

```
## The following objects are masked from 'package:base':
## 
##     intersect, setdiff, setequal, union
```

```
## Loading required package: tidyr
```

```
## Loading required package: ggplot2
```

```
## Loading required package: viridis
```

```
## Loading required package: viridisLite
```

```
## Loading required package: phyloseq
```

```
## Loading required package: ggrepel
```

```
## Loading required package: adaptiveGPCA
```

```
## Loading required package: Matrix
```

```
## 
## Attaching package: 'Matrix'
```

```
## The following object is masked from 'package:tidyr':
## 
##     expand
```

```
## Loading required package: PMA
```

```
## Loading required package: impute
```

```
## Loading required package: mvtnorm
```

```
## Loading required package: gridExtra
```

```
## 
## Attaching package: 'gridExtra'
```

```
## The following object is masked from 'package:dplyr':
## 
##     combine
```

```
## Loading required package: RColorBrewer
```

```
## Loading required package: svglite
```

```
## Loading required package: scales
```

```
## Loading required package: caret
```

```
## Loading required package: lattice
```

```
## Loading required package: tibble
```

```
## Loading required package: xtable
```

```
## Loading required package: devtools
```

```
## Loading required package: GO.db
```

```
## Loading required package: AnnotationDbi
```

```
## Loading required package: stats4
```

```
## Loading required package: BiocGenerics
```

```
## Loading required package: parallel
```

```
## 
## Attaching package: 'BiocGenerics'
```

```
## The following objects are masked from 'package:parallel':
## 
##     clusterApply, clusterApplyLB, clusterCall, clusterEvalQ,
##     clusterExport, clusterMap, parApply, parCapply, parLapply,
##     parLapplyLB, parRapply, parSapply, parSapplyLB
```

```
## The following object is masked from 'package:gridExtra':
## 
##     combine
```

```
## The following objects are masked from 'package:Matrix':
## 
##     colMeans, colSums, rowMeans, rowSums, which
```

```
## The following objects are masked from 'package:dplyr':
## 
##     combine, intersect, setdiff, union
```

```
## The following objects are masked from 'package:stats':
## 
##     IQR, mad, sd, var, xtabs
```

```
## The following objects are masked from 'package:base':
## 
##     anyDuplicated, append, as.data.frame, cbind, colMeans,
##     colnames, colSums, do.call, duplicated, eval, evalq, Filter,
##     Find, get, grep, grepl, intersect, is.unsorted, lapply,
##     lengths, Map, mapply, match, mget, order, paste, pmax,
##     pmax.int, pmin, pmin.int, Position, rank, rbind, Reduce,
##     rowMeans, rownames, rowSums, sapply, setdiff, sort, table,
##     tapply, union, unique, unsplit, which, which.max, which.min
```

```
## Loading required package: Biobase
```

```
## Welcome to Bioconductor
## 
##     Vignettes contain introductory material; view with
##     'browseVignettes()'. To cite Bioconductor, see
##     'citation("Biobase")', and for packages 'citation("pkgname")'.
```

```
## 
## Attaching package: 'Biobase'
```

```
## The following object is masked from 'package:phyloseq':
## 
##     sampleNames
```

```
## Loading required package: IRanges
```

```
## Loading required package: S4Vectors
```

```
## 
## Attaching package: 'S4Vectors'
```

```
## The following object is masked from 'package:Matrix':
## 
##     expand
```

```
## The following object is masked from 'package:tidyr':
## 
##     expand
```

```
## The following objects are masked from 'package:dplyr':
## 
##     first, rename
```

```
## The following object is masked from 'package:plyr':
## 
##     rename
```

```
## The following objects are masked from 'package:data.table':
## 
##     first, second
```

```
## The following object is masked from 'package:base':
## 
##     expand.grid
```

```
## 
## Attaching package: 'IRanges'
```

```
## The following object is masked from 'package:phyloseq':
## 
##     distance
```

```
## The following objects are masked from 'package:dplyr':
## 
##     collapse, desc, regroup, slice
```

```
## The following object is masked from 'package:plyr':
## 
##     desc
```

```
## The following object is masked from 'package:data.table':
## 
##     shift
```

```
## 
## Attaching package: 'AnnotationDbi'
```

```
## The following object is masked from 'package:dplyr':
## 
##     select
```

```
##
```

```
## Loading required package: treeDA
```

```
##   data.table      stringr         plyr        dplyr        tidyr 
##         TRUE         TRUE         TRUE         TRUE         TRUE 
##      ggplot2      viridis     phyloseq      ggrepel adaptiveGPCA 
##         TRUE         TRUE         TRUE         TRUE         TRUE 
##       Matrix          PMA      mvtnorm    gridExtra RColorBrewer 
##         TRUE         TRUE         TRUE         TRUE         TRUE 
##      svglite       scales        caret       tibble       xtable 
##         TRUE         TRUE         TRUE         TRUE         TRUE 
##     devtools        GO.db     phyloseq       impute       treeDA 
##         TRUE         TRUE         TRUE         TRUE         TRUE
```

```
set.seed(05122016)
rm(list = ls()) # Delete all existing variables
graphics.off() # Close all open plots

# minimal theme for ggplots
theme_set(theme_bw())
min_theme <- theme_update(
  panel.border = element_blank(),
  panel.grid = element_blank(),
  panel.spacing = unit(0, "line"),
  axis.ticks = element_blank(),
  legend.title = element_text(size = 12),
  legend.text = element_text(size = 10),
  axis.text = element_text(size = 10),
  axis.title = element_text(size = 12),
  strip.background = element_blank(),
  strip.text = element_text(size = 10),
  legend.key = element_blank()
)
```

```
ps = readRDS("../data/ps.RDS")
sample_data(ps)$PCR_Pool = as.factor(sample_data(ps)$PCR_Pool)
```

The phyloseq object we read in has all the samples, but for now we only want to look at the ones related to the colon cleanout. We restrict our analysis to those subjects who underwent the colon cleanout (A, B, D, F, G, I, N, and P), and we also exclude some samples from subject A who was planning on doing the colon cleanout but had an unplanned course of antibiotics (these samples are the only ones which are more than 250 days before the cleanout). Below we see the subjects and their sampling schedules for the samples which we will use for the remainder of the analysis.

```
##Fig1
ps = prune_samples(sample_data(ps)$Subject %in%
    paste("AA", c("A", "B", "D", "F", "G", "I", "N", "P"), sep = ""), ps)
ps = prune_samples(sample_data(ps)$CC_RelDay > -250, ps)
ggplot(sample_data(ps)) +
    geom_point(aes(x = CC_RelDay, y = Subject), size = 1) +
    xlab("Days from Cleanout")
```

It is also useful to see the sampling schedule in a bit more detail right around the cleanout, which is shown below.

```
##Fig1-inset
prepost_cols = viridis(12)[c(6, 1)]
ggplot(sample_data(ps)) +
    geom_point(aes(x = CC_RelDay, y = Subject, color = CC_Interval),
               size = 1, alpha = 0.7) +
    scale_color_manual(values = prepost_cols) +
    labs("col" = "Interval", "x" = "Days from Cleanout") +
    xlim(c(-10, 10))
```

## Library size distribution

Because this is a major potential confounder in the data, we’ll first look at how the read depth varies from sample to sample. There are a couple of samples with fewer than 10000 reads that we exclude for having too few reads (these are primarily controls). We’ll also save the library size so that we can include it in later steps.

```
library_size = rowSums(otu_table(ps))
sample_data(ps)$library_size = library_size
qplot(log10(library_size), geom = "histogram")
```

```
## `stat_bin()` using `bins = 30`. Pick better value with `binwidth`.
```

```
ps = prune_samples(library_size > 10000, ps)
```

In the plot above, we see that the library size is bimodal. This bimodality seems to be primarily related to which PCR pool the sample was in, as shown in the plot below. There is still some heterogeneity within PCR pools, but PCR pool seems to account for a very large part of the variance in library size.

```
ggplot(sample_data(ps)) +
    geom_density(aes(color = PCR_Pool, x = log10(library_size)))
```

## Alpha diversity

```
makeAlphaDiversity = function(abund, coverage) {
    relAb = abund / sum(abund)
    relAb = sort(relAb, decreasing = TRUE)
    cumprop = cumsum(relAb)
    min(which(cumprop >= coverage))
}

plot_diversity_coverage = function(abund, samples, coverage, prepost_cols) {
    diversity = apply(abund, 1, makeAlphaDiversity, coverage)
    ggplot(data.frame(diversity, sample_data(ps))) +
      geom_point(aes(x = CC_RelDay, color = CC_Interval, y = diversity)) +
      facet_wrap(~ Subject) +
      scale_color_manual(values = prepost_cols) +
      theme(panel.border = element_rect("transparent", size = 0.15)) +
      labs("x" = "Days from Cleanout", "col" = "Interval") +
      xlim(c(-80, 80))
}

plot_diversity_coverage(otu_table(ps), sample_data(ps), 0.8, prepost_cols) +
    ggtitle("Number of RSVs required for 80% coverage")
```

```
plot_diversity_coverage(otu_table(ps), sample_data(ps), 0.95, prepost_cols) +
    ggtitle("Number of RSVs required for 95% coverage")
```

```
plot_diversity_coverage(otu_table(ps), sample_data(ps), 0.99, prepost_cols) +
    ggtitle("Number of RSVs required for 99% coverage")
```

```
plot_richness(ps, x="CC_RelDay", color="CC_Interval", measures="Shannon") +
    facet_wrap(~ Subject) + xlim(c(-80, 80)) + ggtitle("Shannon") +
    scale_color_manual(values = prepost_cols) +
    labs("x" = "Days from Cleanout", col = "Interval") +
    theme(panel.border = element_rect("transparent", size = 0.15))
```

## Phylum-level analysis

In the OTU data on subjects B, D, F, G, and I, it looked like there was an increase in the fraction of Bacteroidetes or a decrease in the fraction of Firmicutes immediately after the cleanout. This effect was very short-lived and not necessarily that large compared to the standard variance in the fraction of these phyla, and so we want to see if it holds true in the three new subjects A, N, and P for validation purposes. It seems that it does — in the three new subjects we see an increase in the fraction of Bacteroidetes/decrease in the fraction of Firmicutes (more obvious in the Bacteroidetes), and the effect is the strongest when we have data very soon after the cleanout (this is in subject P, where we have a post-cleanout sample on the day of the cleanout).

```
phylum_frac <- function(ps, phylum_name) {
  cur_ix <- which(tax_table(ps)@.Data[, "Phylum"] == phylum_name)
  counts <- get_taxa(ps)[, cur_ix] %>%
    rowSums()

  counts / sample_data(ps)$library_size
}

sample_data(ps)$Firmicutes = phylum_frac(ps, "Firmicutes")
sample_data(ps)$Bacteroidetes = phylum_frac(ps, "Bacteroidetes")
```

```
frac_data <- sample_data(ps) %>%
    filter(abs(CC_RelDay) < 20) %>%
    gather(Phylum, fraction, Firmicutes, Bacteroidetes)

ggplot(frac_data) +
    geom_point(aes(x = CC_RelDay, y = fraction, color = CC_Interval), size = 0.8) +
    scale_color_manual(values = prepost_cols) +
    facet_grid(Phylum ~ Subject) +
    labs("x" = "Days from Cleanout", "col" = "Interval") +
    theme(
      panel.border = element_rect("transparent", size = 0.15),
      axis.text = element_text(size = 7)
    )
```

```
ratio_data <- sample_data(ps) %>%
    filter(abs(CC_RelDay) < 20) %>%
    mutate(ratio = Bacteroidetes / (Bacteroidetes + Firmicutes))

ggplot(ratio_data) +
    geom_point(aes(x = CC_RelDay, y = ratio, color = CC_Interval), size = .8) +
    scale_color_manual(values = prepost_cols) +
    facet_wrap(~ Subject) +
    labs("x" = "Days from Cleanout", "col" = "Interval") +
    theme(
      panel.border = element_rect("transparent", size = 0.15),
      axis.text = element_text(size = 8)
    ) +
    ggtitle("Ratio of Bacteroidetes to\nsum of Bacteroidetes and Firmicutes")
```

## Taxonomic composition over time

We create stacked bar-plots showing composition at the taxonomic family level, in response to reviewer comments. To account for the differential sampling rates, we create one figure aggregating to the weekly level, and another at the daily level (focused immediately around the cleanout).

First, we join the abundances, taxonomic information, and sample data, so we can do the required taxonomic aggregation at the specified time windows.

```
rsv_counts <- data.frame(Meas_ID = sample_names(ps), get_taxa(ps)) %>%
  tbl_df()

sample_df <- sample_data(ps) %>%
  tbl_df()

taxa <- tax_table(ps)@.Data %>%
  tbl_df()
taxa$rsv <- taxa_names(ps)

combined <- rsv_counts %>%
  melt(
    id.vars = "Meas_ID",
    variable.name = "rsv"
  ) %>%
  left_join(sample_df) %>%
  left_join(taxa)
```

```
## Joining, by = "Meas_ID"
```

```
## Joining, by = "rsv"
```

Next, we sum family counts across every week, which is used to compute compositional information.

```
combined$Family[is.na(combined$Family)] <- "unknown"
combined$CC_RelWeek <- round(combined$CC_RelDay / 7)

stacked_bar_data <- combined %>%
  filter(abs(CC_RelWeek) < 12) %>%
  group_by(Subject, CC_RelWeek, Family) %>%
  summarise(family_total = sum(value)) %>%
  group_by(Subject, CC_RelWeek) %>%
  mutate(total = sum(family_total), family_frac = family_total / total)

top_families <- combined %>%
  group_by(Family) %>%
  summarise(family_total = sum(value)) %>%
  arrange(desc(family_total)) %>%
  top_n(7) %>%
  dplyr::select(Family) %>%
  unlist()
```

```
## Selecting by family_total
```

```
## control the plot order in the legend
stacked_bar_data$Family[!(stacked_bar_data$Family %in% top_families)] <- "other"
stacked_bar_data$Family <- factor(
  stacked_bar_data$Family,
  levels = c(setdiff(top_families, "unknown"), "other", "unknown", "unsampled")
)
```

Now we can make the desired week-resolution composition plot.

```
#FigureS1
ggplot(stacked_bar_data) +
  geom_bar(aes(x = CC_RelWeek, y = family_frac, fill = Family), width = 1, stat = "identity") +
  scale_fill_manual(values = c(brewer.pal(8, "Set1"), "#F7F7F7"), drop = FALSE) +
  facet_grid(Subject ~ .) +
  scale_x_continuous(expand=c(0,0), breaks = seq(-8, 8, by = 4)) +
  guides(fill = guide_legend(override.aes = list(color = "black", size = 0.2), nrow = 4)) +
  labs(
    "x" = "Relative Week",
    "y" = "Proportion"
  ) +
  theme(
    panel.border = element_rect(fill = "transparent", size = 1),
    panel.background = element_rect(fill = "#F7F7F7", size = 1),
    legend.position = "bottom",
    axis.text.y = element_blank(),
    legend.text = element_text(size = 9)
  )
```

A similar exercise yields the daily level figure. First, we prepare the input data. The only difference is that we want to keep track of pre / immpost / post for each day so that we can incldue those as facet labels.

```
stacked_bar_data <- combined %>%
  filter(abs(CC_RelDay) < 8) %>%
  group_by(Subject, CC_RelDay, Family) %>%
  summarise(family_total = sum(value), CC_Interval = CC_Interval[1]) %>%
  group_by(Subject, CC_RelDay) %>%
  mutate(
    total = sum(family_total),
    family_frac = family_total / total,
    Pre = CC_Interval == "PreCC",
    ImmPost = CC_Interval == "PostCC" & CC_RelDay <= 3,
    Post = CC_RelDay > 3
  ) %>%
  gather(PreImmpostPost, indicator, Pre, ImmPost, Post) %>%
  filter(indicator)

stacked_bar_data$Family[!(stacked_bar_data$Family %in% top_families)] <- "other"
stacked_bar_data$Family <- factor(
  stacked_bar_data$Family,
  levels = c(setdiff(top_families, "unknown"), "other", "unknown", "unsampled")
)
stacked_bar_data$PreImmpostPost <- factor(
  stacked_bar_data$PreImmpostPost,
  levels = c("Pre", "ImmPost", "Post")
)
```

```
#FigureS2
ggplot(stacked_bar_data) +
  geom_bar(aes(x = CC_RelDay, y = family_frac, fill = Family), width = 1, stat = "identity") +
  scale_fill_manual(values = c(brewer.pal(8, "Set1"), "#F7F7F7"), drop = FALSE) +
  facet_grid(Subject ~ PreImmpostPost, scale = "free_x", space = "free_x") +
  scale_x_continuous(expand=c(0,0), breaks = seq(-6, 6, by = 3)) +
  guides(fill = guide_legend(override.aes = list(color = "black", size = 0.2), nrow = 4)) +
  labs(
    "x" = "Relative Day",
    "y" = "Proportion"
  ) +
  theme(
    axis.text.y = element_blank(),
    strip.text.x = element_text(size = 10),
    panel.border = element_rect(fill = "transparent", size = 1),
    panel.background = element_rect(fill = "#F7F7F7", size = 1),
    legend.position = "bottom",
    legend.text = element_text(size = 9)
  )
```

## Resilience Prediction

Even without discovering the underlying mechanisms of resilience, the development of predictive diagnostics of resilience can be clinically relevant. With only 8 subjects, it is impossible to make any definitive conclusions; however, it is not unreasonable to explore methodological frameworks and propose possibly predictive factors.

One approach to this problem is to define a scalar measure of resilience within each subject, and then attempt to predict this resilience measure using information known before any perturbation is performed. Any pre-perturbation features that may be predictive of resilience could become potential diagnostics.

There are several plausible candidates for characterizing resilience. One natural candidate is the (absolute or relative) change in \(\alpha\)-diversity from the IIOD event to some preset number of days afterwards. The downside of this definition is that individuals for whom the post-IIOD decrease in diversity is most dramatic may appear to be more resilient, because they have more room for improvement. Intuitively, however, these subjects are in fact more vulnerable to change during perturbation. An alternative measure, which we use here, is the relative change in diversity, computed over windows immediately preceding and following and preceding the cleanout, respectively. We use a window of length 3 days.

Before computing the resilience metric, we define the diversity measure. Note that this is the same shannon diversity as plotted in the `plot_richness` command above.

```
shannon_div <- estimate_richness(ps, measures = "shannon")
shannon_div$Meas_ID <- rownames(shannon_div)
shannon_div <- sample_df %>%
  left_join(shannon_div) %>%
  dplyr::select(Meas_ID, Subject, CC_RelDay, Shannon) %>%
  dplyr::rename(CC_RelDay = CC_RelDay, diversity = Shannon)
```

```
## Joining, by = "Meas_ID"
```

We now compute the aforementioned resilience metric. Note that there is some variation across subjects – in fact, some subjects have higher diversity after rather than before the perturbation.

```
diversity_effect <- shannon_div %>%
  filter(abs(CC_RelDay) < 4) %>%
  mutate(interval = ifelse(CC_RelDay > 0, "post", "pre")) %>%
  group_by(Subject, interval) %>%
  summarise(diversity = mean(diversity)) %>%
  ungroup() %>%
  spread(interval, diversity) %>%
  mutate(relative_change = post / pre)

ggplot(diversity_effect) +
  geom_rug(aes(x = relative_change), size = 3) +
  labs("x" = "Relative Change in Diversity")
```

We now consider what predictors might be appropriate as diagnostics for recovery of community diversity following severe perturbation. As there is only limited `sample_data` that appears useful, we instead focus on using initial community composition. Our approach is to compute the family fractions within each subject, across all samples before the three-day window on which the resilience measure is defined. We imagine that certain bacteria might be associated with rapid vs. slow recovery. The block below defines these features and ensures they are aligned with the responses.

```
family_frac <- combined %>%
  filter(CC_RelDay < -4, CC_RelDay > -40) %>%
  group_by(Subject, Family) %>%
  summarise(family_total = sum(value)) %>%
  filter(family_total > 500) %>%
  group_by(Subject) %>%
  mutate(total = sum(family_total), family_frac = family_total / total) %>%
  ungroup() %>%
  dplyr::select(-family_total, -total) %>%
  spread(Family, family_frac, fill = 0)

pred_data <- diversity_effect %>%
  dplyr::select(Subject, relative_change) %>%
  left_join(family_frac)
```

```
## Joining, by = "Subject"
```

```
x <- pred_data %>%
  dplyr::select(-Subject, -relative_change) %>%
  as.matrix()

y <- pred_data %>%
  .[["relative_change"]]
```

Finally, we can fit a model and study (possibly) predictive features. Since there are only 8 subjects, it is hard to say much that isn’t just tentative. Nonetheless, we apply the elastic net and rank features by their importance.

```
fit <- train(x, y, method = "glmnet")
fit
```

```
## glmnet 
## 
##  8 samples
## 38 predictors
## 
## No pre-processing
## Resampling: Bootstrapped (25 reps) 
## Summary of sample sizes: 8, 8, 8, 8, 8, 8, ... 
## Resampling results across tuning parameters:
## 
##   alpha  lambda       RMSE       Rsquared 
##   0.10   0.006195172  0.2198878  0.6351317
##   0.10   0.019590855  0.2181654  0.6363254
##   0.10   0.061951723  0.2067196  0.6361588
##   0.55   0.006195172  0.2450133  0.6042170
##   0.55   0.019590855  0.2341771  0.5910284
##   0.55   0.061951723  0.2045681  0.5405203
##   1.00   0.006195172  0.2838961  0.5990586
##   1.00   0.019590855  0.2637025  0.5755048
##   1.00   0.061951723  0.1967969  0.6076783
## 
## RMSE was used to select the optimal model using  the smallest value.
## The final values used for the model were alpha = 1 and lambda = 0.06195172.
```

```
varImp(fit)
```

```
## glmnet variable importance
## 
##   only 20 most important variables shown (out of 38)
## 
##                              Overall
## Streptococcaceae             100.000
## Enterobacteriaceae            82.883
## Prevotellaceae                 3.837
## Veillonellaceae                0.000
## Acidaminococcaceae             0.000
## Methanomassiliicoccaceae       0.000
## Porphyromonadaceae             0.000
## Leuconostocaceae               0.000
## Synergistaceae                 0.000
## Oxalobacteraceae               0.000
## unknown                        0.000
## Streptophyta                   0.000
## Lachnospiraceae                0.000
## Rikenellaceae                  0.000
## Heliobacteriaceae              0.000
## Bacteroidales_incertae_sedis   0.000
## Enterococcaceae                0.000
## Actinomycetaceae               0.000
## Lactobacillaceae               0.000
## Sutterellaceae                 0.000
```

```
coef_order <- varImp(fit)$importance %>%
                        tibble::rownames_to_column("coef") %>%
                        arrange(desc(Overall)) %>%
                        .[["coef"]]
```

Finally, we plot the raw data for the three features with nonzero importances. Each panel corresponds to a family of bacteria that was found to have nonzero importance in the resilience prediction problem. Within each panel, the initial abundance fraction for that family is plotted along the \(x\)-axis. On the \(y\)-axis is the model’s response – the relative change in diversity between windows immediately before and after the cleanout. The text label is the name of the associated subject. The dashed line corresponds to the situation that diversity does not change between the immediately pre and post cleanout windows.

There is a hint of an association between early presence of these bacteria and change in diversity after cleanout. For example, it seems that when Streptococacceae or Enterobacteriaceae are present at the onset of sampling, diversity actually *increases* post-cleanout, while when Prevotellaceae is more abundant at onset, diversity decreases. Of course, new data would need to be collected to validate these claims.

```
# S8Fig
mpred_data <- pred_data %>%
  melt(id.vars = c("Subject", "relative_change"), variable.name = "coef") %>%
  as_data_frame

filtered_mpred <- mpred_data %>%
  filter(coef %in% coef_order[1:3])
filtered_mpred$coef <- factor(
  filtered_mpred$coef,
  levels = coef_order
)

## Dummy is just to control the x-axis limits separately within each facet.
dummy_df <- data.frame(
  coef = rep(coef_order[1:3], each = 2),
  Subject = rep("AAA", 6),
  value = c(0, 0.008, 0, 0.008, 0, 0.3),
  relative_change = rep(1, 6)
)

ggplot(filtered_mpred, aes(x = value, y = relative_change, label = Subject)) +
  geom_text(hjust = 0) +
  geom_hline(yintercept = 1, size = 0.5, alpha = 0.6, linetype = 2) +
  geom_blank(data = dummy_df) +
  scale_x_continuous(breaks = pretty_breaks(3)) +
  facet_wrap(~coef, scales = "free_x") +
  theme(panel.border = element_rect("transparent", size = 1.5)) +
  labs(
    x = "Pre-Cleanout Community Fraction",
    y = "Relative Change in Diversity"
  )
```

## Ordinations

### Bray-Curtis

Next we are interested in doing some ordinations on this data. We’ll start out with Bray-Curtis as a standard, non-phylogenetic method.

```
transform_compositional = function(ps) {
  otu_table(ps) = otu_table(
    get_taxa(ps) / rowSums(get_taxa(ps)),
    taxa_are_rows = FALSE
  )
 ps
}

ps_comp = transform_compositional(ps)

out.ord = ordinate(ps_comp, method = "PCoA", distance = "bray")
bc.evals = out.ord$values$Eigenvalues
qplot(y = bc.evals / sum(bc.evals), x = seq_along(bc.evals)) +
  labs("x" = "Index", "y" = "Eigenvalue") +
  ggtitle("Scree plot for Bray-Curtis MDS")
```

```
#Fig2A
Fig2A <-plot_ordination(ps_comp, out.ord, color = "Subject" ) + xlim(c(-0.4,0.6))+                                           theme(legend.position="none") + scale_fill_discrete(guide=FALSE)
Fig2A
```

The points in the plot above show mainly a grouping by subject.

From our analysis of the OTU data, we think that the effect of the cleanout is going to be only in the first couple of days, and so we would like to see if those first couple of days separate out well. We first need to make a variable which codes for this, we’ll call `ImmPost'' any`Post’’ samples which are at relative day no more than 3. We make this variable below and plot some of the samples to make sure we did it correctly.

```
samples_time_groups = sample_data(ps) %>%
  mutate(
    Pre = CC_Interval == "PreCC",
    ImmPost = CC_Interval == "PostCC" & CC_RelDay <= 3,
    Post = CC_RelDay > 3
  ) %>%
  gather(PreImmpostPost, indicator, Pre, ImmPost, Post) %>%
  filter(indicator) %>%
  dplyr::select(-indicator)

samples_time_groups$PreImmpostPost = factor(
  samples_time_groups$PreImmpostPost,
  levels = c("Pre", "ImmPost", "Post")
)

row_reorder <- match(samples_time_groups$Meas_ID, rownames(sample_data(ps)))
rownames(samples_time_groups) = samples_time_groups$Meas_ID
sample_data(ps) = samples_time_groups[row_reorder, ]
sample_data(ps_comp) = samples_time_groups[row_reorder, ]

preimmpost_cols <- c("#440154FF", "#DB4551", "#25858EFF")
p1 <- ggplot(sample_data(ps_comp)) +
  geom_point(
    aes(x = CC_RelDay, y = Subject, color = PreImmpostPost),
    size = 1, alpha = 0.5
  ) +
  scale_color_manual(values = preimmpost_cols) +
  scale_x_continuous(expand=c(0,1), limits = c(-20, 20)) +
  theme(
    axis.text = element_blank(),
    axis.title = element_blank(),
    legend.position = "None",
    panel.border = element_rect(fill = "transparent", size = 0.2)
  )

p2 <- ggplot(sample_data(ps_comp)) +
  geom_vline(xintercept = -20, size = 0.2) +
  geom_vline(xintercept = 20, size = 0.2) +
  geom_point(
    aes(x = CC_RelDay, y = Subject, color = PreImmpostPost),
    size = 1, alpha = 0.5
  ) +
  scale_color_manual(values = preimmpost_cols) +
  labs("x" = "Days from Cleanout", "col" = "Interval")

ggsave(file = "make-immpost-1.svg", p1, width = 7 / 2.5, height = 3.5 / 2.5, dpi = 600)
ggsave(file = "make-immpost-2.svg", p2, width = 7, height = 3.5, dpi = 600)
```

Finally, we can plot the Bray-Curtis MDS ordination showing the samples which are in the period immediately after the cleanout. They do not separate out convincingly in this ordination.

```
#FigureS3
ggplot(data.frame(out.ord$vectors, sample_data(ps_comp))) +
  geom_point(aes(x = Axis.1, y = Axis.2, color = PreImmpostPost), size = 1) +
  scale_color_manual(values = preimmpost_cols) +
  facet_wrap(~ Subject) +
  theme(panel.border = element_rect("transparent", size = 0.15)) +
  labs(
    "x" = "Axis 1",
    "y" = "Axis 2",
    "col" = "Interval"
  )
```

```
ggplot(data.frame(out.ord$vectors, sample_data(ps_comp))) +
  geom_point(aes(y = Axis.1, x = CC_RelDay, color = PreImmpostPost), size = .8) +
  scale_color_manual(values = preimmpost_cols) +
  facet_wrap(~ Subject, ncol = 4) +
  theme(panel.border = element_rect("transparent", size = 0.15)) +
  xlim(c(-100, 100)) +
  labs(
    "x" = "Relative Day",
    "y" = "Axis 1",
    "col" = "Interval"
  )
```

```
ggplot(data.frame(out.ord$vectors, sample_data(ps_comp))) +
  geom_point(aes(y = Axis.2, x = CC_RelDay, color = PreImmpostPost), size = .8) +
  scale_color_manual(values = preimmpost_cols) +
  facet_wrap(~ Subject, ncol = 4) +
  theme(panel.border = element_rect("transparent", size = 0.15)) +
  xlim(c(-100, 100)) +
  labs(
    "x" = "Relative Day",
    "y" = "Axis 2",
    "col" = "Interval"
  )
```

### Adaptive gPCA

Next we can look at the results from DPCoA or adaptive gPCA. I’m not putting the plots in this document, but DPCoA does a substantially worse job than either weighted Unifrac or adaptive gPCA at separating the first couple of days post-cleanout from the rest of the samples within each subject.

To do adaptive gPCA and have the transformation be comparable to that in weighted Unifrac, we first log-transform the counts and then transform the log-transformed into proportions. The transformation and the code to perform adaptive gPCA follow.

```
ps_log_cca = transform_sample_counts(ps, function(x) log(1 + x)) %>%
  transform_compositional()

otutab = otu_table(ps_log_cca)
Q = ape::vcv(phy_tree(ps_log_cca))
Q = Q / sum(diag(Q)) * ncol(Q)
Qeig = eigen(Q)
X = scale(otutab, scale = FALSE)
out.adaptive = adaptivegpca(X, Qeig, k = 4)
out.adaptive$r
```

```
## [1] 0.09315285
```

Next we look at the scree plot.

```
adaptive.evals = out.adaptive$vars
qplot(y = adaptive.evals / sum(adaptive.evals), x = seq_along(adaptive.evals)) +
  labs("x" = "Component", "y" = "Fraction of variance") +
  ggtitle("Scree plot for adaptive gPCA")
```

Then we can look at the results colored by subject — the sample points in general for this adaptive gPCA are fairly similar to weighted Unifrac.

```
##Fig2B
perc1 = round(adaptive.evals[1] / sum(adaptive.evals) * 100, digits = 1)
perc2 = round(adaptive.evals[2] / sum(adaptive.evals) * 100, digits = 1)
Fig2B<- ggplot(data.frame(out.adaptive$U, sample_data(ps_log_cca))) +
  geom_point(aes(x = Axis1, y = Axis2,  color = Subject)) +
  xlab(paste0("Axis.1 [", perc1, "%]")) +
  ylab(paste0("Axis.2 [", perc2, "%]"))
```

```
grid.arrange( Fig2A, Fig2B,
    nrow = 2,
    heights = c(0.37,.63)
)
```

If we facet by subject and color by the condition type, we see that the immediate post samples tend to fall at the edge of the clouds of points of their subjects.

```
#Fig3A
fig3a <- ggplot(data.frame(out.adaptive$U, sample_data(ps_log_cca))) +
  geom_point(aes(x = Axis1, y = Axis2,  color = PreImmpostPost), size = .8) +
  scale_color_manual(values = preimmpost_cols) +
  facet_wrap(~ Subject, ncol = 4) +
  labs(
    "x" = paste0("Axis 1: ", perc1, "%"),
    "y" = paste0("Axis 2: ", perc2, "%"),
    "col" = "Interval"
  ) +  theme(panel.border = element_rect("transparent", size = 0.15))
fig3a
```

As before, we can plot the axis 1 score for each sample against time, and again we see that the first couple of samples after the cleanout generally have much higher scores along the first axis than the other samples from the same subject.

```
scoresCentered = out.adaptive$U
for(s in unique(sample_data(ps_log_cca)$Subject)) {
    subjectIdx = which(sample_data(ps_log_cca)$Subject == s)
    scoresCentered[subjectIdx,] = scale(out.adaptive$U[subjectIdx,], scale = FALSE)
}

fig3b <- ggplot(data.frame(scoresCentered, sample_data(ps_log_cca))) +
  geom_point(aes(x = CC_RelDay, y = Axis1,  color = PreImmpostPost), size = .8) +
  scale_color_manual(values = preimmpost_cols) +
  scale_y_continuous(breaks = pretty_breaks(3)) +
  xlim(c(-100, 100)) +
  facet_wrap(~ Subject, ncol = 4) +
  theme(
    panel.border = element_rect("transparent", size = 0.15),
    axis.text = element_text(size = 8)
  ) +
  labs(
    "x" = "Days from Cleanout",
    "y" = paste0("Axis 1: ", perc1, "%"),
    "col" = "Interval"
  )

fig3b
```

And again, a zoomed-in version of the plot above so that we can see the behavior of the samples right around the cleanout.

```
ggplot(data.frame(out.adaptive$U, sample_data(ps_log_cca))) +
  geom_point(aes(x = CC_RelDay, y = Axis1,  color = PreImmpostPost), size = .8) +
  scale_color_manual(values = preimmpost_cols) +
  scale_y_continuous(breaks = pretty_breaks(3)) +
  facet_wrap(~ Subject, scales = "free") + xlim(c(-20, 20)) +
  theme(
    panel.border = element_rect("transparent", size = 0.15),
    axis.text = element_text(size = 8)
  ) +
  labs(
    "x" = "Days from Cleanout",
    "y" = paste0("Axis 1: ", perc1, "%"),
    "col" = "Interval",
    "title" = "Adaptive gPCA"
  )
```

The adaptive gPCA also gives us some insight into the OTUs which explain the axes, as we can see in the plot below. We should probably look at the group of Bacteroidetes which have large scores on the first axis as potentially related to the colon cleanout.

```
#Fig3C
agpca_phyla = data.frame(out.adaptive$QV, tax_table(ps_log_cca)[, c("Phylum", "Genus")])

## Order phyla by abundance, and only label top 7
phyla_order = table(agpca_phyla$Phylum) %>%
  sort(decreasing = TRUE) %>%
  names()
agpca_phyla$Phylum = factor(
  agpca_phyla$Phylum,
  levels = c(phyla_order[1:7], "Other")
)
agpca_phyla$Phylum[is.na(agpca_phyla$Phylum)] <- "Other"

agpca_phyla$Genus <- as.character(agpca_phyla$Genus)
agpca_phyla$Genus[agpca_phyla$Genus != "Bacteroides"] <- "Other"
agpca_phyla$Genus[is.na(agpca_phyla$Genus)] <- "Other"

trimmed_scientific <- function(l) {
  l <- gsub("e-[0-9]+", "", l)
  parse(text = l)
}

fig3c <- ggplot(agpca_phyla) +
  geom_point(
    data = agpca_phyla %>% filter(Phylum == "Other"),
    aes(x = Axis1, y = Axis2),
    size = 1, alpha = 0.6, col = "#5E5E5E"
  ) +
  geom_point(
    data = agpca_phyla %>% filter(Phylum != "Other"),
    aes(x = Axis1, y = Axis2, col = Phylum, shape = Genus),
    size = 1.8
  ) +
  scale_color_brewer(palette = "Set2") +
  scale_shape_manual(values = c(17, 20)) +
  scale_x_continuous(labels = trimmed_scientific) +
  scale_y_continuous(labels = trimmed_scientific) +
  labs(
    "x" = paste0("Axis 1: ", perc1, "%"),
    "y" = paste0("Axis 2: ", perc2, "%"),
    "col" = "Phylum"
  ) + coord_fixed(0.45)
fig3c
```

```
 grid.arrange(fig3a, fig3b, fig3c,
  nrow = 3,
  heights = c(0.8,0.8,1.2)
)
```

```
genusSubset = as.character(tax_table(ps_log_cca)[, "Genus"])
generaToLabel = c(
  "Alistipes", "Bilophila", "Bacteroides", "Roseburia",
  "Eubacterium", "Ruminococcus", "Prevotella", "Bifidobacterium"
)
genusSubset[!(genusSubset %in% generaToLabel)] = "Other"

QV_genus <- data.frame(out.adaptive$QV, genusSubset)
ggplot() +
  geom_point(
    data = QV_genus %>% filter(genusSubset == "Other"),
    aes(x = Axis1, y = Axis2),
    size = 0.2, alpha = 0.2, col = "#5E5E5E"
  ) +
  geom_point(
    data = QV_genus %>% filter(genusSubset != "Other"),
    aes(x = Axis1, y = Axis2, col = genusSubset),
    size = 0.8
  ) +
  scale_color_brewer(palette = "Accent") +
  labs(
    "x" = paste0("Axis 1: ", perc1, "%"),
    "y" = paste0("Axis 2: ", perc2, "%"),
    "col" = "Genus",
    "title" = "Adaptive gPCA: RSVs"
  )
```

## Sparse CCA

Next we want to try sparse CCA to investigate the relationships between the RSV data and the metagenome data. For this analysis, I am going to perform sparse CCA on the metagenome data and the subset of the RSV data containing the genera Prevotella and Bacteroides. This is because those RSVs were associated with the first axis (which seemed to describe the response to the cleanout), and because there is evidence from other studies that the ratio between these two genera is associated with diet and lifestyle differences. Since all the RSVs in the Bacteroides and Prevotella genera only come out to around 100 OTUs, the sparsity penalty will only be on the GO terms, not on the RSVs.

```
## prepare go counts
go_counts = readRDS("../data/go_counts.RDS")
goTotals = Matrix::colSums(go_counts)
go_counts = go_counts[,goTotals > 0]
go_counts = asinh(go_counts)

measids = read.delim(
  "../data/Matrix_Headers_MeasIDs.txt",
  header = TRUE
)

rownames(go_counts) = colnames(measids)[-1]

## prepare metagenomic sample data
source("../src/measToEvent.R")
sampledatamg = measToEvent(
  rownames(go_counts),
  measFile = "../data/Mapping_Files_28Sep2016_Meas.csv",
  eventFile = "../data/Mapping_Files_28Sep2016_Samp.csv"
)
```

```
## check that event codes match, below should be all TRUE
##    Mode    TRUE 
## logical    1103
```

```
## subset to subjects in both datasets
subjects = paste("AA", c("A", "B", "D", "F", "G", "I", "N", "P"), sep = "")
subject_subset = sampledatamg$Subject %in% subjects
go_counts = go_counts[subject_subset,]
sampledatamg = sampledatamg[subject_subset,]

ec = intersect(
  sample_data(ps)$Event_Code,
  sampledatamg$Event_Code.x
) %>%
  unique()

#' Take averages of all measurements with a specific event code
colmeans_in_groups = function(X, groups, groups_map) {
  t(sapply(groups, function(g) {
    Matrix::colMeans(X[groups_map == g,, drop = FALSE])
  }))
}

otusForCCA = which(tax_table(ps)[,"Genus"] == "Bacteroides")

cca_otus = colmeans_in_groups(
  otu_table(ps)[, otusForCCA],
  ec,
  sample_data(ps)$Event_Code
)

cca_metag = colmeans_in_groups(
  go_counts,
  ec,
  sampledatamg$Event_Code.x
)

## make a sample data table for the event codes
keep_cols = c(
  "Subject", "CC_RelDay", "CC_Interval", "Event_Code",
  "PreImmpostPost", "PCR_Pool"
)

cca_sampledata = sample_data(ps) %>%
  dplyr::select_(.dots = keep_cols) %>%
  unique() %>%
  filter(
    Event_Code %in% ec,
    !(Subject == "AAB" & PCR_Pool == 2)
  ) %>%
  mutate(Event_Code = as.character(Event_Code))

cca_otus = cca_otus[cca_sampledata$Event_Code, ]
cca_metag = cca_metag[cca_sampledata$Event_Code, ]

## filter out any otus / GO terms which are always 0
cca_otus = cca_otus[, colSums(cca_otus) != 0]
cca_metag = cca_metag[, colSums(cca_metag) != 0]
```

```
cca_otus_trans = asinh(cca_otus)
for(s in unique(cca_sampledata$Subject)) {
    idx = which(cca_sampledata$Subject == s)
    cca_otus_trans[idx,] = scale(cca_otus_trans[idx,], scale = FALSE)
}
out.cca = CCA(cca_otus_trans, cca_metag, K = 2, penaltyx = 1, penaltyz = .1, niter = 40)
```

```
## 12345678
## 12345678910111213141516
```

Here we see first the RSV scores and then the sample scores along the first two CCA axes. We see that the samples lie along a gradient, and that the samples immediately after the cleanout are associated with one end of that gradient.

```
#S4-Fig-cca
tt = tax_table(ps)[colnames(cca_otus),]
df = data.frame(out.cca$u, tt)
df$rsv <- paste0("RSV", match(rownames(df), taxa_names(ps)))

ggplot(df) +
  geom_text(aes(x = X1, y = X2, label = rsv), size = 3) +
  labs(
    "x" = sprintf("c1(X) [d1 = %s]", comma(round(out.cca$d[1], -2))),
    "y" = sprintf("c2(X) [d2 = %s]", comma(round(out.cca$d[2], -2)))
  ) +
  coord_fixed(sqrt(out.cca$d[2] / out.cca$d[1]))
```

```
scores = cca_otus %*% out.cca$u
ggplot(data.frame(scores, cca_sampledata)) +
  geom_point(aes(x = X1, y = X2, color = PreImmpostPost)) + facet_wrap(~ Subject) +
  scale_x_continuous(breaks = c(-35000, 0)) +
  scale_y_continuous(breaks = pretty_breaks(3)) +
  scale_color_manual(values = preimmpost_cols) +
  labs(
    "x" = sprintf("Xc1 [d1 = %s]", comma(round(out.cca$d[1], -2))),
    "y" = sprintf("Xc2 [d2 = %s]", comma(round(out.cca$d[2], -2))),
    "col" = "Interval"
  ) +
  theme(panel.border = element_rect("transparent", size = 0.15)) +
  coord_fixed(sqrt(out.cca$d[2] / out.cca$d[1]))
```

Below are a list of the GO terms associated with the first and second CCA axes, listed in order of magnitude.

```
#Fig5
terms_coord = data.table(
  term = Term(colnames(cca_metag)),
  scores = out.cca$v
) %>%
  dplyr::rename(Yc1 = scores.V1, Yc2 = scores.V2) %>%
  filter(Yc1 != 0 | Yc2 != 0)

## shorten some of the long names
terms_coord$term <- gsub(
  "metabolic process",
  "m.p.",
  terms_coord$term
)

terms_coord$term <- gsub(
  "catabolic process",
  "c.p.",
  terms_coord$term
)

terms_coord$term <- revalue(
  terms_coord$term,
  c(
    "pyruvate family amino acid c.p." = "pyruvate amino acid c.p.",
    "regulation of translational elongation" = "translational elongation",
    "cellular transition metal ion homeostasis" = "transition metal ion"
  )
)

## merge terms for very close by points
text_coord <- terms_coord %>%
  filter(Yc1 < -0.23 | Yc2  > 0.1 | Yc2 < -0.25) %>%
  arrange(Yc1, Yc2) %>%
  mutate(group = 1)

j <- 2
for (i in seq_len(nrow(text_coord) - 1)) {
  cur_dist <- dist(text_coord[c(i, i + 1), c("Yc1", "Yc2")])
  if (cur_dist > 0.005) {
    j <- j + 1
  }
  text_coord[i + 1, "group"] <- j
}

text_coord <- text_coord %>%
  group_by(group) %>%
  summarise(
    new_term = paste0(term, collapse = "\n "),
    Yc1 = mean(Yc1),
    Yc2 = mean(Yc2)
  )

## create the figure
ggplot() +
  geom_label_repel(
    data = text_coord,
    aes(x = Yc1, y = Yc2, label = new_term),
    size = 3,
    force = 10,
    segment.size = 0.5,
    segment.alpha = 0.3,
    nudge_y = 0.02,
    nudge_x = -0.05
  )  +
  geom_point(
    data = terms_coord,
    aes(x = Yc1, y = Yc2), size = 1
  ) +
  scale_x_continuous(limits = c(-0.5, 0.01)) +
  scale_y_continuous(limits = c(-0.385, 0.25)) +
  labs(
    "x" = sprintf("c1(Y) [d1 = %s]", comma(round(out.cca$d[1], -2))),
    "y" = sprintf("c2(Y) [d2 = %s]", comma(round(out.cca$d[2], -2)))
  ) +
  coord_fixed(sqrt(out.cca$d[2] / out.cca$d[1]))
```

```
## print all terms
factor_terms = function(term_names, factors) {
  axis_terms = data.table(
    Term = Term(term_names[factors != 0]),
    value = factors[factors != 0]
  )

  setkey(axis_terms, "value")
  axis_terms
}

options(width = 120)
factor_terms(colnames(cca_metag), out.cca$v[, 1])
```

```
##                                                           Term         value
##  1:                                cellular cation homeostasis -0.2697365415
##  2:                             cellular metal ion homeostasis -0.2695466457
##  3:                  cellular transition metal ion homeostasis -0.2661911159
##  4:                                           response to heat -0.2608229397
##  5:                                  formate metabolic process -0.2529806507
##  6:                                histidine catabolic process -0.2528827444
##  7:                                formamide metabolic process -0.2528827444
##  8:            imidazole-containing compound catabolic process -0.2528827444
##  9:                                L-alanine metabolic process -0.2362943833
## 10:                                  alanine catabolic process -0.2314990062
## 11:               pyruvate family amino acid catabolic process -0.2314990062
## 12:                               pyridoxine metabolic process -0.2009044398
## 13:                            vitamin B6 biosynthetic process -0.2009044398
## 14:                      cellular response to abiotic stimulus -0.1834706329
## 15:                                 response to osmotic stress -0.1828590520
## 16:                                       iron ion homeostasis -0.1621625103
## 17:                                          peptide transport -0.1208165260
## 18:                               antibiotic catabolic process -0.1142521103
## 19:                   beta-lactam antibiotic metabolic process -0.1142521103
## 20:                             galacturonan metabolic process -0.0926286301
## 21:                                   pectin metabolic process -0.0926286301
## 22:                                            lipid transport -0.0858178769
## 23:                                         lipid localization -0.0835189114
## 24:                          GDP-L-fucose biosynthetic process -0.0813604831
## 25:                             GDP-L-fucose metabolic process -0.0813604831
## 26:                                     copper ion homeostasis -0.0778677888
## 27:                                      telomere organization -0.0733201001
## 28:                           anatomical structure homeostasis -0.0733201001
## 29:                                           response to drug -0.0653423029
## 30:                                             drug transport -0.0653423029
## 31:                            fatty acid biosynthetic process -0.0488578764
## 32:                                 arabinan metabolic process -0.0422072224
## 33:                                 cell envelope organization -0.0358594481
## 34:                                          membrane assembly -0.0358594481
## 35:                                                  cytolysis -0.0345824646
## 36:                                     cell wall organization -0.0303905344
## 37: modification of morphology or physiology of other organism -0.0297625507
## 38:                         killing of cells of other organism -0.0292197161
## 39:                      disruption of cells of other organism -0.0292197161
## 40:                                  lipoate metabolic process -0.0201417406
## 41:                                   carbohydrate homeostasis -0.0053143003
## 42:                                        glucose homeostasis -0.0053143003
## 43:                                       cellular homeostasis -0.0002851044
##                                                           Term         value
```

```
factor_terms(colnames(cca_metag), out.cca$v[, 2])
```

```
##                                                              Term        value
##  1:                         tetrahydrobiopterin metabolic process -0.380421916
##  2:                         N-acetylglucosamine metabolic process -0.281095191
##  3:                               galacturonate metabolic process -0.228491225
##  4:                             D-galacturonate metabolic process -0.228491225
##  5:                               galacturonate catabolic process -0.228491225
##  6:                       pyrimidine nucleotide catabolic process -0.210118332
##  7:          pyrimidine nucleoside triphosphate catabolic process -0.210118332
##  8: pyrimidine deoxyribonucleoside triphosphate catabolic process -0.210118332
##  9:              pyrimidine deoxyribonucleotide catabolic process -0.210118332
## 10:                     nucleoside triphosphate catabolic process -0.192860539
## 11:            deoxyribonucleoside triphosphate catabolic process -0.192860539
## 12:                         deoxyribonucleotide catabolic process -0.192860539
## 13:                       deoxyribose phosphate catabolic process -0.192860539
## 14:                                      carbohydrate homeostasis -0.180823377
## 15:                                           glucose homeostasis -0.180823377
## 16:                          regulation of translational fidelity -0.159945886
## 17:                                  glyoxylate metabolic process -0.157444967
## 18:                   response to topologically incorrect protein -0.153410808
## 19:                      response to arsenic-containing substance -0.120439918
## 20:              pyrimidine deoxyribonucleoside metabolic process -0.119283131
## 21:                                                  cell killing -0.100538589
## 22:                                      DNA biosynthetic process -0.097080398
## 23:                                        sodium ion homeostasis -0.096649047
## 24:                                   non-glycolytic fermentation -0.087932870
## 25:                                  propionate metabolic process -0.082959817
## 26:                       monovalent inorganic cation homeostasis -0.072110754
## 27:                                          cellular homeostasis -0.039383002
## 28:                    phosphatidylethanolamine metabolic process -0.029984218
## 29:   CMP-keto-3-deoxy-D-manno-octulosonic acid metabolic process -0.017278764
## 30:                      short-chain fatty acid metabolic process -0.017209183
## 31:                                     quinone metabolic process -0.013582955
## 32:                                  quinone biosynthetic process -0.013582955
## 33:                                 menaquinone metabolic process -0.012353450
## 34:                   reactive nitrogen species metabolic process -0.001377018
## 35:                    N-terminal protein amino acid modification  0.025177777
## 36:                                           protein acetylation  0.044904947
## 37:                                             protein acylation  0.050251020
## 38:                               teichoic acid metabolic process  0.103981577
## 39:                        regulation of translational elongation  0.153056672
## 40:                                           cytokinetic process  0.185889653
##                                                              Term        value
```

```
options(width = 80)
```

```
#Fig6
ggplot(
  data.frame(
    cca_sampledata,
    Xc1 = cca_otus_trans %*% out.cca$u[, 1],
    Yc1 = cca_metag %*% out.cca$v[, 1]
  )) +
  geom_point(aes(x = Xc1, y = Yc1, col = PreImmpostPost), size = 1.5) +
  scale_color_manual(values = preimmpost_cols) +
  labs(
    "col" = "Interval",
    "x" = sprintf("X * c1(X) [d1 = %s]", comma(round(out.cca$d[1], -2))),
    "y" = sprintf("Y * c1(Y) [d1 = %s]", comma(round(out.cca$d[1], -2)))
  ) +
  coord_fixed()
```

The following is a helper function to get the legend from a ggplot, and is taken from the STHDA website here.

```
get_legend<-function(myggplot){
  tmp <- ggplot_gtable(ggplot_build(myggplot))
  leg <- which(sapply(tmp$grobs, function(x) x$name) == "guide-box")
  legend <- tmp$grobs[[leg]]
  return(legend)
}
```

```
#Fig4
scores = cca_metag %*% out.cca$v
cca.scores.1 = ggplot(data.frame(scores, cca_sampledata)) +
  geom_point(aes(x = CC_RelDay, y = X1, color = PreImmpostPost), size = .8) +
  scale_color_manual(values = preimmpost_cols) +
  facet_wrap(~ Subject, scales = "free_x", ncol = 2) +
  theme(panel.border = element_rect("transparent", size = 0.15)) +
  labs("x" = "Days from Cleanout", y = "Score on CCA Axis 1", col = "Interval")
cca.scores.2 = ggplot(data.frame(scores, cca_sampledata)) +
  geom_point(aes(x = CC_RelDay, y = X2, color = PreImmpostPost), size = .8) +
  scale_color_manual(values = preimmpost_cols) +
  facet_wrap(~ Subject, scales = "free_x", ncol = 2) +
  theme(panel.border = element_rect("transparent", size = 0.15)) +
  labs("x" = "Days from Cleanout", y = "Score on CCA Axis 2", col = "Interval")
grid.arrange(cca.scores.1 + theme(legend.position = "none"),
             cca.scores.2 + theme(legend.position = "none"),
             get_legend(cca.scores.1),
             ncol = 3,
             widths = c(1,1,.2))
```

## Sparse Discriminant Analysis

```
ps_sub = prune_samples(sample_data(ps)$PreImmpostPost != "Post", ps)
ps_sub = prune_taxa(colSums(otu_table(ps_sub) > 5) > 10, ps_sub)


subjects = unique(sample_data(ps_sub)$Subject)
X = log(1 + otu_table(ps_sub))
for(s in subjects) {
    idx = which(sample_data(ps_sub)$Subject == s)
    X[idx, ] = scale(X[idx, ], scale = FALSE)
}

## Prepare input / output structures for treeda
pvec = 1:50
cvloss = matrix(NA, nrow = length(pvec), ncol = length(subjects))
colnames(cvloss) = subjects
A = treeDA:::makeDescendantMatrix(phy_tree(ps_sub))

for(i in seq_along(pvec)) {
    if (i != 25) { ## this is where minimum occurs (you can rerun cv by commenting this out)
      next
    }
    for(s in subjects) {
        cat(sprintf("Training \t subject: %s \t p: %s\n", s, pvec[i]))

        trainidx = which(sample_data(ps_sub)$Subject != s)
        out.treeda = treeda(
          sample_data(ps_sub)$PreImmpostPost[trainidx],
          X[trainidx,],
          phy_tree(ps_sub),
          p = pvec[i],
          check.consist = FALSE,
          scale = FALSE,
          A = A
        )

        predictions = predict(out.treeda, X[-trainidx,])
        cvloss[i,s] = sum(predictions$classes != sample_data(ps_sub)$PreImmpostPost[-trainidx])
    }
}
```

```
## Training      subject: AAA    p: 25
## Training      subject: AAB    p: 25
## Training      subject: AAD    p: 25
## Training      subject: AAF    p: 25
## Training      subject: AAG    p: 25
## Training      subject: AAI    p: 25
## Training      subject: AAN    p: 25
## Training      subject: AAP    p: 25
```

```
##Fig7A
treeda.final = treeda(
    sample_data(ps_sub)$PreImmpostPost,
    X,
    phy_tree(ps_sub),
    p = which.min(rowSums(cvloss)),
    check.consist = FALSE,
    A = A
)

fig7a <- ggplot(data.frame(proj = treeda.final$projections, sample_data(ps_sub))) +
    geom_point(aes(x = proj, y = Subject, color = PreImmpostPost)) +
    scale_color_manual(values = preimmpost_cols) +
    xlab("Score")

fig7a
```

Here we look at the performance on the held-out data. For the optimal value of p, we fit the model on all but one of the subjects and then find the projections of the held out subject onto the discriminating axis defined by the others. We repeat this procedure for each subject and plot the results.

```
#FigS7
projectionList = list()
for(s in subjects) {
    idx = which(sample_data(ps_sub)$Subject == s)
    treeda.heldout = treeda(
        sample_data(ps_sub)$PreImmpostPost[-idx],
        X[-idx,],
        phy_tree(ps_sub),
        p = which.min(rowSums(cvloss)),
        check.consist = FALSE,
        A = A
    )
    projectionList[[s]] = data.frame(
                      projection = predict(treeda.heldout, newdata = X[idx,])$projections,
                      sample_data(ps_sub)[idx,]
    )
}
heldOutProjections = Reduce(rbind, projectionList)
ggplot(heldOutProjections) +
    geom_point(aes(y = Axis.1, x = CC_RelDay, color = PreImmpostPost)) +
    facet_wrap(~Subject, ncol = 4) + xlim(c(-100, 4))
```

```
#Fig7B
tree.mod = phy_tree(ps_sub)
tree.mod$edge.length[tree.mod$edge.length > .7] = .7
generaToLabelDA = c("Bacteroides", "Roseburia", "Ruminococcus", "Oxalobacter",
                    "Clostridium_XlVb", "Parasutterella", "Sutterella")
genusSubsetDA = as.vector(tax_table(ps_sub)[,"Genus"])
genusSubsetDA[!(genusSubsetDA %in% generaToLabelDA)] = "Other"

otu_position = get_leaf_position(tree.mod, ladderize = TRUE)$otu.pos
coorddf = data.frame(
    x = otu_position,
    y = as(treeda.final$leafCoefficients$beta, "matrix"),
    Genus = genusSubsetDA
)

coorddf = subset(coorddf, y != 0)
values = c(brewer.pal(n = 8, "Set1")[-6], "bisque4")
names(values) = c(generaToLabelDA, "Other")
coorddf$Genus = factor(coorddf$Genus, levels = names(values), ordered = TRUE)

rsvplot = ggplot(coorddf) +
  geom_hline(aes(yintercept = 0), color = "gray") +
  geom_point(aes(x = x, y = y, color = Genus), size = 1) +
  scale_color_manual(values = values) +
  theme(axis.title.x=element_blank(), axis.text.x=element_blank(),
        axis.ticks.x=element_blank()) +
  ylab("beta") +
  scale_x_continuous(limits = c(min(otu_position), max(otu_position)))
treeplot = plot_tree(tree.mod, ladderize = TRUE) +
    coord_flip() +
    scale_x_reverse()

fullplot = combine_plot_and_tree(
  rsvplot + theme(legend.position = "none"),
  treeplot,
  tree.height = 1
)
```

```
fig7b <- grid.arrange(
    fullplot,
    get_legend(rsvplot),
    ncol = 2,
    widths = c(1,.3)
)
```

```
fig7b
```

```
## TableGrob (1 x 2) "arrange": 2 grobs
##   z     cells    name              grob
## 1 1 (1-1,1-1) arrange    gtable[layout]
## 2 2 (1-1,2-2) arrange gtable[guide-box]
```

We can look at what the taxa are in some of the larger groups of RSVs which were selected. We see that the largest is a group of 16 Ruminococcus RSVs,

```
#Fig7C
beta = as.vector(treeda.final$leafCoefficients$beta)
leafIndices = list()

idx = 1
for(v in unique(beta)) {
    leafIndices[[idx]] = list(
      value = v,
      indices = which(beta == v),
      size = sum(beta == v)
    )
    idx = idx + 1
}

which(sapply(leafIndices, function(x) x$size) >= 10)
```

```
## [1]  1  4 11 15
```

```
ttnorownames = tax_table(ps_sub)
rownames(ttnorownames) = NULL
ttnorownames[leafIndices[[4]]$indices]
```

```
## Taxonomy Table:     [16 taxa by 6 taxonomic ranks]:
##      Kingdom    Phylum       Class        Order          
## sp1  "Bacteria" "Firmicutes" "Clostridia" "Clostridiales"
## sp2  "Bacteria" "Firmicutes" "Clostridia" "Clostridiales"
## sp3  "Bacteria" "Firmicutes" "Clostridia" "Clostridiales"
## sp4  "Bacteria" "Firmicutes" "Clostridia" "Clostridiales"
## sp5  "Bacteria" "Firmicutes" "Clostridia" "Clostridiales"
## sp6  "Bacteria" "Firmicutes" "Clostridia" "Clostridiales"
## sp7  "Bacteria" "Firmicutes" "Clostridia" "Clostridiales"
## sp8  "Bacteria" "Firmicutes" "Clostridia" "Clostridiales"
## sp9  "Bacteria" "Firmicutes" "Clostridia" "Clostridiales"
## sp10 "Bacteria" "Firmicutes" "Clostridia" "Clostridiales"
## sp11 "Bacteria" "Firmicutes" "Clostridia" "Clostridiales"
## sp12 "Bacteria" "Firmicutes" "Clostridia" "Clostridiales"
## sp13 "Bacteria" "Firmicutes" "Clostridia" "Clostridiales"
## sp14 "Bacteria" "Firmicutes" "Clostridia" "Clostridiales"
## sp15 "Bacteria" "Firmicutes" "Clostridia" "Clostridiales"
## sp16 "Bacteria" "Firmicutes" "Clostridia" "Clostridiales"
##      Family            Genus         
## sp1  "Ruminococcaceae" "Ruminococcus"
## sp2  "Ruminococcaceae" "Ruminococcus"
## sp3  "Ruminococcaceae" "Ruminococcus"
## sp4  "Ruminococcaceae" "Ruminococcus"
## sp5  "Ruminococcaceae" "Ruminococcus"
## sp6  "Ruminococcaceae" "Ruminococcus"
## sp7  "Ruminococcaceae" "Ruminococcus"
## sp8  "Ruminococcaceae" "Ruminococcus"
## sp9  "Ruminococcaceae" "Ruminococcus"
## sp10 "Ruminococcaceae" "Ruminococcus"
## sp11 "Ruminococcaceae" "Ruminococcus"
## sp12 "Ruminococcaceae" "Ruminococcus"
## sp13 "Ruminococcaceae" "Ruminococcus"
## sp14 "Ruminococcaceae" "Ruminococcus"
## sp15 "Ruminococcaceae" "Ruminococcus"
## sp16 "Ruminococcaceae" "Ruminococcus"
```

```
ttnorownames[leafIndices[[11]]$indices]
```

```
## Taxonomy Table:     [12 taxa by 6 taxonomic ranks]:
##      Kingdom    Phylum           Class                 Order            
## sp1  "Bacteria" "Proteobacteria" "Betaproteobacteria"  "Burkholderiales"
## sp2  "Bacteria" "Proteobacteria" "Betaproteobacteria"  "Burkholderiales"
## sp3  "Bacteria" "Proteobacteria" "Betaproteobacteria"  "Burkholderiales"
## sp4  "Bacteria" "Proteobacteria" "Gammaproteobacteria" "Pasteurellales" 
## sp5  "Bacteria" "Proteobacteria" "Betaproteobacteria"  "Burkholderiales"
## sp6  "Bacteria" "Proteobacteria" "Betaproteobacteria"  "Burkholderiales"
## sp7  "Bacteria" "Proteobacteria" "Betaproteobacteria"  "Burkholderiales"
## sp8  "Bacteria" "Proteobacteria" "Betaproteobacteria"  "Burkholderiales"
## sp9  "Bacteria" "Proteobacteria" "Betaproteobacteria"  "Burkholderiales"
## sp10 "Bacteria" "Proteobacteria" "Betaproteobacteria"  "Burkholderiales"
## sp11 "Bacteria" "Proteobacteria" "Betaproteobacteria"  "Burkholderiales"
## sp12 "Bacteria" "Proteobacteria" "Betaproteobacteria"  "Burkholderiales"
##      Family             Genus           
## sp1  "Sutterellaceae"   "Parasutterella"
## sp2  "Sutterellaceae"   "Sutterella"    
## sp3  NA                 NA              
## sp4  "Pasteurellaceae"  "Mannheimia"    
## sp5  NA                 NA              
## sp6  "Sutterellaceae"   "Sutterella"    
## sp7  "Oxalobacteraceae" "Oxalobacter"   
## sp8  "Sutterellaceae"   NA              
## sp9  "Sutterellaceae"   "Parasutterella"
## sp10 "Oxalobacteraceae" "Oxalobacter"   
## sp11 "Sutterellaceae"   "Sutterella"    
## sp12 "Oxalobacteraceae" NA
```

```
ttnorownames[leafIndices[[15]]$indices]
```

```
## Taxonomy Table:     [11 taxa by 6 taxonomic ranks]:
##      Kingdom    Phylum       Class        Order           Family Genus
## sp1  "Bacteria" "Firmicutes" "Clostridia" "Clostridiales" NA     NA   
## sp2  "Bacteria" "Firmicutes" "Clostridia" "Clostridiales" NA     NA   
## sp3  "Bacteria" "Firmicutes" "Clostridia" "Clostridiales" NA     NA   
## sp4  "Bacteria" "Firmicutes" NA           NA              NA     NA   
## sp5  "Bacteria" "Firmicutes" NA           NA              NA     NA   
## sp6  "Bacteria" "Firmicutes" NA           NA              NA     NA   
## sp7  "Bacteria" "Firmicutes" NA           NA              NA     NA   
## sp8  "Bacteria" "Firmicutes" NA           NA              NA     NA   
## sp9  "Bacteria" "Firmicutes" "Clostridia" "Clostridiales" NA     NA   
## sp10 "Bacteria" "Firmicutes" NA           NA              NA     NA   
## sp11 "Bacteria" NA           NA           NA              NA     NA
```

```
ps_sub2 = prune_taxa(taxa_names(ps) %in% taxa_names(ps_sub), ps)
otudf = log(1 + get_sample(ps_sub2, leafIndices[[4]]$indices))
dfm = data.frame(otudf, sample_data(ps_sub2)) %>%
    melt(id.vars = colnames(sample_data(ps_sub2)))

map = paste0("RSV", match(unique(dfm$variable), taxa_names(ps)))
names(map) = unique(dfm$variable)
dfm$RSV = factor(map[dfm$variable], levels = map)
fig7c <- ggplot(dfm) +
    geom_point(aes(x = CC_RelDay, y = value, color = PreImmpostPost), size = .8) +
    geom_line(aes(x = CC_RelDay, y = value, group = Subject, color = PreImmpostPost)) +
    scale_x_continuous(breaks = pretty_breaks(3)) +
    scale_y_continuous(breaks = pretty_breaks(3)) +
    facet_wrap(~ RSV, scales = "free_y") +
    xlim(c(-20, 20)) +
    labs(x = "Days from Cleanout", y = "log(1 + abundance)", col = "Interval") +
    scale_color_manual(values = preimmpost_cols) +
    theme(panel.border = element_rect("transparent", size = 0.15))
```

```
## Scale for 'x' is already present. Adding another scale for 'x', which
## will replace the existing scale.
```

```
fig7c
```

```
 grid.arrange(fig7a, fig7b, fig7c,
  nrow = 3,
  heights = c(0.9,0.9,1)
)
```

```
rsv_identity <- tax_table(ps) %>%
  as.data.frame %>%
  rownames_to_column(var = "sequence") %>%
  as_data_frame(keep_rownames = TRUE) %>%
  mutate(rsv_ix = 1:n()) %>%
  mutate(rsv_ix = paste0("RSV", rsv_ix)) %>%
  dplyr::select(rsv_ix, Kingdom, Phylum, Class, Order, Family, Genus, sequence) %>%
  mutate(
    treeda_selected = sequence %in% dfm$variable,
    cca_selected = sequence %in% rownames(tt)
  ) %>%
  arrange(desc(treeda_selected), desc(cca_selected))

write.csv(rsv_identity, file = "../data/rsv_identity.csv", row.names = FALSE)
print(xtable(rsv_identity, caption = "The mapping between RSV numbers displayed in figures and their                      sequence identity."),
                                    file = "../data/rsv_identity.tex")
```

### Tree-DA with asinh transformation

Here we try tree discriminant analysis with a different data transformation to see how sensitive the results are to the specific transformation used. We’re going to try the asinh transformation, which is similar to the log(1+x) transformation at the low and high end but has a slightly different behavior in the middle. The results are qualitatively similar: the model chosen is a bit more parsimonious this time. The results are broadly the same though: the largest clade identified is composed of Ruminococcus RSVs which are negatively associated with the immediate post period, and a group of Bacteroides are identified as positively associated with the immediate post period.

```
X = asinh(otu_table(ps_sub))
for(s in subjects) {
    idx = which(sample_data(ps_sub)$Subject == s)
    X[idx, ] = scale(X[idx, ], scale = FALSE)
}

## Prepare input / output structures for treeda
pvec = 1:50
cvloss = matrix(NA, nrow = length(pvec), ncol = length(subjects))
colnames(cvloss) = subjects
A = treeDA:::makeDescendantMatrix(phy_tree(ps_sub))

for(i in seq_along(pvec)) {
    for(s in subjects) {
        cat(sprintf("Training \t subject: %s \t p: %s\n", s, pvec[i]))

        trainidx = which(sample_data(ps_sub)$Subject != s)
        out.treeda = treeda(
          sample_data(ps_sub)$PreImmpostPost[trainidx],
          X[trainidx,],
          phy_tree(ps_sub),
          p = pvec[i],
          check.consist = FALSE,
          scale = FALSE,
          A = A
        )

        predictions = predict(out.treeda, X[-trainidx,])
        cvloss[i,s] = sum(predictions$classes != sample_data(ps_sub)$PreImmpostPost[-trainidx])
    }
}
```

```
## Training      subject: AAA    p: 1
## Training      subject: AAB    p: 1
## Training      subject: AAD    p: 1
## Training      subject: AAF    p: 1
## Training      subject: AAG    p: 1
## Training      subject: AAI    p: 1
## Training      subject: AAN    p: 1
## Training      subject: AAP    p: 1
## Training      subject: AAA    p: 2
## Training      subject: AAB    p: 2
## Training      subject: AAD    p: 2
## Training      subject: AAF    p: 2
## Training      subject: AAG    p: 2
## Training      subject: AAI    p: 2
## Training      subject: AAN    p: 2
## Training      subject: AAP    p: 2
## Training      subject: AAA    p: 3
## Training      subject: AAB    p: 3
## Training      subject: AAD    p: 3
## Training      subject: AAF    p: 3
## Training      subject: AAG    p: 3
## Training      subject: AAI    p: 3
## Training      subject: AAN    p: 3
## Training      subject: AAP    p: 3
## Training      subject: AAA    p: 4
## Training      subject: AAB    p: 4
## Training      subject: AAD    p: 4
## Training      subject: AAF    p: 4
## Training      subject: AAG    p: 4
## Training      subject: AAI    p: 4
## Training      subject: AAN    p: 4
## Training      subject: AAP    p: 4
## Training      subject: AAA    p: 5
## Training      subject: AAB    p: 5
## Training      subject: AAD    p: 5
## Training      subject: AAF    p: 5
## Training      subject: AAG    p: 5
## Training      subject: AAI    p: 5
## Training      subject: AAN    p: 5
## Training      subject: AAP    p: 5
## Training      subject: AAA    p: 6
## Training      subject: AAB    p: 6
## Training      subject: AAD    p: 6
## Training      subject: AAF    p: 6
## Training      subject: AAG    p: 6
## Training      subject: AAI    p: 6
## Training      subject: AAN    p: 6
## Training      subject: AAP    p: 6
## Training      subject: AAA    p: 7
## Training      subject: AAB    p: 7
## Training      subject: AAD    p: 7
## Training      subject: AAF    p: 7
## Training      subject: AAG    p: 7
## Training      subject: AAI    p: 7
## Training      subject: AAN    p: 7
## Training      subject: AAP    p: 7
## Training      subject: AAA    p: 8
## Training      subject: AAB    p: 8
## Training      subject: AAD    p: 8
## Training      subject: AAF    p: 8
## Training      subject: AAG    p: 8
## Training      subject: AAI    p: 8
## Training      subject: AAN    p: 8
## Training      subject: AAP    p: 8
## Training      subject: AAA    p: 9
## Training      subject: AAB    p: 9
## Training      subject: AAD    p: 9
## Training      subject: AAF    p: 9
## Training      subject: AAG    p: 9
## Training      subject: AAI    p: 9
## Training      subject: AAN    p: 9
## Training      subject: AAP    p: 9
## Training      subject: AAA    p: 10
## Training      subject: AAB    p: 10
## Training      subject: AAD    p: 10
## Training      subject: AAF    p: 10
## Training      subject: AAG    p: 10
## Training      subject: AAI    p: 10
## Training      subject: AAN    p: 10
## Training      subject: AAP    p: 10
## Training      subject: AAA    p: 11
## Training      subject: AAB    p: 11
## Training      subject: AAD    p: 11
## Training      subject: AAF    p: 11
## Training      subject: AAG    p: 11
## Training      subject: AAI    p: 11
## Training      subject: AAN    p: 11
## Training      subject: AAP    p: 11
## Training      subject: AAA    p: 12
## Training      subject: AAB    p: 12
## Training      subject: AAD    p: 12
## Training      subject: AAF    p: 12
## Training      subject: AAG    p: 12
## Training      subject: AAI    p: 12
## Training      subject: AAN    p: 12
## Training      subject: AAP    p: 12
## Training      subject: AAA    p: 13
## Training      subject: AAB    p: 13
## Training      subject: AAD    p: 13
## Training      subject: AAF    p: 13
## Training      subject: AAG    p: 13
## Training      subject: AAI    p: 13
## Training      subject: AAN    p: 13
## Training      subject: AAP    p: 13
## Training      subject: AAA    p: 14
## Training      subject: AAB    p: 14
## Training      subject: AAD    p: 14
## Training      subject: AAF    p: 14
## Training      subject: AAG    p: 14
## Training      subject: AAI    p: 14
## Training      subject: AAN    p: 14
## Training      subject: AAP    p: 14
## Training      subject: AAA    p: 15
## Training      subject: AAB    p: 15
## Training      subject: AAD    p: 15
## Training      subject: AAF    p: 15
## Training      subject: AAG    p: 15
## Training      subject: AAI    p: 15
## Training      subject: AAN    p: 15
## Training      subject: AAP    p: 15
## Training      subject: AAA    p: 16
## Training      subject: AAB    p: 16
## Training      subject: AAD    p: 16
## Training      subject: AAF    p: 16
## Training      subject: AAG    p: 16
## Training      subject: AAI    p: 16
## Training      subject: AAN    p: 16
## Training      subject: AAP    p: 16
## Training      subject: AAA    p: 17
## Training      subject: AAB    p: 17
## Training      subject: AAD    p: 17
## Training      subject: AAF    p: 17
## Training      subject: AAG    p: 17
## Training      subject: AAI    p: 17
## Training      subject: AAN    p: 17
## Training      subject: AAP    p: 17
## Training      subject: AAA    p: 18
## Training      subject: AAB    p: 18
## Training      subject: AAD    p: 18
## Training      subject: AAF    p: 18
## Training      subject: AAG    p: 18
## Training      subject: AAI    p: 18
## Training      subject: AAN    p: 18
## Training      subject: AAP    p: 18
## Training      subject: AAA    p: 19
## Training      subject: AAB    p: 19
## Training      subject: AAD    p: 19
## Training      subject: AAF    p: 19
## Training      subject: AAG    p: 19
## Training      subject: AAI    p: 19
## Training      subject: AAN    p: 19
## Training      subject: AAP    p: 19
## Training      subject: AAA    p: 20
## Training      subject: AAB    p: 20
## Training      subject: AAD    p: 20
## Training      subject: AAF    p: 20
## Training      subject: AAG    p: 20
## Training      subject: AAI    p: 20
## Training      subject: AAN    p: 20
## Training      subject: AAP    p: 20
## Training      subject: AAA    p: 21
## Training      subject: AAB    p: 21
## Training      subject: AAD    p: 21
## Training      subject: AAF    p: 21
## Training      subject: AAG    p: 21
## Training      subject: AAI    p: 21
## Training      subject: AAN    p: 21
## Training      subject: AAP    p: 21
## Training      subject: AAA    p: 22
## Training      subject: AAB    p: 22
## Training      subject: AAD    p: 22
## Training      subject: AAF    p: 22
## Training      subject: AAG    p: 22
## Training      subject: AAI    p: 22
## Training      subject: AAN    p: 22
## Training      subject: AAP    p: 22
## Training      subject: AAA    p: 23
## Training      subject: AAB    p: 23
## Training      subject: AAD    p: 23
## Training      subject: AAF    p: 23
## Training      subject: AAG    p: 23
## Training      subject: AAI    p: 23
## Training      subject: AAN    p: 23
## Training      subject: AAP    p: 23
## Training      subject: AAA    p: 24
## Training      subject: AAB    p: 24
## Training      subject: AAD    p: 24
## Training      subject: AAF    p: 24
## Training      subject: AAG    p: 24
## Training      subject: AAI    p: 24
## Training      subject: AAN    p: 24
## Training      subject: AAP    p: 24
## Training      subject: AAA    p: 25
## Training      subject: AAB    p: 25
## Training      subject: AAD    p: 25
## Training      subject: AAF    p: 25
## Training      subject: AAG    p: 25
## Training      subject: AAI    p: 25
## Training      subject: AAN    p: 25
## Training      subject: AAP    p: 25
## Training      subject: AAA    p: 26
## Training      subject: AAB    p: 26
## Training      subject: AAD    p: 26
## Training      subject: AAF    p: 26
## Training      subject: AAG    p: 26
## Training      subject: AAI    p: 26
## Training      subject: AAN    p: 26
## Training      subject: AAP    p: 26
## Training      subject: AAA    p: 27
## Training      subject: AAB    p: 27
## Training      subject: AAD    p: 27
## Training      subject: AAF    p: 27
## Training      subject: AAG    p: 27
## Training      subject: AAI    p: 27
## Training      subject: AAN    p: 27
## Training      subject: AAP    p: 27
## Training      subject: AAA    p: 28
## Training      subject: AAB    p: 28
## Training      subject: AAD    p: 28
## Training      subject: AAF    p: 28
## Training      subject: AAG    p: 28
## Training      subject: AAI    p: 28
## Training      subject: AAN    p: 28
## Training      subject: AAP    p: 28
## Training      subject: AAA    p: 29
## Training      subject: AAB    p: 29
## Training      subject: AAD    p: 29
## Training      subject: AAF    p: 29
## Training      subject: AAG    p: 29
## Training      subject: AAI    p: 29
## Training      subject: AAN    p: 29
## Training      subject: AAP    p: 29
## Training      subject: AAA    p: 30
## Training      subject: AAB    p: 30
## Training      subject: AAD    p: 30
## Training      subject: AAF    p: 30
## Training      subject: AAG    p: 30
## Training      subject: AAI    p: 30
## Training      subject: AAN    p: 30
## Training      subject: AAP    p: 30
## Training      subject: AAA    p: 31
## Training      subject: AAB    p: 31
## Training      subject: AAD    p: 31
## Training      subject: AAF    p: 31
## Training      subject: AAG    p: 31
## Training      subject: AAI    p: 31
## Training      subject: AAN    p: 31
## Training      subject: AAP    p: 31
## Training      subject: AAA    p: 32
## Training      subject: AAB    p: 32
## Training      subject: AAD    p: 32
## Training      subject: AAF    p: 32
## Training      subject: AAG    p: 32
## Training      subject: AAI    p: 32
## Training      subject: AAN    p: 32
## Training      subject: AAP    p: 32
## Training      subject: AAA    p: 33
## Training      subject: AAB    p: 33
## Training      subject: AAD    p: 33
## Training      subject: AAF    p: 33
## Training      subject: AAG    p: 33
## Training      subject: AAI    p: 33
## Training      subject: AAN    p: 33
## Training      subject: AAP    p: 33
## Training      subject: AAA    p: 34
## Training      subject: AAB    p: 34
## Training      subject: AAD    p: 34
## Training      subject: AAF    p: 34
## Training      subject: AAG    p: 34
## Training      subject: AAI    p: 34
## Training      subject: AAN    p: 34
## Training      subject: AAP    p: 34
## Training      subject: AAA    p: 35
## Training      subject: AAB    p: 35
## Training      subject: AAD    p: 35
## Training      subject: AAF    p: 35
## Training      subject: AAG    p: 35
## Training      subject: AAI    p: 35
## Training      subject: AAN    p: 35
## Training      subject: AAP    p: 35
## Training      subject: AAA    p: 36
## Training      subject: AAB    p: 36
## Training      subject: AAD    p: 36
## Training      subject: AAF    p: 36
## Training      subject: AAG    p: 36
## Training      subject: AAI    p: 36
## Training      subject: AAN    p: 36
## Training      subject: AAP    p: 36
## Training      subject: AAA    p: 37
## Training      subject: AAB    p: 37
## Training      subject: AAD    p: 37
## Training      subject: AAF    p: 37
## Training      subject: AAG    p: 37
## Training      subject: AAI    p: 37
## Training      subject: AAN    p: 37
## Training      subject: AAP    p: 37
## Training      subject: AAA    p: 38
## Training      subject: AAB    p: 38
## Training      subject: AAD    p: 38
## Training      subject: AAF    p: 38
## Training      subject: AAG    p: 38
## Training      subject: AAI    p: 38
## Training      subject: AAN    p: 38
## Training      subject: AAP    p: 38
## Training      subject: AAA    p: 39
## Training      subject: AAB    p: 39
## Training      subject: AAD    p: 39
## Training      subject: AAF    p: 39
## Training      subject: AAG    p: 39
## Training      subject: AAI    p: 39
## Training      subject: AAN    p: 39
## Training      subject: AAP    p: 39
## Training      subject: AAA    p: 40
## Training      subject: AAB    p: 40
## Training      subject: AAD    p: 40
## Training      subject: AAF    p: 40
## Training      subject: AAG    p: 40
## Training      subject: AAI    p: 40
## Training      subject: AAN    p: 40
## Training      subject: AAP    p: 40
## Training      subject: AAA    p: 41
## Training      subject: AAB    p: 41
## Training      subject: AAD    p: 41
## Training      subject: AAF    p: 41
## Training      subject: AAG    p: 41
## Training      subject: AAI    p: 41
## Training      subject: AAN    p: 41
## Training      subject: AAP    p: 41
## Training      subject: AAA    p: 42
## Training      subject: AAB    p: 42
## Training      subject: AAD    p: 42
## Training      subject: AAF    p: 42
## Training      subject: AAG    p: 42
## Training      subject: AAI    p: 42
## Training      subject: AAN    p: 42
## Training      subject: AAP    p: 42
## Training      subject: AAA    p: 43
## Training      subject: AAB    p: 43
## Training      subject: AAD    p: 43
## Training      subject: AAF    p: 43
## Training      subject: AAG    p: 43
## Training      subject: AAI    p: 43
## Training      subject: AAN    p: 43
## Training      subject: AAP    p: 43
## Training      subject: AAA    p: 44
## Training      subject: AAB    p: 44
## Training      subject: AAD    p: 44
## Training      subject: AAF    p: 44
## Training      subject: AAG    p: 44
## Training      subject: AAI    p: 44
## Training      subject: AAN    p: 44
## Training      subject: AAP    p: 44
## Training      subject: AAA    p: 45
## Training      subject: AAB    p: 45
## Training      subject: AAD    p: 45
## Training      subject: AAF    p: 45
## Training      subject: AAG    p: 45
## Training      subject: AAI    p: 45
## Training      subject: AAN    p: 45
## Training      subject: AAP    p: 45
## Training      subject: AAA    p: 46
## Training      subject: AAB    p: 46
## Training      subject: AAD    p: 46
## Training      subject: AAF    p: 46
## Training      subject: AAG    p: 46
## Training      subject: AAI    p: 46
## Training      subject: AAN    p: 46
## Training      subject: AAP    p: 46
## Training      subject: AAA    p: 47
## Training      subject: AAB    p: 47
## Training      subject: AAD    p: 47
## Training      subject: AAF    p: 47
## Training      subject: AAG    p: 47
## Training      subject: AAI    p: 47
## Training      subject: AAN    p: 47
## Training      subject: AAP    p: 47
## Training      subject: AAA    p: 48
## Training      subject: AAB    p: 48
## Training      subject: AAD    p: 48
## Training      subject: AAF    p: 48
## Training      subject: AAG    p: 48
## Training      subject: AAI    p: 48
## Training      subject: AAN    p: 48
## Training      subject: AAP    p: 48
## Training      subject: AAA    p: 49
## Training      subject: AAB    p: 49
## Training      subject: AAD    p: 49
## Training      subject: AAF    p: 49
## Training      subject: AAG    p: 49
## Training      subject: AAI    p: 49
## Training      subject: AAN    p: 49
## Training      subject: AAP    p: 49
## Training      subject: AAA    p: 50
## Training      subject: AAB    p: 50
## Training      subject: AAD    p: 50
## Training      subject: AAF    p: 50
## Training      subject: AAG    p: 50
## Training      subject: AAI    p: 50
## Training      subject: AAN    p: 50
## Training      subject: AAP    p: 50
```

```
## final model
treeda.final.asinh = treeda(
    sample_data(ps_sub)$PreImmpostPost,
    X,
    phy_tree(ps_sub),
    p = which.min(rowSums(cvloss)),
    check.consist = FALSE,
    A = A
)

## plot projections along discriminating axis
ggplot(data.frame(proj = treeda.final.asinh$projections, sample_data(ps_sub))) +
    geom_point(aes(x = proj, y = Subject, color = PreImmpostPost)) +
    scale_color_manual(values = preimmpost_cols) +
        xlab("Score")
```

```
## plot the coefficients along the tree
coorddf = data.frame(
    x = otu_position,
    y = as(treeda.final.asinh$leafCoefficients$beta, "matrix"),
    Genus = genusSubsetDA
)

rsvplot = ggplot(coorddf) +
  geom_hline(aes(yintercept = 0), color = "gray") +
  geom_point(aes(x = x, y = y, color = Genus), size = 1) +
  scale_color_manual(values = values) +
  theme(axis.title.x=element_blank(),
        axis.text.x=element_blank(),
        axis.ticks.x=element_blank()) +
  ylab("beta") +
  scale_x_continuous(limits = c(min(otu_position), max(otu_position)))
treeplot = plot_tree(phy_tree(ps_sub), ladderize = TRUE) +
    coord_flip() +
    scale_x_reverse()

fullplot = combine_plot_and_tree(
  rsvplot + theme(legend.position = "none"),
  treeplot,
  tree.height = 2
)
```

```
grid.arrange(
    fullplot,
    get_legend(rsvplot),
    ncol = 2,
    widths = c(1,.2)
)
```

## LEfSe - Linear Discriminant Effective Size (code not live)

```
ps.lefse <- ps

#   Keep only Pre and Immediate post samples - remove post samples
ps.lefse <- subset_samples(ps.lefse,PreImmpostPost%in%c("Pre",  "ImmPost"))

#   Filter taxa as done for SDA
ps.lefse <- prune_taxa(colSums(otu_table(ps.lefse) > 5) > 10, ps.lefse)

#   Transformation---just the relative abundance becasue we pass normalization factor when we call format.py
ps.lefse <- transform_sample_counts(ps.lefse,function(x){x/sum(x)})

#   Keep RSVs in rows and samples in columns
if(dim(otu_table(ps.lefse))[2]!=nsamples(ps.lefse)){otu_table(ps.lefse) <- t(otu_table(ps.lefse))}

#   Only consider RSV with at least known Phylum level
ps.lefse <- prune_taxa(taxa_names(ps.lefse)[!is.na(tax_table(ps.lefse)[,2])],ps.lefse)

#   Rename all the RSV with highest known level of taxonomy
tax.tab <- data.frame(tax_table(ps.lefse))

ModifyTax <- function(x,ind){
    #ind taxonomy level to change
    if(is.na(x[ind])){
        nonNa <- which(!is.na(x[-ind]))
        maxNonNa <- max(nonNa)
        #x[ind] <- paste(x[maxNonNa],".",x[ind])
        x[ind] <- paste(x[maxNonNa],".")
    }else{x[ind] <- x[ind]}
}

tax_table(ps.lefse)[,6] <- apply(tax.tab,1,ModifyTax,ind=6)
tax_table(ps.lefse)[,5] <- apply(tax.tab,1,ModifyTax,ind=5)
tax_table(ps.lefse)[,4] <- apply(tax.tab,1,ModifyTax,ind=4)
tax_table(ps.lefse)[,3] <- apply(tax.tab,1,ModifyTax,ind=3)
tax_table(ps.lefse)[,2] <- apply(tax.tab,1,ModifyTax,ind=2)

#   Rename RSV because existing names are too long
df.temp <- data.frame(Genus=tax_table(ps.lefse)[,6],".",num=seq(1,ntaxa(ps.lefse)))
renameRSV <- with(df.temp,paste(Genus,num))
taxa_names(ps.lefse) <- renameRSV
```

```
devtools::install_github("ying14/yingtools2")
library(yingtools2)
#   Prepare input for lefse
phy <- ps.lefse
#   Any class which defines the biological conditions
class <- "PreImmpostPost"
#   Any subclass which defines the biological conditions
subclass = NA;
#   If sample is different from subject
subject = "Subject";
#   set the alpha value for the Anova test (default 0.05)
anova.alpha = 0.05;
#   set the alpha value for the Wilcoxon test (default 0.05)
wilcoxon.alpha = 0.05;
#   set the threshold on the absolute value of the logarithmic LDA score (default 2.0)
lda.cutoff = 2;
#   verbose execution (default FALSE)
verbose.exe = FALSE;
#   wheter to perform the Wilcoxon step (default TRUE)
wilcoxon.within.subclass = FALSE;
#   select LDA or SVM for effect size (default LDA)
select.lda.svm = "lda";
#   whether to normalize the data in [0,1] for SVM feature waiting (default 1 strongly suggested)
normalize.for.svm = 1;
# set the number of bootstrap iteration for LDA (default 30)
num.boot = 30;
#   (for multiclass tasks) set whether the test is performed in a one-against-one ( 1 - more strict!) or in a one-against-all setting ( 0 - less strict) (default 0)
one.against.one = FALSE;
# set the multiple testing correction options. 0 no correction (more strict, default), 1 correction for independent comparisons, 2 correction for independent comparison
mult.test.correction = 1;
#   set the title of the analysis (default input file without extension)
set.title = "";
#   Do you want to make plot
make.lefse.plots = TRUE;
#   Do you want to do test on RSV level - if FALSE LEfse will look up significance at other taxonomy levels such as Phylum, Class, Order, Family, Genus
by_otus = FALSE;
#   Taxa names to be used in the plot
levels = rank_names(phy)
# set the normalization value (default -1.0 meaning no normalization)

#   just edit devtools::install_github("ying14/yingtools2")
    keepvars <- c(class, subclass, subject, "sample")
    keepvars <- unique(keepvars[!is.na(keepvars)])
    #   class, subclass variables from phyloseq sample data
    samp <- get.samp(phy)[, keepvars]
    #   consider statistical test for each RSV
    if (by_otus) {
        otu <- get.otu.melt(phy, sample_data = FALSE)
        otu.levels <- otu %>% mutate(taxon = otu) %>% group_by(sample,taxon) %>% summarize(pctseqs = sum(pctseqs)) %>%
            mutate(taxon = gsub(" ", "_", taxon))
    }else {# consider statistical test for each taxonomy level
        otu <- get.otu.melt(phy, sample_data = FALSE)
        otu.list <- lapply(1:length(levels), function(i) {
            lvls <- levels[1:i]
            lvl <- levels[i]
            otu.level <- otu
            otu.level$taxon <- do.call(paste, c(lapply(lvls,
                                                       function(l) otu[[l]]), sep = "|"))
            otu.level$rank <- lvl
            otu.level2 <- otu.level %>% group_by(sample, taxon,
                                                 rank) %>% summarize(pctseqs = sum(pctseqs)) %>%
                ungroup()
            return(otu.level2)
        })
        otu.levels <- bind_rows(otu.list) %>% mutate(taxon = gsub(" ",
                                                                  "_", taxon))
    }

otu.tbl <- otu.levels %>% dcast(sample ~ taxon, value.var = "pctseqs",
                                    fill = 0)
                          %>% left_join(samp, by = "sample")
                          %>% select_(.dots = c(keepvars,
                                    lazyeval::interp(~everything())))

    if (is.na(subject) | subject != "sample") {
        otu.tbl <- otu.tbl %>% dplyr::select(-sample)
    }

    tbl <- otu.tbl %>% t()

    #   Can use lefse.txt in Galaxy app as an input
    write.table(tbl, "lefse.txt", quote = FALSE, sep = "\t",
                col.names = FALSE)
    #   set arguments for calling format_input.py
    opt.class <- paste("-c", which(keepvars %in% class))
    opt.subclass <- ifelse(is.na(subclass), "", paste("-s", which(keepvars %in% subclass)))
    opt.subject <- ifelse(is.na(subject), "", paste("-u", which(keepvars %in% subject)))
    format.command <- paste("python format_input.py lefse.txt lefse.in",opt.class, opt.subclass, opt.subject, "-o 10000000")
    system(format.command)

    #   Prepare a command to run in the terminal
    #   lefse.in --the input file (produced by calling format.py command in the terminal)
    #   lefse.res --the output file containing the data for the visualization module
    lefse.command <- paste("python run_lefse.py lefse.in lefse.res",
                           "-a", anova.alpha,
                           "-w", wilcoxon.alpha,
                           "-l", lda.cutoff,
                           "-e", as.numeric(wilcoxon.within.subclass),
                           "-y", as.numeric(one.against.one),
                           "-s", mult.test.correction)

    system(lefse.command)
    print("Wrote lefse.res")

    #   If you want the output file in tabular format
    lefse.out <- read.table("lefse.res", header = FALSE, sep = "\t")
    lefse.out <- lefse.out %>% dplyr::rename(taxon = V1, log.max.pct = V2, direction = V3,lda = V4, p.value = V5)

    #   If we want to make plots
    if (make.lefse.plots) {
        #   plot effect size
        system("python plot_res.py lefse.res lefse_lda.pdf --format pdf")
        print("Wrote lefse_lda.pdf")
        #   plot cladogram
        system("python plot_cladogram.py lefse.res lefse_clado.pdf --format pdf")
        print("Wrote lefse_clado.pdf")
    }
```
